# Supplementary material for: An intranasal adenoviral-vectored vaccine protects against highly pathogenic avian influenza H5N1 in naive and antigen-experienced animals
Source: Cell Rep Med. 2026 Jan 30;7(2):102582. doi: 10.1016/j.xcrm.2025.102582 (PMC12923973; doi:10.1016/j.xcrm.2025.102582)
Supplement: Document S1. Figures S1–S9 and Tables S1 and S2 [file mmc1.pdf]

Cell Reports Medicine, Volume *7*

Supplemental information

An intranasal adenoviral-vectored vaccine protects against highly pathogenic avian influenza H5N1 in naive and antigen-experienced animals

Baoling Ying, Kelly Pyles, Tamarand L. Darling, Kuljeet Seehra, Truc Pham, Lin-Chen Huang, Houda H. Harastani, Ashish Sharma, Pritesh Desai, Elena A. Kashentseva, David T. Curiel, Bjoern Peters, James Brett Case, Eva-Maria Strauch, Michael S. Diamond, and Adrianus C.M. Boon

Supplemental information

An intranasal adenoviral-vectored vaccine protects against highly pathogenic avian influenza H5N1 in naïve and antigen-experienced animals

Baoling Ying, Kelly Pyles, Tamarand L Darling, Kuljeet Seehra, Truc Pham, Lin-Chen Huang, Houda H. Harastani, Ashish Sharma, Pritesh Desai, Elena A. Kashentseva, David T. Curiel, Bjoern Peters, James Brett Case, Eva-Maria Strauch, Michael S. Diamond, and Adrianus C.M. Boon

Supplementary Figure S1

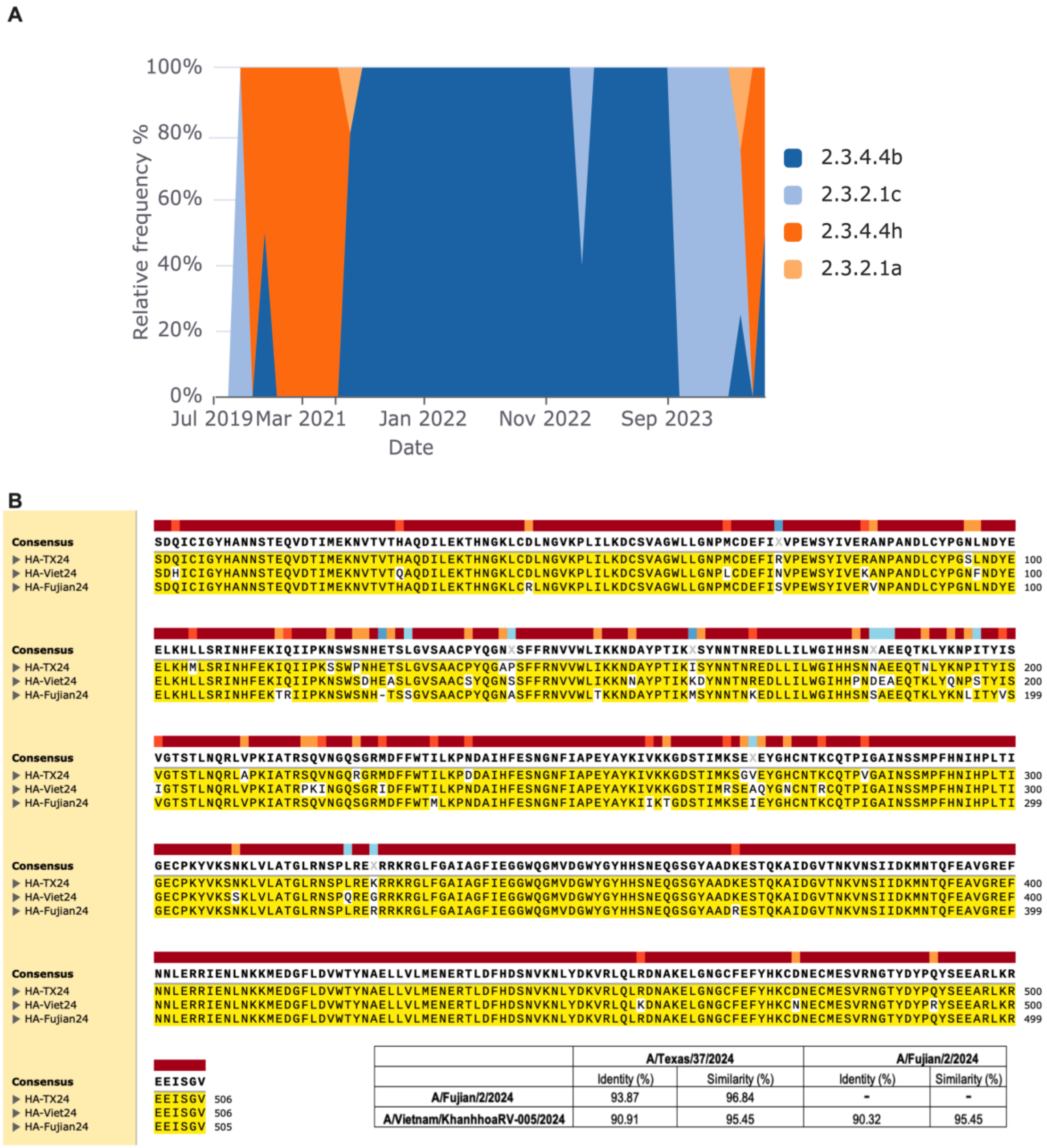

	A/Texas/37/2024		A/Fujian/2/2024	
	Identity (%)	Similarity (%)	Identity (%)	Similarity (%)
A/Fujian/2/2024	93.87	96.84	-	-
A/Vietnam/KhanhhoaRV-005/2024	90.91	95.45	90.32	95.45

Figure S1. Genetic and sequence analysis of HA proteins from recently circulating H5-Texas, H5-Vietnam and H5-Fujian strains, Related to Figure 1. (**A**) Relative clade frequency of human infecting HPAI H5 influenza A viruses circulating between June 2021 and April 2024. (**B**) Sequence alignment of HA proteins from the H5-Texas, H5-Vietnam, and H5-Fujian strains. The percentages of identity and similarity among the strains are shown in the Table.

Supplementary Figure S2

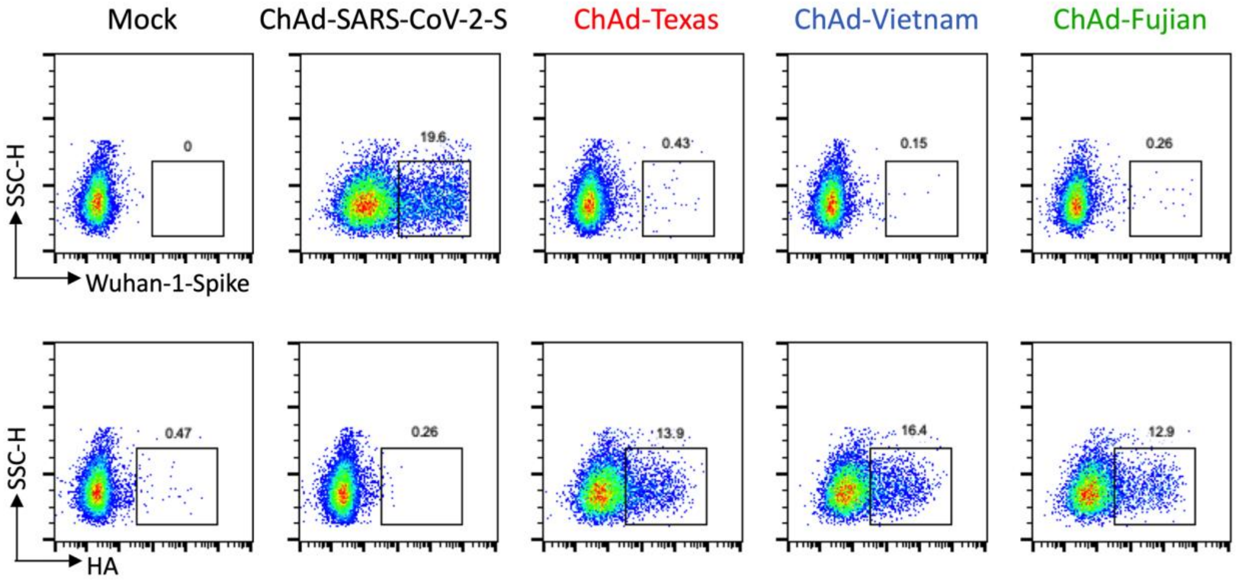

Figure S2. Surface expression of H5 HA from ChAd-Texas (clade 2.3.4.4b), ChAd-Vietnam (clade 2.3.2.1c) and ChAd-Fujian (clade 2.3.4.4h) transduced 293T cells, Related to Figures 1 and 2. 293T cells were transduced with 20 virus particles per cell of ChAd-SARS-CoV-2-S, ChAd-Texas, ChAd-Vietnam or ChAd-Fujian. At 40 h post-infection, cells were fixed and stained with an oligoclonal pool of spike-reactive monoclonal antibodies SARS2-2, SARS2-11, SARS2-16, SARS2-31, SARS2-38, SARS2-57, and SARS2-71 SARS-CoV-2 (VanBlargan et al., 2021), or HA stem-reactive mAb (CR9114). Representative flow cytometry plots of surface staining are shown.

Supplementary Figure S3

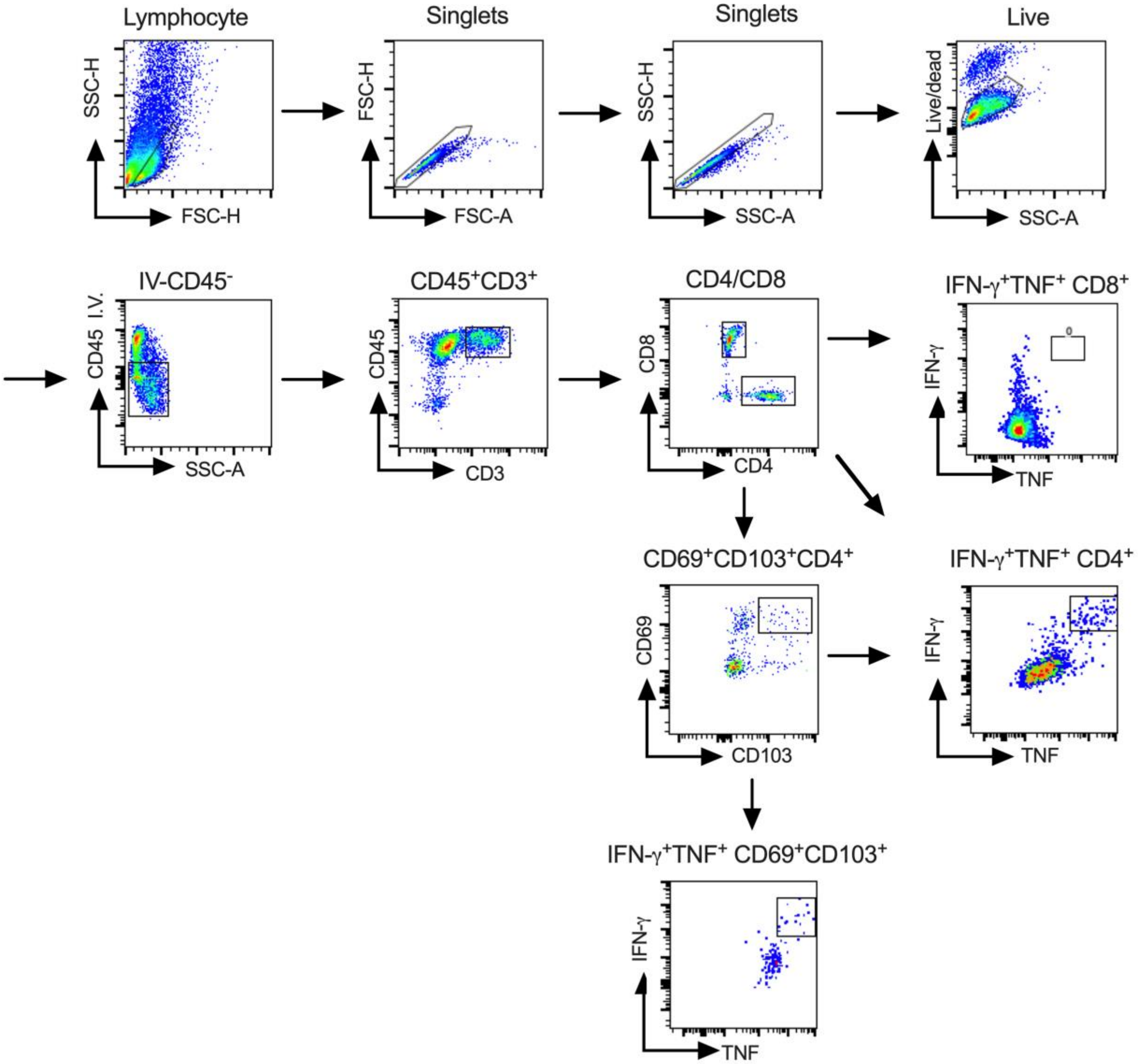

Figure S3. Gating strategy for T cell responses, Related to Figure 3 and S6. Seven-week-old female C57BL/6 mice were immunized with ChAd-CTRL or ChAd-Texas and boosted four weeks later. T cell responses in the spleen and lungs at day 10 post-boost were analyzed after HA peptide re-stimulation. Cells were gated for lymphocytes (FSC-A/SSC-A), singlets (SSC-W/SSC-H), live cells (Viability dye eF506$^-$), IV-CD45$^-$, CD45$^+$ CD3$^+$, CD4$^+$ or CD8$^+$ followed by CD4$^+$ or CD8$^+$ cell populations expressing IFN-γ and TNF. HA-specific resident memory CD4$^+$ T cells (CD4 T_{RM}) in the lung were gated on CD4$^+$CD69$^+$CD103$^+$, followed by staining of IFN-γ $^+$ and TNF$^+$.

Supplementary Figure S4

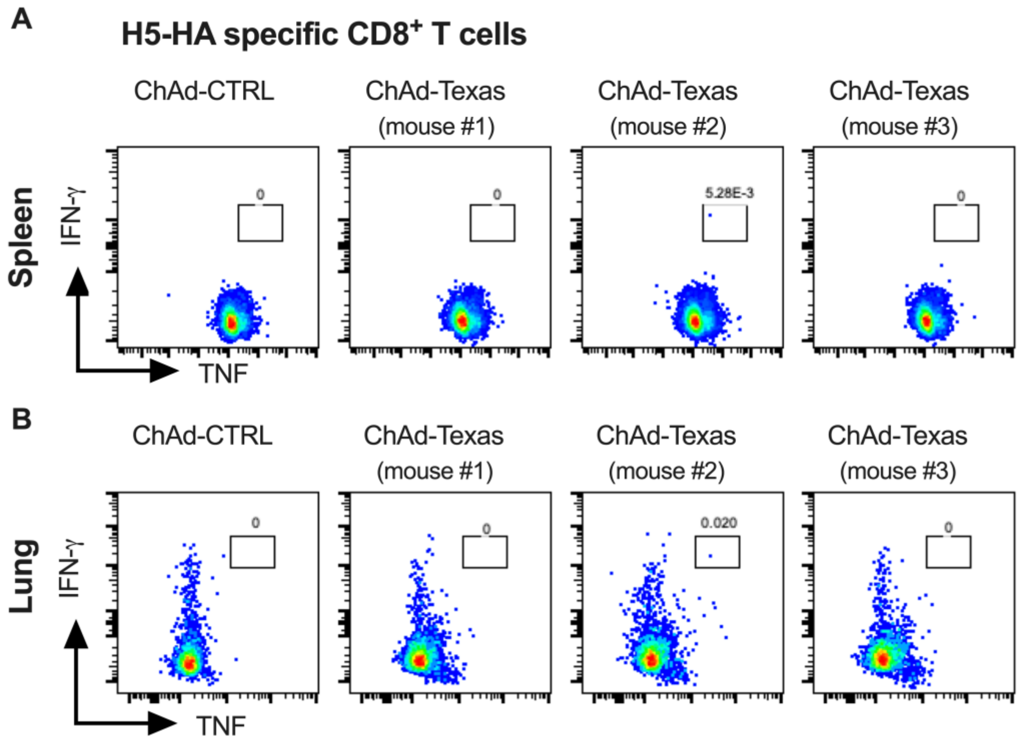

Figure S4. Lack of measurable CD8^{+} T cell responses following IN immunization with ChAd-Texas vaccine in C57BL/6 mice, Related to Figure 3. Seven-week-old female C57BL/6 mice were immunized IN with 10^{9} vp of ChAd-CTRL or ChAd-Texas and boosted four weeks later. On day 10 post-boost, CD8^{+} T cell responses in the spleen and lungs were analyzed following HA peptide re-stimulation. Representative flow cytometry plots of IFN-γ^{+} and TNF^{+} CD8^{+} T cells in the spleen (**A**) and lung (**B**) are shown.

Supplementary Figure S5

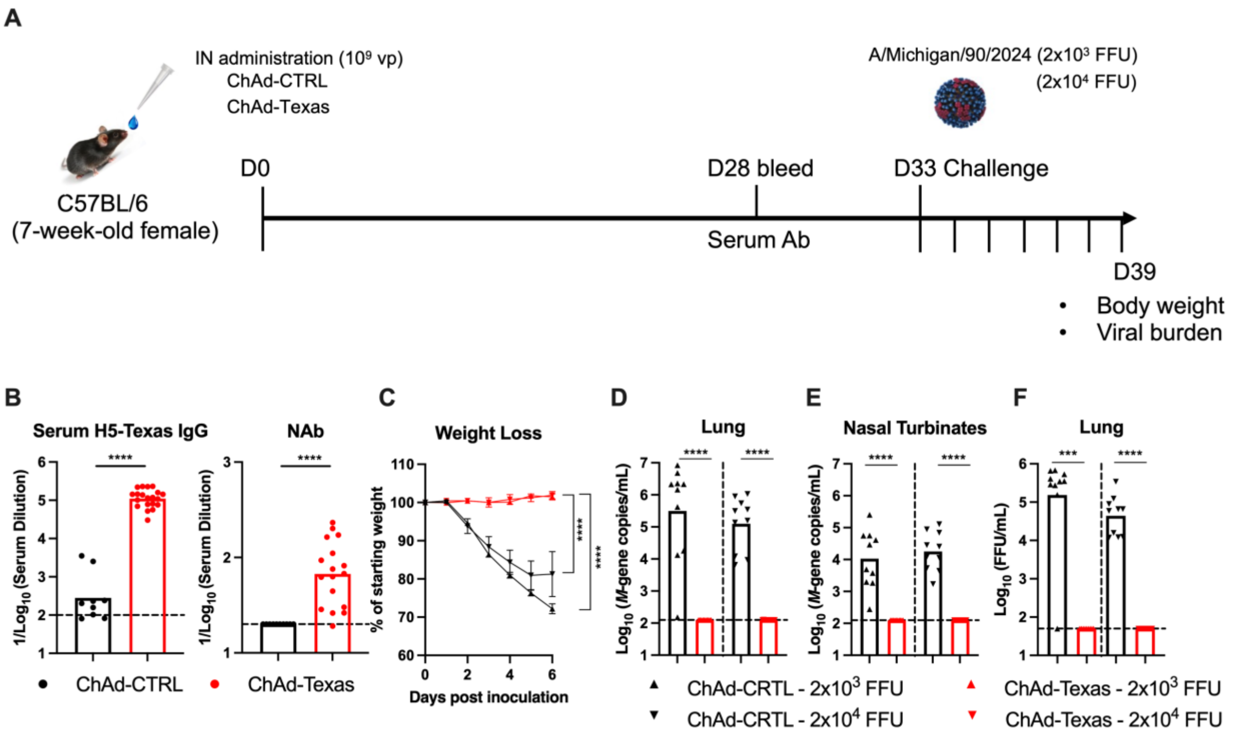

Figure S5. IN delivered ChAd-Texas vaccine confers protection against high dose H5N1 challenge, Related to Figure 4. (**A**) Scheme of immunizations, blood collection, and virus challenge. Cohorts of C57B/L6 mice were immunized via an IN route with 10^9 vp of ChAd-CTRL or ChAd-Texas. Sera were collected four weeks after immunization, and H5-Texas specific IgG and neutralizing antibody titers were determined (**B**). Four to five weeks after immunization, mice were challenged IN with increasing doses (2,000 or 20,000 FFU) of H5N1 A/Michigan/90/2024**.** (**C**) Body weight change. Viral RNA levels were determined at 6 dpi in the lungs (**D**) and nasal turbinates (**E**). Infectious virus levels in the lung (**F**). n = 8-10, two experiments, boxes illustrate geometric mean values, dotted lines show the limit of detection. Statistical analysis: (**B, D-F**) Mann-Whitney test. (**C**) two-way ANOVA analysis between ChAd-CTRL and ChAd-Texas immunized mice at corresponding challenge dose: **** $P < 0.0001$.

Supplementary Figure S6

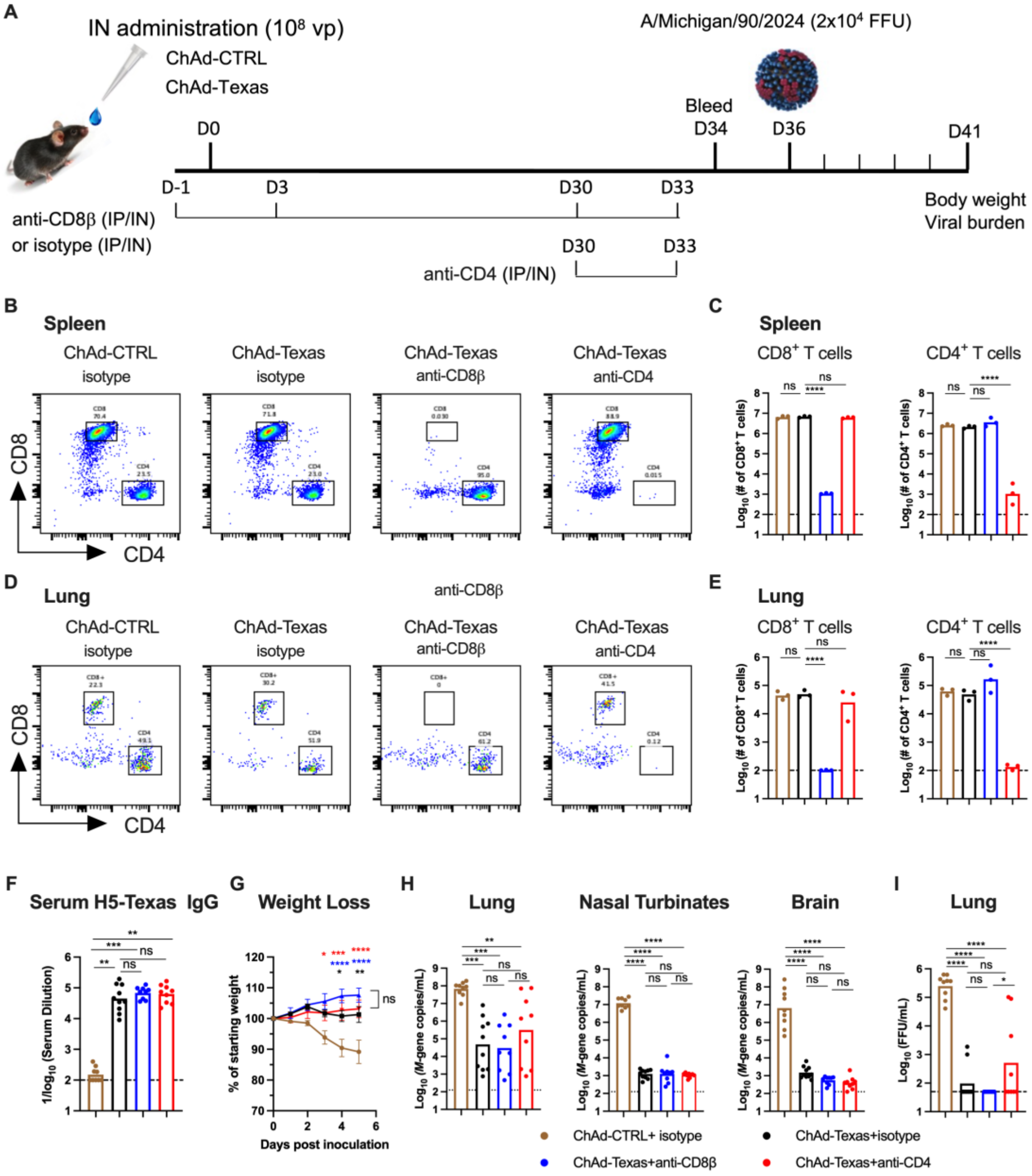

Figure S6. Depletion of CD4⁺ or CD8⁺ T cells had minimal impact on ChAd-Texas-mediated protection following H5N1 challenge, Related to Figures 3 and 4. (**A**) Scheme of immunizations, T cell depletion, and virus challenge. On day 34 post-immunization, spleens (**B-C**) and lungs (**D-E**) from a subset of animals were collected for analysis of $CD8^+$ and $CD4^+$ T cell frequencies and numbers by flow cytometry. Representative scatter plots (**B, D**) and total cell numbers (**C, E**) of $CD8^+$ and $CD4^+$ T cells in the spleen (**B-C**) and lungs (**D-E**) are shown (n = 3 per group; boxes indicate geometric mean values). (**F**) Serum IgG against HA of A/Texas/37/2024. (**G**) Weight loss following intranasal challenge with A/Michigan/90/2024. (**H**) Viral RNA levels in lungs, nasal turbinates, and brain. (**I**) Infectious virus titers in lungs (**F-I**, n = 8-10; two independent experiments). Boxes indicate geometric mean values; dotted lines represent the limit of detection. Statistical analysis: (**C, E**) one-way ANOVA with Dunnett's post-test; (**F**) Kruskal-Wallis with Dunn's test; (**G**) two-way ANOVA with Turke's post-test; (**H** and **I**) one-way ANOVA with Holm-Sidak post-test. ns, not significant, * $P < 0.05$; ** $P < 0.01$; *** $P < 0.001$; **** $P < 0.0001$.

Supplementary Figure S7

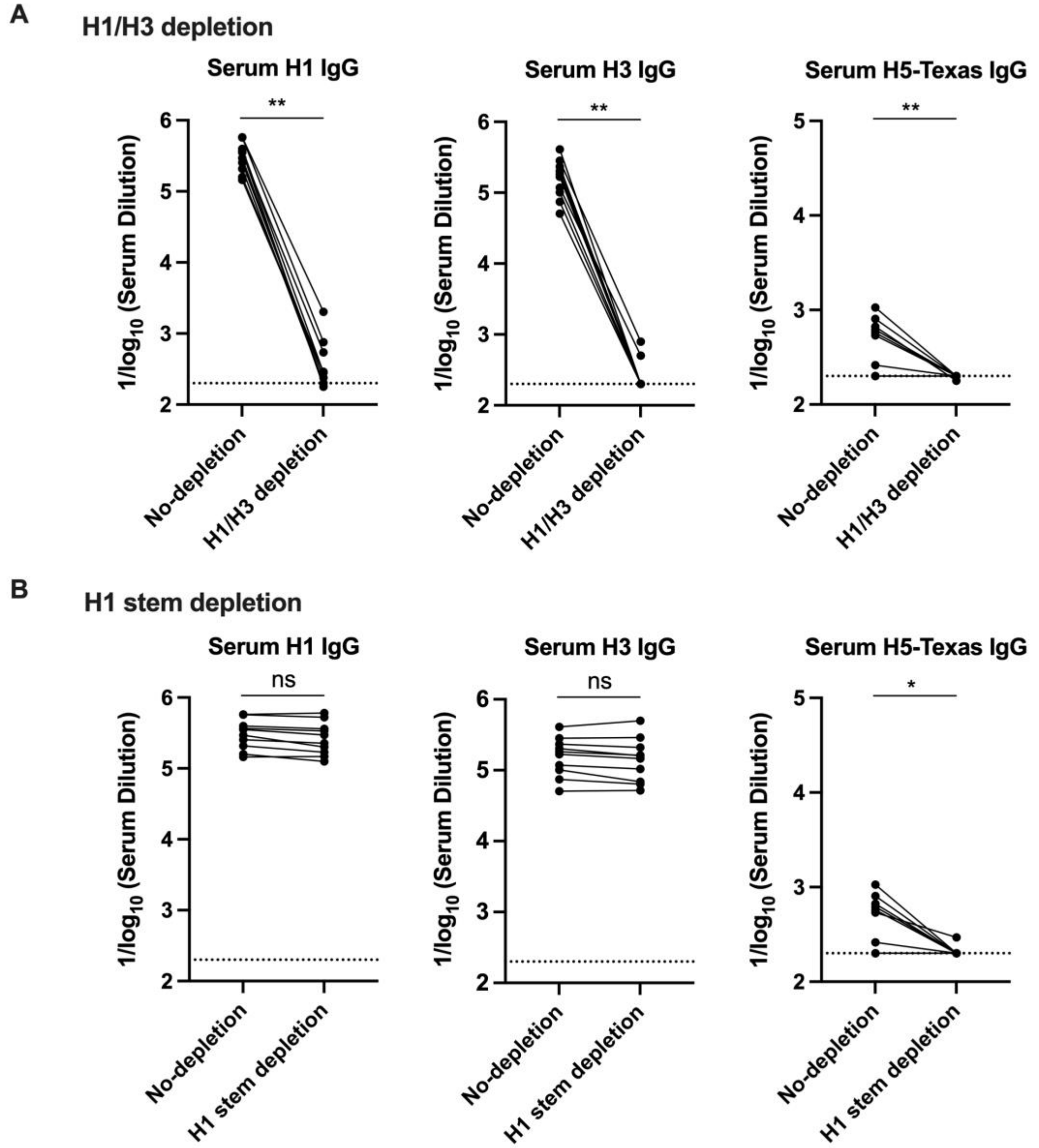

Figure S7. QIV immunization elicits low levels of H5-HA cross-reactive antibodies, Related to Figure 6. Serum samples collected three weeks after the second QIV immunization were absorbed with streptavidin-coated magnetic beads loaded with biotinylated HA proteins from H1, H3, or the stem domain of H1 HA. The pre-cleared sera were evaluated for residual binding to H1-, H3-, and H5-HA detection beads. Serum IgG levels binding to H1-HA, H3-HA, or H5-HA following pre-clearing with empty beads (no depletion), H1 and H3-HA loaded beads (H1/H3 depletion, panel **A**), or H1 HA stem–loaded beads (H1 stem depletion, panel **B**). Connecting lines represent sera from the same animals. Dotted lines indicate the limit of detection (LOD); values at the LOD are plotted slightly below the line. Wilcoxon matched pairs signed rank test. ns, not significant; * $P < 0.05$; ** $P < 0.01$.

Supplementary Figure S8

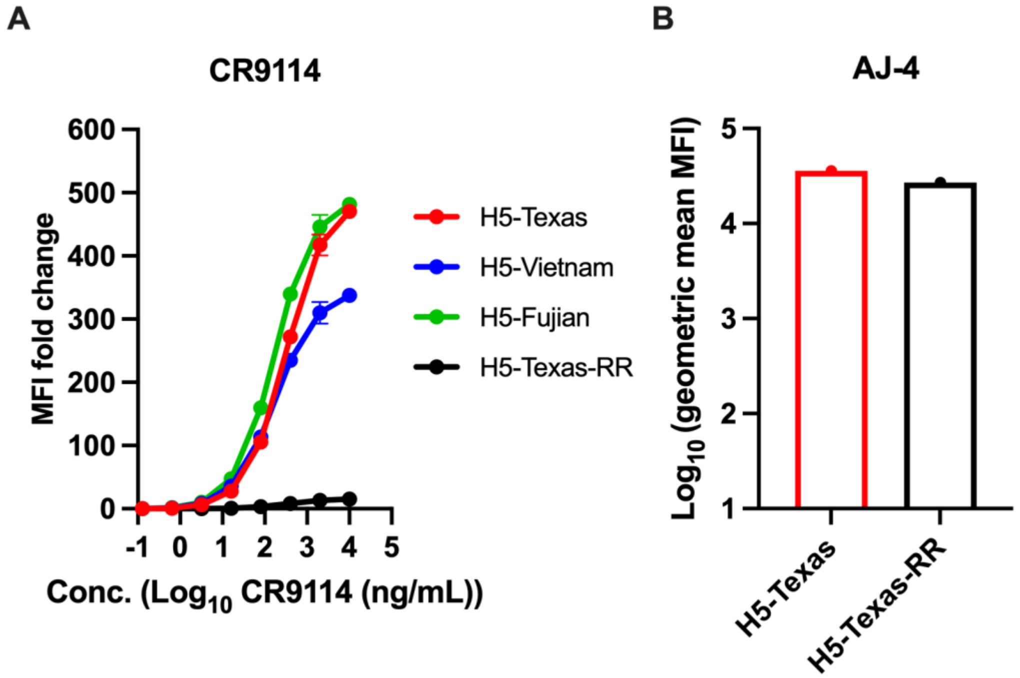

Figure S8. Elimination of the stem epitope in H5 HA, Related to Figure 6. Substitutions at residues 45 and 49 in the stem-domain of H5 HA (I45R-T49R) disrupt CR9114 binding as measured by flow-cytometry (**A**). Anti-H5 antibody (AJ-4), targeting the head domain of H5 was used to control for H5 HA binding (**B**).

Supplementary Figure S9

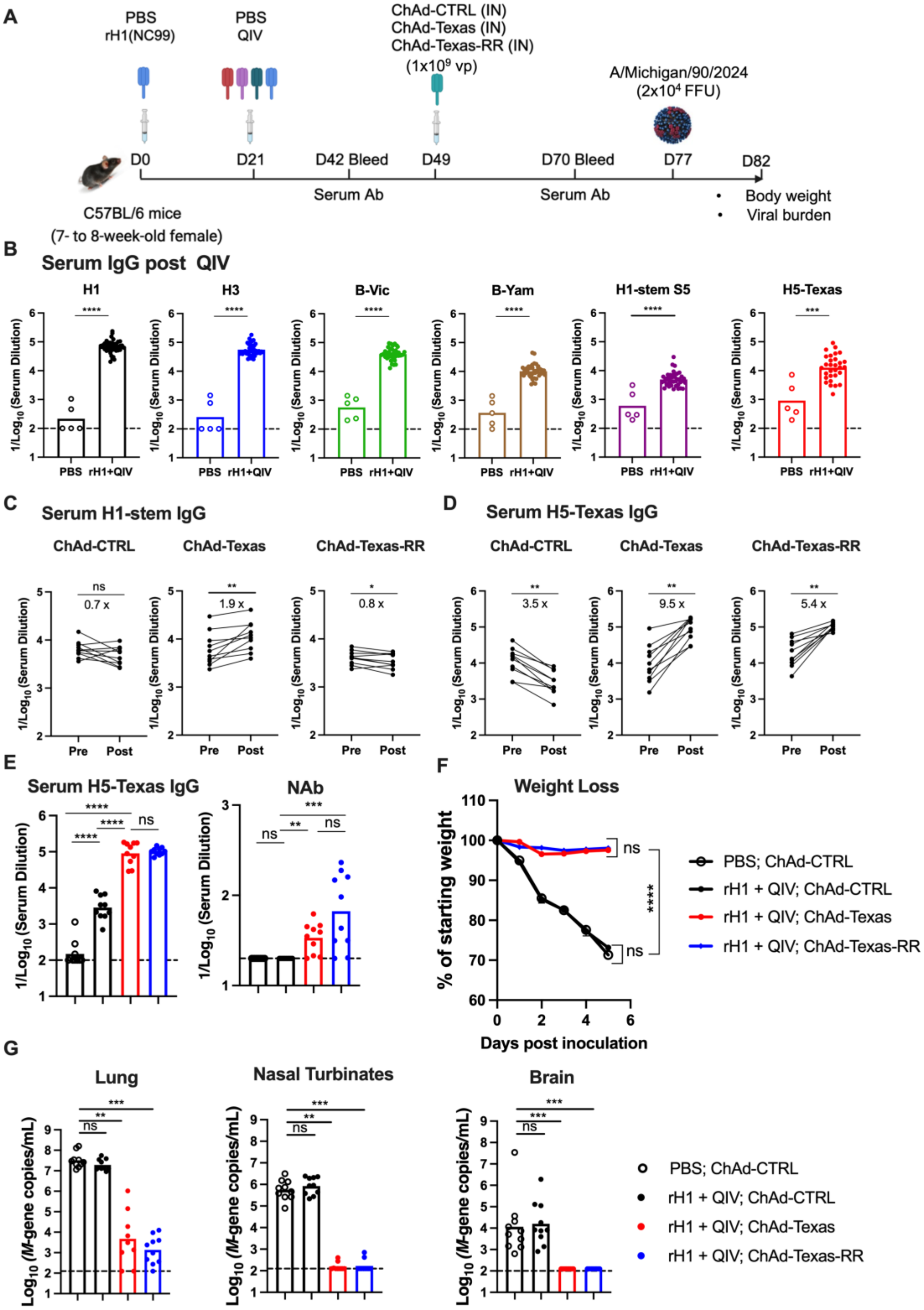

Figure S9. ChAd-Texas induces H5-specific immunity and confers protection against H5N1 in mice immunized with H1 and H3 HA antigens, Related to Figure 6. (**A**) Schematic of study design with indicated timeline of immunization, blood collection, and viral challenge. C57BL/6 mice were immunized IM with either PBS or 3.0 μg of recombinant H1 HA protein (A/New Caledonia/20/1999, H1N1) mixed 1:1 with AddaVax followed by seasonal QIV. Three weeks after the QIV dose, mice received an IN immunization of 10^9 vp of ChAd-CTRL, ChAd-Texas, or ChAd-Texas-RR vaccine. Serum samples were collected three weeks after QIV vaccine and three weeks after the ChAd-vaccine boost to evaluate antibody responses. (**B**) Serum IgG against HA of seasonal influenza virus strain and H5N1-Texas at day 21 after QIV immunization. (**C**) Paired analysis of serum IgG titers of pre- and post-boost with indicated ChAd-vaccine against H1 HA-stem protein. (**D**) Paired analysis of serum IgG titers pre- and post-boost with indicated ChAd-vaccine against H5 HA-Texas. (**E**) Serum H5-HA binding IgG and neutralizing antibody titers after ChAd-vaccine boost in previously unprimed or QIV primed mice. (**F**) Weight loss after IN challenge with 20,000 FFU of A/Michigan/90/2024. (**G**) Viral RNA levels determined at 4 dpi in lungs, nasal turbinates and brain. n = 9-10 mice per group, two independent experiments. Column heights indicate geometric mean values, and dotted lines show the limit of detection. Fold-changes are indicated above corresponding graphs. Statistical analyses: Mann–Whitney test (**B, E and G**); (**C-D**) Wilcoxon matched signed-rank test. (**F**) two-way ANOVA or mixed effect analysis with the Geisser-Greenhouse correction. ns, not significant; * $P < 0.05$, ** $P < 0.01$, *** $P < 0.001$, **** $P < 0.0001$.

Supplementary Table S1: HA protein sequences, Related to Figure 1, 3, 4, 5 and 6.

H1 A/Delaware/55/2019

MKAILVVMLYTFTTANADTLCIGYHANNSTDTVDTVLEKNVTVTHSVNLLEDKHNGKLCKLRGVA
PLHLGKCNIAGWILGNPECESLSTARSWSYIVETSNSDNGTCYPGDFINYEELREQLSSVSSFE
RFEIFPKTSSWPNHDSDNGVTAACPHAGAKSFYKNLIWLVKKGKSYPKINQTYINDKGKEVLVL
WGIHHPPTIADQQSLYQNADAYVFVGTSRYSKMFKPEIATRPKVRDQEGRMNYYWTLVEPGDK
ITFEATGNLVAPRYAFTMERDAGSGIIISDTPVHDCNTTCQTPEGAINTSLPFQNVHPITIGKCPKY
VKSTKLRLATGLRNVPSIQSRGLFGAIAGFIEGGWTGMVDGWYGYHHQNEQGSGYAADLKST
QNAIDKITNKVNSVIEKMNTQFTAVGKEFNHLEKRIENLNKKVDDGFLDIWTYNAELLVLLENERT
LDYHDSNVKNLYEKVRNQLKNNAKEIGNGCFEFYHKCDNTCMESVKNGTYDYPKYSEEAKLN
REKIDGGYIPEAPRDGQAYVRKDGEWVLLSTFLGSGLNDIFEAQKIEWHEGHHHHHH

H3 A/Darwin/11/2021

MKTIIALSNILCLVFAQKIPGNDNSTATLCLGHHAVPNGTIVKTITNDRIEVTNATELVQNSSIGEIC
DSPHQILDGGNCTLIDALLGDPQCDGFQNKEWDLFVERSRANSNCYPYDVPDYASLRSLVASS
GTLEFKNESFNWTGVKQNGTSSACIRGSSSSFFSRLNWLTSLNNIYPAQNVTMPNKEQFDKLYI
WGVHHPDTDKNQISLFAQSSGRITVSTKRSQQAVIPNIGSRPRIRDIPSRISIYWTIVKPGDILLIN
STGNLIAPRGYFKIRSGKSSIMRSDAPIGKCKSECITPNGSIPNDKPFQNVNRITYGACPRYVKQ
STLKLATGMRNVPEKQTRGIFGAIAGFIENGWEGMVDGWYGFRHQNSEGRGQAADLKSTQAA
IDQINGKLNRLIGKTNEKFHQIEKEFSEVEGRVQDLEKYVEDTKIDLWSYNAELLVALENQHTIDL
TDSEMNKLFEKTKKQLRENAEDMGNGCFKIYHKCDNACIGSIRNETYDHNVYRDEALNNRFQI
KGVGYIPEAPRDGQAYVRKDGEWVLLSTFLGSGLNDIFEAQKIEWHEGHHHHHH

Victoria-lineage B/Singapore/WUH4618/2021

MKAIIVLLMVVTSNADRICTGITSSNSPHVVKTATQGEVNVTGVIPLTTTPTKSHFANLKGTETRG
KLCPKCLNCTDLDVALGRPKCTGKIPSARVSILHEVRPVTSGCFPIMHDRTKIRQLPNLLRGYEH
VRLSTHNVINTEDAPGGPYEIGTSGSCLNITNGKGFFATMAWAVPKNKTATNPLTIEVPYICTEEE
DQITVWGFHSDDETQMVRLYGDSKPQKFTSSANGVTTHYVSQIGGFPNQTEDGGLPQSGRIV
VDYMVQKSGKTGTITYQRGILLPQKVWCASGKSKVIKGSLPLIGEADCLHEKYGGLNKSKPYYT
GEHAKAIGNCPIWVKTPLKLANGTKYRPPAKLLKERGFFGAIAGFLEGGWEGMIAGWHGYTSH
GAHGVAVAADLKSTQEAINKITKNLNSLSELEVKNLQRLSGAMDELHNEILELDEKVDDLRADTIS
SQIELAVLLSNEGIINSEDEHLLALERKLKKMLGPSAVEIGNGCFETKHKCNQTCLDRIAAGTFDA
GEFSLPTFDSLNITAASGYIPEAPRDGQAYVRKDGEWVLLSTFLGSGLNDIFEAQKIEWHEGHH
HHHH

Yamagata-lineage B/Singapore/INFTT-16-0610/2016

MKAIIVLLMVVTSNADRICTGITSSNSPHVVKTATQGEVNVTGVIPLTTTPTKSYFANLKGTRTRG
KLCPDCLNCTDLDVALGRPMCVGTTPSAKASILHEVRPVTSGCFPIMHDRTKIRQLPNLLRGYE
KIRLSTQNVIDAEKAPGGPYRLGTSGSCPNATSKIGFFSTMAWAVPKDNYKNATNPQTVEVPYI
CTEGEDQITVWGFHSDNKTQMKSLYGDSNPQKFTSSANGVTTHYVSQIGDFPDQTEDGGLPQ
SGRIVVDYMVQKPGKTGTIVYQRGVLLPQKVWCASGRSKVIKGSLPLIGEADCLHEEYGGLNK
SKPYYTGKHAKAIGNCPIWVKTPLKLANGTKYRPPAKLLKERGFFGAIAGFLEGGWEGMIAGW
HGYTSHGAHGVAVAADLKSTQEAINKITKNLNSLSELEVKNLQRLSGAMDELHNEILELDEKVDD
LRADTISSQIELAVLLSNEGIINSEDEHLLALERKLKKMLGPSAVDIGNGCFETKHKCNQTCLDRI
AAGTFNAGEYSLPTFDSLNITAASGYIPEAPRDGQAYVRKDGEWVLLSTFLGSGLNDIFEAQKIE
WHEGHHHHHH

H5 A/Vietnam/KhanhhoaRV1-005/2024

MERIVLLFATINLVKSDHICIGYHANNSTEQVDTIMEKNVTVTQAQDILEKTHNGKLCDLNGVKPL
ILKDCSVAGWLLGNPLCDEFINVPEWSYIVEKANPANDLCYPGNFNDYEELKHLLSRINHFEKIQ
IIPKNSWSDHEASLGVSAACSYQGNSSFFRNVVWLIKKNNAYPTIKKDYNNTNREDLLILWGIHH
PNDEAEQTKLYQNPSTYISIGTSTLNQRLVPKIATRPKINGQSGRIDFFWTILKPNDAIHFESNGN
FIAPEYAYKIVKKGDSTIMRSEAQYGNCNTRCQTPIGAINSSMPFHNIHPLTIGECPKYVKSSKLV
LATGLRNSPQREGRRKRGLFGAIAGFIEGGWQGMVDGWYGYHHSNEQGSGYAADKESTQKA
IDGVTNKVNSIIDKMNTQFEAVGREFNNLERRIENLNKKMEDGFLDVWTYNAELLVLMENERTL
DFHDSNVKNLYDKVRLQLKDNAKELGNGCFEFYHKCNNECMESVRNGTYDYPRYSEEARLKR
EEISGVGYIPEAPRDGQAYVRKDGEWVLLSTFLGHHHHHH

H5 A/Texas/37/2024

MENIVLLLAIVSLVKSDQICIGYHANNSTEQVDTIMEKNVTVTHAQDILEKTHNGKLCDLNGVKPL
ILKDCSVAGWLLGNPMCDEFIRVPEWSYIVERANPANDLCYPGSLNDYEELKHMLSRINHFEKI
QIIPKSSWPNHETSLGVSAACPYQGAPSFFRNVVWLIKKNDAYPTIKISYNNTNREDLLILWGIH
HSNNAEEQTNLYKNPITYISVGTSTLNQRLAPKIATRSQVNGQRGRMDFFWTILKPDDAIHFES
NGNFIAPEYAYKIVKKGDSTIMKSGVEYGHCNTKCQTPVGAINSSMPFHNIHPLTIGECPKYVKS
NKLVLATGLRNSPLREKRRKRGLFGAIAGFIEGGWQGMVDGWYGYHHSNEQGSGYAADKES
TQKAIDGVTNKVNSIIDKMNTQFEAVGREFNNLERRIENLNKKMEDGFLDVWTYNAELLVLMEN
ERTLDFHDSNVKNLYDKVRLQLRDNAKELGNGCFEFYHKCDNECMESVRNGTYDYPQYSEEA
RLKREEISGVGYIPEAPRDGQAYVRKDGEWVLLSTFLGHHHHHH

H5 A/Texas/37/2024-I45R-T49R

MENIVLLLAIVSLVKSDQICIGYHANNSTEQVDTIMEKNVTVTHAQDILEKTHNGKLCDLNGVKPL
ILKDCSVAGWLLGNPMCDEFIRVPEWSYIVERANPANDLCYPGSLNDYEELKHMLSRINHFEKI
QIIPKSSWPNHETSLGVSAACPYQGAPSFFRNVVWLIKKNDAYPTIKISYNNTNREDLLILWGIH
HSNNAEEQTNLYKNPITYISVGTSTLNQRLAPKIATRSQVNGQRGRMDFFWTILKPDDAIHFES
NGNFIAPEYAYKIVKKGDSTIMKSGVEYGHCNTKCQTPVGAINSSMPFHNIHPLTIGECPKYVKS
NKLVLATGLRNSPLREKRRKRGLFGAIAGFIEGGWQGMVDGWYGYHHSNEQGSGYAADKES
TQKARDGVRNKVNSIIDKMNTQFEAVGREFNNLERRIENLNKKMEDGFLDVWTYNAELLVLME
NERTLDFHDSNVKNLYDKVRLQLRDNAKELGNGCFEFYHKCDNECMESVRNGTYDYPQYSEE
ARLKREEISGVGYIPEAPRDGQAYVRKDGEWVLLSTFLGHHHHHH

H5 A/Fujian/2/2024

MEKIVLLLSVVNLVKSDQICIGYHANNSTEQVDTIMEKNVTVTHAQDILEKTHNGKLCRLNGVKP
LILKDCSVAGWLLGNPMCDEFISVPEWSYIVERVNPANDLCYPGNLNDYEELKHLLSRINHFEKT
RIIPKNSWSNHTSSGVSAACPYQGNASFFRNVVWLTKKNDAYPTIKMSYNNTNKEDLLILWGIH
HSNSAEEQTKLYKNLITYVSVGTSTLNQRLVPKIATRSQVNGQSGRMDFFWTMLKPNDAIHFES
NGNFIAPEYAYKIIKTGDSTIMKSEIEYGHCNTKCQTPIGAINSSMPFHNIHPLTIGECPKYVKSNK
LVLATGLRNSPLRERRRKRGLFGAIAGFIEGGWQGMVDGWYGYHHSNEQGSGYAADRESTQ
KAIDGVTNKVNSIIDKMNTQFEAVGREFNNLERRIENLNKKMEDGFLDVWTYNAELLVLMENER
TLDFHDSNVKNLYDKVRLQLRDNAKELGNGCFEFYHKCDNECMESVRNGTYDYPQYSEEARL
KREEISGVGYIPEAPRDGQAYVRKDGEWVLLSTFLGHHHHHH

Signal peptide
Foldon
Avi-tag
6x histag

Supplementary Table S2, Related to Figure 3.

Predicted MHC class I HA peptides in C57BL/6 mice by NetMHCPan 4.1 EL

allele	start	end	length	peptide	score	percentile_rank
H-2-Db	303	313	11	SSMPFHNIHPL	0.925328	0.01
H-2-Db	136	145	10	SSWPNHETSL	0.826912	0.02
H-2-Db	304	313	10	SMPFHNIHPL	0.670663	0.04
H-2-Db	445	453	9	VLMENERTL	0.554872	0.06
H-2-Db	11	19	9	VSLVKSDQI	0.521853	0.07
H-2-Db	158	166	9	SFFRNVVWL	0.414976	0.11
H-2-Db	321	329	9	YVKSNKLVL	0.411489	0.11
H-2-Db	109	117	9	GSLNDYEEL	0.395589	0.12
H-2-Kb	303	310	8	SSMPFHNI	0.94821	0.01
H-2-Kb	411	418	8	VGREFNNL	0.835582	0.02
H-2-Kb	155	163	9	GAPSFFRNV	0.774067	0.03
H-2-Kb	264	272	9	IAPEYAYKI	0.595342	0.07
H-2-Kb	208	216	9	KNPITYISV	0.415406	0.18
H-2-Kb	525	532	8	SVGTYQIL	0.279533	0.31
H-2-Kb	156	163	8	APSFFRNV	0.267409	0.33
H-2-Kb	137	145	9	SWPNHETSL	0.263779	0.33

Predicted MHC class II HA peptides in C57BL/6 mice by NetMHCPan 4.1 EL

allele	start	end	length	peptide	score	percentile_rank
H-2-IAb	204	218	TNLYKNPITYISVGT	0.8314	0.29	204
H-2-IAb	168	182	KKNDAYPTIKISYNN	0.4958	1.8	168
H-2-IAb	90	104	PEWSYIVERANPAND	0.491	1.8	90
H-2-IAb	303	317	SSMPFHNIHPLTIGE	0.4054	2.5	303
H-2-IAb	452	466	TLDFHDSNVKNLYDK	0.3664	2.8	452
H-2-IAb	531	545	ILSIYSTAASSLALA	0.3492	3	531
H-2-IAb	298	312	VGAINSSMPFHNIHP	0.2841	3.9	298
H-2-IAb	210	224	PITYISVGTSTLNQR	0.2478	4.6	210
H-2-IAb	344	358	KRGLFGAIAGFIEGG	0.2372	4.7	344
H-2-IAb	364	378	DGWYGYHHSNEQGSG	0.2299	4.9	364
H-2-IAb	366	380	WYGYHHSNEQGSGYA	0.1943	5.6	366
H-2-IAb	498	512	RNGTYDYPQYSEEAR	0.1675	6.5	498
H-2-IAb	265	279	APEYAYKIVKKGDST	0.1453	7.3	265
H-2-IAb	37	51	EKNVTVTHAQDILEK	0.1368	7.6	37
