# Supplementary material for: An intranasal adenoviral-vectored vaccine protects against highly pathogenic avian influenza H5N1 in naive and antigen-experienced animals
Source: Cell Rep Med. 2026 Jan 30;7(2):102582. doi: 10.1016/j.xcrm.2025.102582 (PMC12923973; doi:10.1016/j.xcrm.2025.102582)
Supplement: Document S2. Article plus supplemental information [file mmc2.pdf]

Cell Reports Medicine

Article

An intranasal adenoviral-vectored vaccine protects against highly pathogenic avian influenza H5N1 in naive and antigen-experienced animals

Graphical abstract

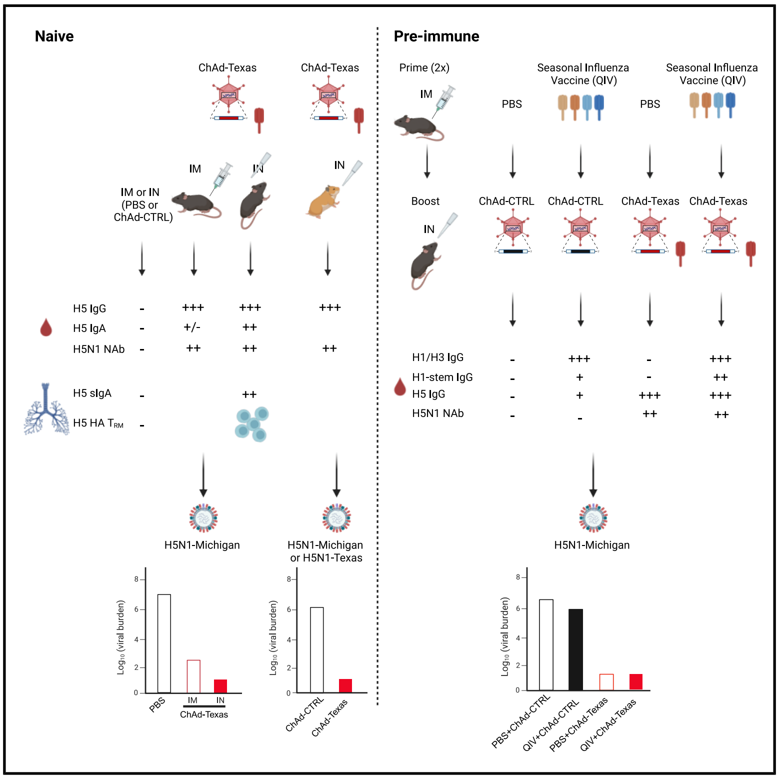

Highlights

- IN-delivered ChAd-Texas vaccine elicits mucosal antibody and T cell responses
- IN-delivered ChAd-Texas vaccine protects against H5N1 in mice and hamsters
- IN delivery of ChAd-Texas vaccine confers greater protection than IM delivery
- ChAd-Texas induces H5N1 immunity in the setting of prior influenza immunity

Authors

Baoling Ying, Kelly Pyles, Tamarand L. Darling, ..., Eva-Maria Strauch, Michael S. Diamond, Adrianus C.M. Boon

Correspondence

mdiamond@wustl.edu (M.S.D.), jboon@wustl.edu (A.C.M.B.)

In brief

Ying et al. demonstrate that intranasally delivered ChAd-Texas H5 vaccine elicits robust systemic and mucosal immunity that confers protection against homologous H5N1 challenge in rodents. Intranasal immunization outperforms intramuscular delivery and is effective even with pre-existing immunity from seasonal influenza vaccines, highlighting its potential as a vaccine strategy against H5N1.

Ying et al., 2026, Cell Reports Medicine 7, 102582
February 17, 2026 © 2026 The Authors. Published by Elsevier Inc.
https://doi.org/10.1016/j.xcrm.2025.102582

CellPress

Cell Reports Medicine

CellPress
OPEN ACCESS

Article

An intranasal adenoviral-vectored vaccine protects against highly pathogenic avian influenza H5N1 in naive and antigen-experienced animals

Baoling Ying,[1] Kelly Pyles,[1] Tamarand L. Darling,[1] Kuljeet Seehra,[1] Truc Pham,[1] Lin-Chen Huang,[1] Houda H. Harastani,[1] Ashish Sharma,[1] Pritesh Desai,[1] Elena A. Kashentseva,[2] David T. Curiel,[2] Bjoern Peters,[3] James Brett Case,[1] Eva-Maria Strauch,[1,5,6] Michael S. Diamond,[1,4,5,7,8,*] and Adrianus C.M. Boon[1,4,5,7,9,*]

[1]Department of Medicine, Washington University School of Medicine, St. Louis, MO 63103, USA
[2]Department of Radiation Oncology, Washington University School of Medicine, St. Louis, MO 63103, USA
[3]Center for Infectious Disease and Vaccine Research, La Jolla Institute for Immunology, La Jolla, CA 92037, USA
[4]Department of Pathology & Immunology, Washington University School of Medicine, St. Louis, MO 63103, USA
[5]Department of Molecular Microbiology, Washington University School of Medicine, St. Louis, MO 63103, USA
[6]Department of Biochemistry and Biophysics, Washington University School of Medicine, St. Louis, MO 63103, USA
[7]Andrew M. and Jane M. Bursky the Center for Human Immunology and Immunotherapy, Washington University School of Medicine, St. Louis, MO 63103, USA
[8]Center for Vaccines and Immunity to Microbial Pathogens, Washington University School of Medicine, St. Louis, MO 63103, USA
[9]Lead contact
*Correspondence: mdiamond@wustl.edu (M.S.D.), jboon@wustl.edu (A.C.M.B.)
https://doi.org/10.1016/j.xcrm.2025.102582

SUMMARY

The emergence of highly pathogenic avian H5N1 influenza viruses in dairy cows and humans has increased the potential for another pandemic. To address this risk, we developed chimpanzee adenoviral (ChAd)-vectored H5 hemagglutinin-targeted vaccines and tested their immunogenicity and efficacy in rodents. Immunization with ChAd-Texas (clade 2.3.4.4b) vaccine in mice elicits neutralizing antibody responses and confers protection against viral infection and mortality upon challenge with a human H5N1 isolate (A/Michigan/90/2024, clade 2.3.4.4b). Intranasal delivery of the ChAd-Texas vaccine elicits mucosal antibody and T cell responses and confers greater protection than intramuscular immunization. In Syrian hamsters, a single intranasal dose of ChAd-Texas vaccine prevents weight loss and reduces airway infection after H5N1 A/Michigan/90/2024 or A/Texas/37/2024 challenge. Importantly, prior seasonal influenza vaccination does not impair antibody responses or protection after intranasal delivery of the ChAd-Texas vaccine. These results support the development of mucosally administered ChAd-Texas HA vaccines as an effective platform for HPAI H5N1 preparedness.

INTRODUCTION

H5N1 avian influenza, a highly pathogenic strain of the influenza A virus (HPAI), poses a threat to the poultry and livestock industry and human public health. Since its emergence in 1997,[1–4] H5N1 virus has undergone genetic evolution, driven largely by reassortments and mutations in key viral proteins, particularly those involved in host receptor binding and immune evasion.[5–11] This genetic diversification has led to the emergence of multiple clades and subclades (clades 1, 2.2, 2.3.2, and 2.3.4.4) with varying pathogenic potential and geographic distributions.[7,12–14] In 2021, a new subclade, 2.3.4.4b, emerged and became dominant in both Europe and North America, resulting in fatal infections in many species.[11,14–17] In March 2024, H5N1 clade 2.3.4.4b virus infection was confirmed in dairy cows in Texas and then disseminated throughout many regions of the United States.[18–21] Since 2024, H5N1 has also caused over 70 human cases in the United States including two fatalities. The continued circulation of H5N1 viruses increases the potential for reassortment with other influenza viruses and adaptation to animal and human hosts. Thus, it is critical to develop vaccines that mitigate the threat of a future pandemic of H5N1 and other related influenza strains.

Currently, the US government has stockpiled pre-pandemic H5N1 vaccines targeting strains that circulated two decades ago.[22–24] These vaccines include Sanofi Pasteur's inactivated vaccine (A/Vietnam/1203/2004, clade 1), Seqirus' MF59 adjuvanted inactivated vaccine AUDENZ (A/turkey/Turkey/1/2005 NIBRG-23, clade 2.2.1), and GlaxoSmithKline's AS03 adjuvanted monovalent vaccine (A/Indonesia/5/2005, clade 2.1.3). Although these vaccines derived from clade 1 and clade 2.1 H5N1 strains induce cross-reactive neutralizing antibodies against the H5 clade 2.3.4.4b A/Astrakhan/3212/2020 strain,[25] their efficacy against the currently circulating H5N1 clade 2.3.4.4b strain, A/Texas/37/2024, has not been established.

Preclinical studies using various intramuscularly (IM) administered mRNA lipid nanoparticles (mRNA-LNP) and replicating RNA vaccines targeting clade 2.3.4.4b H5N1 virus have

© 2026 The Authors. Published by Elsevier Inc.
This is an open access article under the CC BY license (http://creativecommons.org/licenses/by/4.0/).

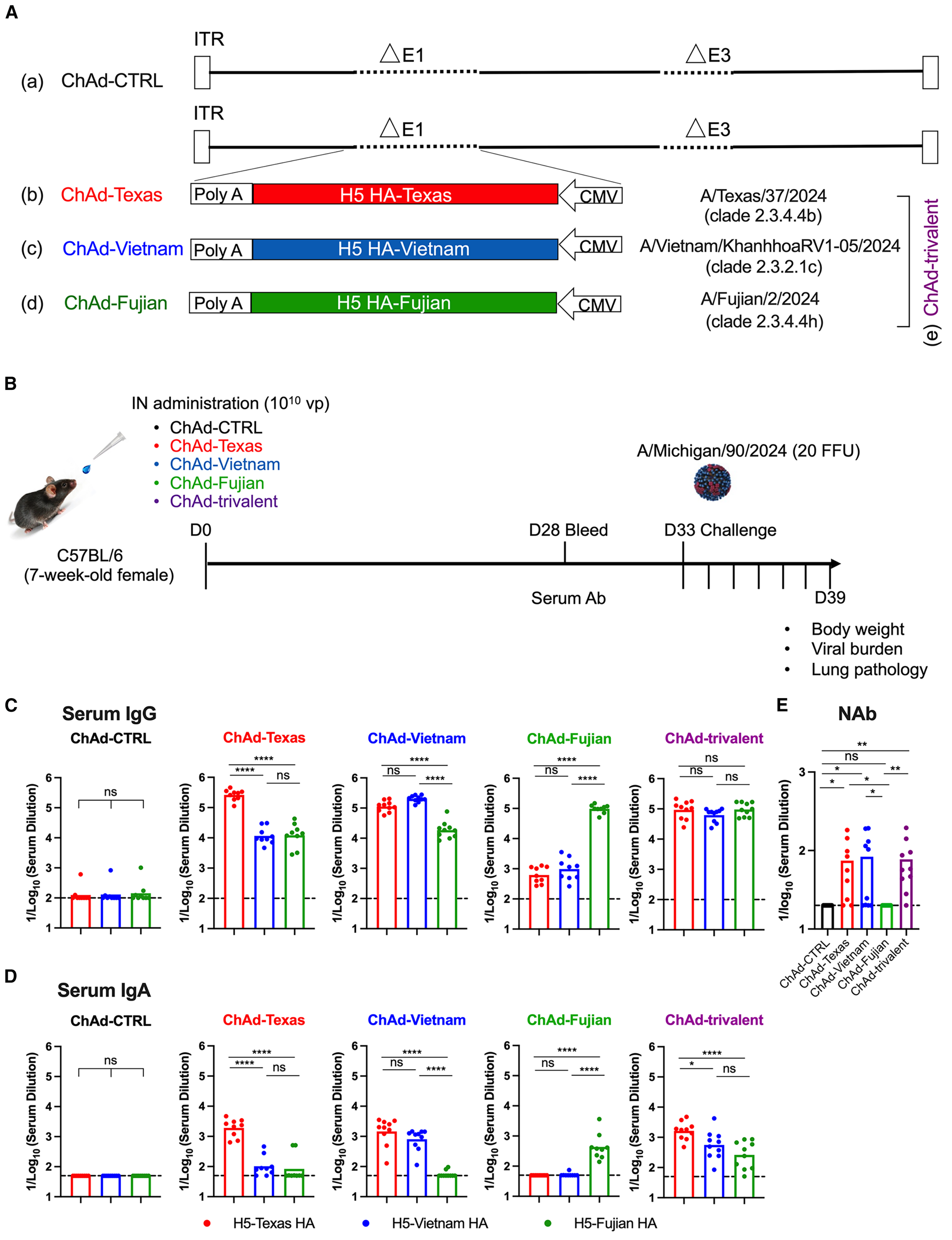

(legend on next page)

demonstrated protection against infection by different H5N1 isolates in small animal models.[26–28] How these results translate to humans remains to be determined, including whether they will prevent upper airway infection and transmission. Previous reports have shown that intranasal (IN), but not IM, immunization elicits mucosal immune responses and provides superior protection against respiratory virus infections and transmission, including SARS-CoV-2 and seasonal influenza.[29–32] Thus, nasal or inhaled vaccines against H5N1 might protect better against infection and transmission than IM vaccines.[33,34]

Pre-existing immunity from prior seasonal influenza infection or vaccination can influence the efficacy of H5N1 vaccines. An individual's first exposure to influenza A virus (IAV) imprints the immune system and shapes subsequent responses to related strains.[35–38] In individuals with pre-existing immunity to seasonal influenza viruses, vaccination with H5 antigens can elicit a broadly reactive HA stem-directed antibody response, but a weak H5 head-specific response; however, a robust H5 head-directed response can emerge after iterative boosting.[39] Thus, developing vaccines that can overcome immune imprinting is an important goal.[40]

In this study, we generated ChAd-vectored vaccines against HPAI H5 influenza viruses, encoding different H5 HA proteins from clades 2.3.4.4b, 2.3.4.4h, and 2.3.2.1c H5N1 viruses based on circulating viruses that had infected humans (GISAID April 2024, Figure S1).[41] We evaluated systemic and mucosal immune responses after single-dose IN delivery of the vaccine and assessed protective efficacy against homologous virus challenge in mice and Syrian hamsters. Finally, we evaluated antibody responses and efficacy of the ChAd-H5 vaccine in the context of pre-existing seasonal influenza virus immune responses.

RESULTS

Antibody responses after immunization with intranasally delivered ChAd-H5 vaccines

We constructed a series of ChAd-vectored H5 vaccines based on a simian Ad-36 virus. These replication-incompetent ChAd-H5 vectors encode full-length hemagglutinin (HA) proteins corresponding to A/Texas/37/2024 (ChAd-Texas, clade 2.4.4.4b), A/Vietnam/KhanhhoaRV1-005/2024 (ChAd-Vietnam, clade 2.3.2.1c), and A/Fujian/2/2024 (ChAd-Fujian, clade 2.3.4.4h). The ChAd-control (ChAd-CTRL) lacks a transgene (Figure 1A). Expression of H5 HA protein was confirmed using an HA stem-reactive monoclonal antibody (CR9114) (Figure S2). To assess immunogenicity, we tested the vaccine as monovalent (ChAd-Texas, ChAd-Vietnam, or ChAd-Fujian) or trivalent (1:1:1 mixture of ChAd-Texas, ChAd-Vietnam, and ChAd-Fujian) formulations by immunizing mice once via the IN route with a total of 10^{10} virus particles (vp) (Figure 1B). Serum samples were collected 4 weeks after immunization, and IgG and IgA responses against HA proteins of H5-Texas, H5-Vietnam, and H5-Fujian strains (Table S1) were measured (Figures 1C and 1D). The monovalent ChAd-vectored vaccines induced robust IgG responses against the homologous H5 HA proteins, with variable reactivity against the heterologous clades. Mice immunized with ChAd-Texas had high serum IgG titers against H5-Texas (Figure 1C, geometric mean titer [GMT]: 261,477) but 10- to 100-fold lower levels against the H5-Vietnam and H5-Fujian. Similarly, mice immunized with ChAd-Vietnam developed high IgG titers against H5-Vietnam (Figure 1C, GMT: 203,190), with slightly lower titers against H5-Texas and H5-Fujian. In comparison, mice immunized with the ChAd-Fujian had robust IgG titers against H5-Fujian (GMT: 97,229), but 100- to 150-fold lower IgG titers against H5-Texas and H5-Vietnam. Mice immunized with ChAd-trivalent vaccine elicited high IgG titers against all three H5 proteins (H5-Texas, GMT: 92,865; H5-Vietnam, GMT: 62,458; H5-Fujian, GMT: 96,229). Serum IgA responses (Figure 1D) against the H5 HA proteins were generally 100 to 1,000 times lower than IgG titers. The reactivity of the IgA against the different H5 HA proteins after immunization with ChAd-Texas, ChAd-Vietnam, ChAd-Fujian, or ChAd-trivalent recapitulated the pattern observed with serum IgG (Figure 1C).

We assessed the serum neutralizing activity using a focus-reduction neutralization test against a low-pathogenic reassortant H5N1 virus (PR8/H5N1), which encodes six gene segments of A/Puerto Rico/8/1934 and the HA and NA gene segments of the H5N1 A/Michigan/90/2024 strain (PR8/H5N1) (Figure 1E). Immunization with ChAd-Texas, ChAd-Vietnam, or the ChAd-trivalent vaccine induced serum antibodies that neutralized PR8/H5N1 virus in most mice. However, ChAd-Fujian elicited poorly neutralizing antibody responses against PR8/H5N1, with titers below the assay limit of detection (Figure 1E).

ChAd-vectored vaccines protect against lethal challenge by contemporary H5N1 virus

To assess the efficacy of the different IN-delivered ChAd-vectored vaccines, the mice from Figure 1 were challenged IN with 20 focus-forming units (FFU) (~20 LD_{50}) of H5N1 A/Michigan/90/2024 (Figure 1B). Whereas substantial weight loss (17%–20%) was observed within 6 days of infection in ChAd-CTRL-immunized mice (Figure 2A), the ChAd-Texas, ChAd-Vietnam and trivalent ChAd-vectored vaccines all prevented weight loss. Some weight loss (~7%) was observed in the monovalent ChAd-Fujian vaccinated mice. As expected, at 6 days post-infection (dpi), ChAd-CTRL vaccinated mice had

Figure 1. Murine serum antibody responses following IN immunization with monovalent or trivalent ChAd-vectored H5 vaccines

(A) Diagram of ChAd-vectored H5 HA vaccines encoding the HA protein from different highly pathogenic avian influenza H5 viruses, including ChAd-Texas (A/Texas/37/2024, clade 2.3.4.4b), ChAd-Vietnam (A/Vietnam/KhanhhoaRV1-005/2024, clade 2.3.2.1c), and ChAd-Fujian (A/Fujian/2/2024, clade 2.3.4.4h).

(B–D) (B) Scheme of immunizations, blood collection, and virus challenge. C57BL/6 mice were immunized once via an IN route with ChAd-CTRL, ChAd-Texas, ChAd-Vietnam, ChAd-Fujian, or ChAd-trivalent vaccine; sera were collected four weeks after immunization; and HA-specific IgG (C) and IgA (D) levels were determined.

(E) Serum neutralizing antibody titers were determined against A/Michigan/90/2024 (PR8/H5N1).

Results in this figure are from two experiments (n = 8–10); boxes illustrate geometric mean titers, and dotted lines show the limit of detection.

Statistical analysis: one-way ANOVA with Tukey's post-test (C–E): ns, not significant, $*p < 0.05$, $**p < 0.01$, $****p < 0.0001$.

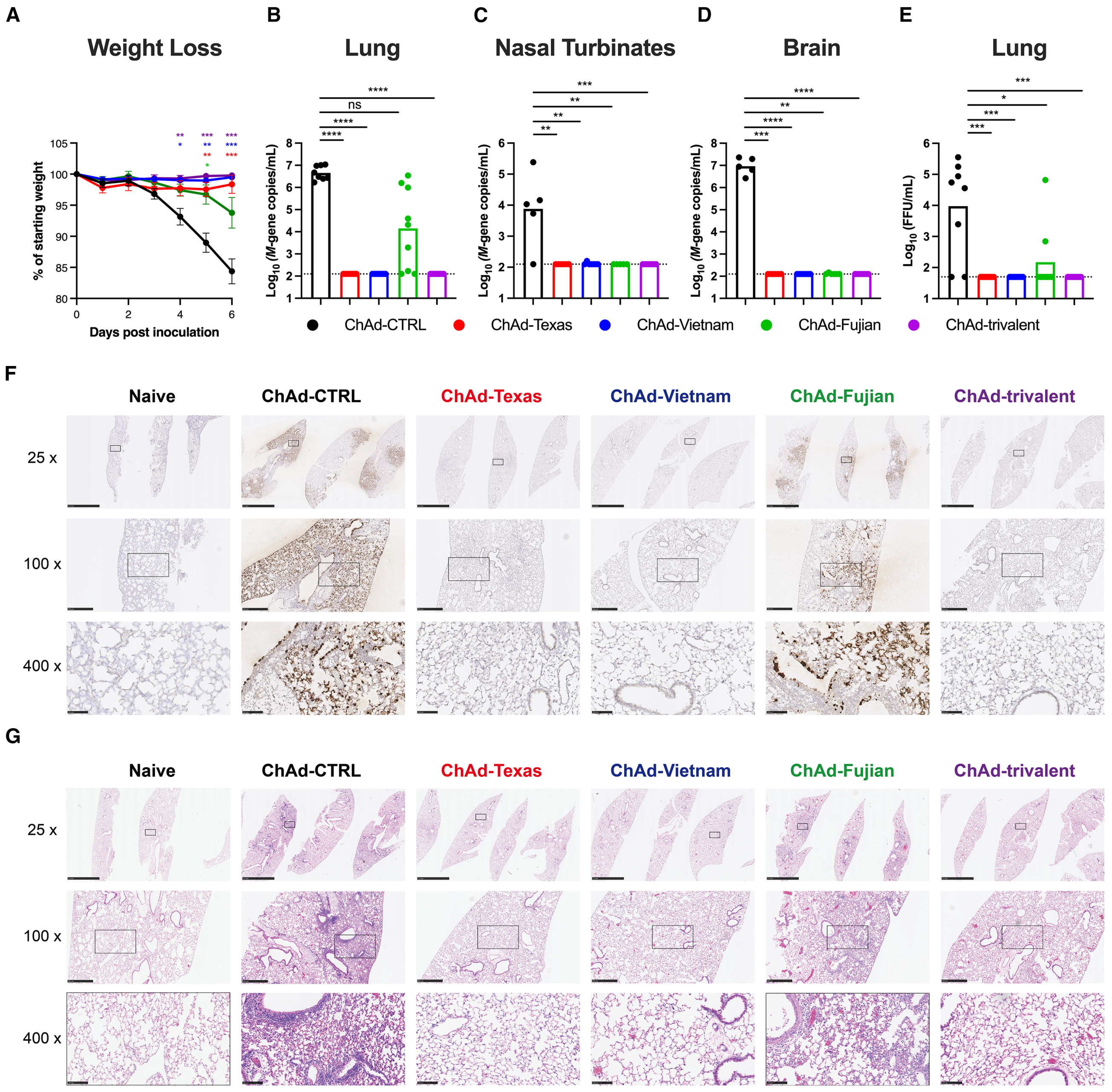

Figure 2. IN-delivered ChAd vaccines protect against challenge by A/Michigan/90/2024

Mice were immunized once via IN route with 10^{10} vp of indicated ChAd vaccines as described in Figure 1. Four to five weeks later, mice were challenged IN with ~20 LD_{50} of A/Michigan/90/2024.

(A) Body weight change.

(B–D) Viral RNA levels were determined at 6 dpi in the lungs (B), nasal turbinates (C), and brains (D).

(E) Infectious virus levels in the lungs.

(F) Viral RNA *in situ* hybridization in lung sections harvested at 6 dpi. Images shown at 25x (scale bars, 2.5 mm), 100x (scale bars, 500 μm) and 400x (scale bars, 100 μm) power magnification.

(G) Hematoxylin and eosin staining of lung sections harvested at 6 dpi. Sections were imaged at 25x (scale bar, 2.5 mm), 100x (scale bars, 500 μm), and 400x (scale bar, 100 μm) magnifications. Each image is representative of a group of 3 mice. Results in this figure are from two experiments (n = 8–10); boxes illustrate geometric mean values, and dotted lines show the limit of detection.

Statistical analysis: (A) two-way ANOVA with Dunnett's post-test; (B–E) Kruskal-Wallis with Dunn's test; ns, not significant, $^{*}p < 0.05$, $^{**}p < 0.01$, $^{***}p < 0.001$, $^{****}p < 0.0001$.

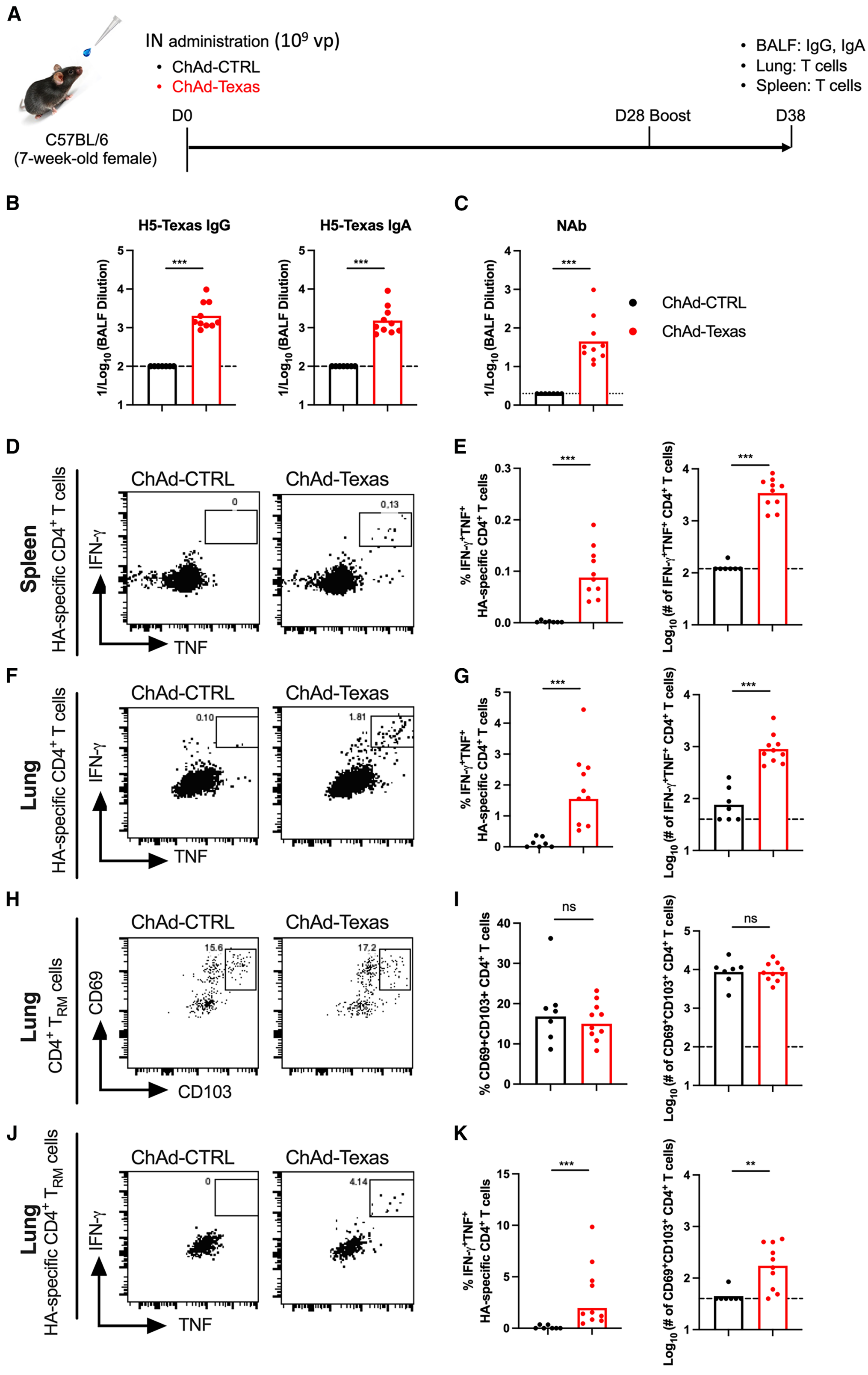

(legend on next page)

high amounts of viral RNA in the lungs (Figure 2B), nasal turbinates (Figure 2C), and brains (Figure 2D), and infectious virus was recovered from the lungs (Figure 2E). Immunization with monovalent ChAd-Texas and ChAd-Vietnam or trivalent ChAd-H5 vaccines provided nearly complete protection against A/Michigan/90/2024 challenge with a >100-fold reduction in viral RNA in the nasal turbinates (Figure 2C), a 10^5-fold reduction in the lungs and brains (Figures 2B–2D), and no detectable infectious virus in the lungs of these vaccinated mice. In contrast, ChAd-Fujian-immunized mice showed a 100- to 1,000-fold reduction in viral RNA in the lungs compared to ChAd-CTRL-immunized mice, with 6 of 9 animals having breakthrough infections (Figures 2B and 2E). Of note, ChAd-Fujian reduced viral RNA levels in the nasal turbinates and brains of H5N1-challenged mice comparably to ChAd-Texas and ChAd-Vietnam (Figures 2C and 2D).

We corroborated the presence of H5N1 viral RNA within lung tissue sections using *in situ* hybridization and a probe targeting A/Michigan/90/2024 viral nucleoprotein (NP) mRNA (Figure 2F). In lung sections of ChAd-CTRL-immunized and H5N1-challenged mice, NP mRNA was widely distributed within the lung interstitium, with intense staining observed in bronchial and alveolar epithelial cells. Substantial NP mRNA also was detected in the lungs of ChAd-Fujian-immunized mice. However, NP mRNA staining was not apparent in the lung sections from ChAd-Texas-, ChAd-Vietnam-, or ChAd-trivalent-immunized mice (Figure 2F).

We assessed the ability of the ChAd vaccines to protect against virus-induced lung damage by analyzing histology at 6 dpi (Figure 2G). Sections from ChAd-CTRL-immunized mice showed signs of severe pneumonia (Figure 2G), whereas mice immunized with monovalent ChAd-Texas, ChAd-Vietnam, or the trivalent ChAd vaccine lacked pathological lesions. However, lungs from ChAd-Fujian-immunized mice showed moderate pneumonia with immune cell infiltration and airspace consolidation (Figure 2G), consistent with the residual viral RNA in the lungs (Figures 2B and 2F). These findings demonstrate that ChAd-vectored vaccines expressing a clade 2.3.4.4b or 2.3.2.1c H5 HA optimally protect against a clade 2.3.4.4b H5N1 virus challenge.

ChAd-Texas induces mucosal antibody and T cell responses

Vaccine-induced mucosal IgA and tissue-resident T cell responses are hypothesized to mitigate severe IAV infection and transmission.[31,42–44] To evaluate mucosal immune responses, mice were immunized twice with ChAd-Texas or ChAd-CTRL via the IN route. Bronchoalveolar lavage fluid (BALF), lung, and spleen were collected at 10 days post-boost for analysis (Figure 3A). BALF from mice immunized with ChAd-Texas had robust IgG and IgA titers and neutralizing activity against H5 HA of A/Texas/37/2024 (Figures 3B and 3C).

Next, we assessed H5 HA-specific $CD4^+$ and $CD8^+$ T cell responses in the lungs and spleens (Figures 3, S3, and S4). Because class I ($CD8^+$) or class II ($CD4^+$) epitopes for clade 2.3.4.4b H5 HA have not yet been reported for C57BL/6 (H-2^b) mice, peptides binding to H2-K^b and H2-D^b (class I, 8- to 11-mers) and H2-I-A^b (class II, 15-mers) were predicted using the H5 HA A/Texas/37/2024 protein sequence (Table S2) and used to stimulate splenocytes and lung cells *ex vivo*. HA-specific $CD4^+$ and $CD8^+$ T cell responses were quantified by intracellular cytokine staining (interferon [IFN]-γ and tumor necrosis factor [TNF]) and flow cytometry (Figures 3D–3K and S4). Although none of the class I peptides induced detectable cytokine production in $CD8^+$ T cells (Figure S4), two class II peptides ($HA_{204–218}$, TNLYKNPITYISVGT, and $HA_{90–104}$, PEWSYIVERANPAND) induced IFN-γ and TNF in $CD4^+$ T cells. Pools of these two peptides were used subsequently to quantify H5 HA-specific $CD4^+$ T cell responses in ChAd-Texas- or ChAd-CTRL-immunized mice. In the spleens (Figures 3D and 3E) and lungs (Figures 3F and 3G) of ChAd-Texas immunized mice, we detected more IFN-γ^+TNF$^+$ $CD4^+$ T cells than in control animals. Although both ChAd-Texas and ChAd-CTRL had comparable levels of total $CD69^+CD103^+$ $CD4^+$ tissue-resident memory T cells (T_{RM}) in the lung (Figures 3H and 3I), only ChAd-Texas increased the frequency and number of H5 HA-specific $CD69^+CD103^+$ $CD4^+$ T_{RM} cells (Figures 3J and 3K).

Nasally delivered ChAd-Texas vaccine confers superior protection against a higher-dose H5N1 challenge

To further assess the protective efficacy of the ChAd-Texas vaccine, we increased the amount of challenge virus. Groups of mice were immunized once IN with 10^9 vp of ChAd-CTRL or ChAd-Texas and then challenged with increasing doses (2,000 or 20,000 FFU) of A/Michigan/90/2024 (Figure S5A). Consistent with results using the 10^{10} vp dose, ChAd-Texas elicited robust serum IgG binding and neutralizing titers against H5-Texas at 4 weeks post-immunization (Figure S5B). Following challenge, mice vaccinated with ChAd-CTRL exhibited substantial weight loss, whereas ChAd-Texas-vaccinated mice were protected from weight loss at all challenge doses (Figure S5C). High viral loads were detected in the lungs and nasal turbinates of ChAd-CTRL-immunized mice (Figures S5D–S5F). In contrast, ChAd-Texas immunization conferred near-complete protection, with virus titers barely detectable, even at the highest challenge dose of $\sim$20,000$\times$ LD_{50} (Figures S5D–S5F).

Figure 3. IN immunization with ChAd-Texas induces mucosal antibody and T cell responses

(A) Experimental design. Mice were immunized IN with 10^9 vp of ChAd-CTRL or ChAd-Texas and boosted 4 weeks later. HA-specific IgG and IgA levels in BALF as well as $CD4^+$ T cell responses in spleen and lung were evaluated on day 10 after the booster immunization.

(B) HA-specific IgG and IgA in BALF.

(C) Neutralizing antibody titers in BALF.

(D–J) Representative flow cytometry plots (D, F, H, and J) and quantification of $CD4^+$ T cell frequencies and total cell numbers (E, G, I, and K) showing IFN-γ^+ and TNF$^+$ $CD4^+$ T cells in the spleen (D and E) and lungs (F and G), total number of $CD69^+CD103^+$ $CD4^+$ tissue-resident memory T (T_{RM}) cells in the lungs (H and I), and HA-specific IFN-γ^+TNF$^+$ $CD69^+CD103^+$ $CD4^+$ T_{RM} cells in the lungs (J and K) following re-stimulation with HA peptides.

Data in this figure are from two independent experiments (n = 7–10). Boxes show mean (for frequencies) or geometric mean (for total cell numbers) values; dotted lines indicate the limit of detection. Statistical analysis was performed using the Mann-Whitney test: $^{**}p < 0.01$, $^{***}p < 0.001$.

CellPress
OPEN ACCESS

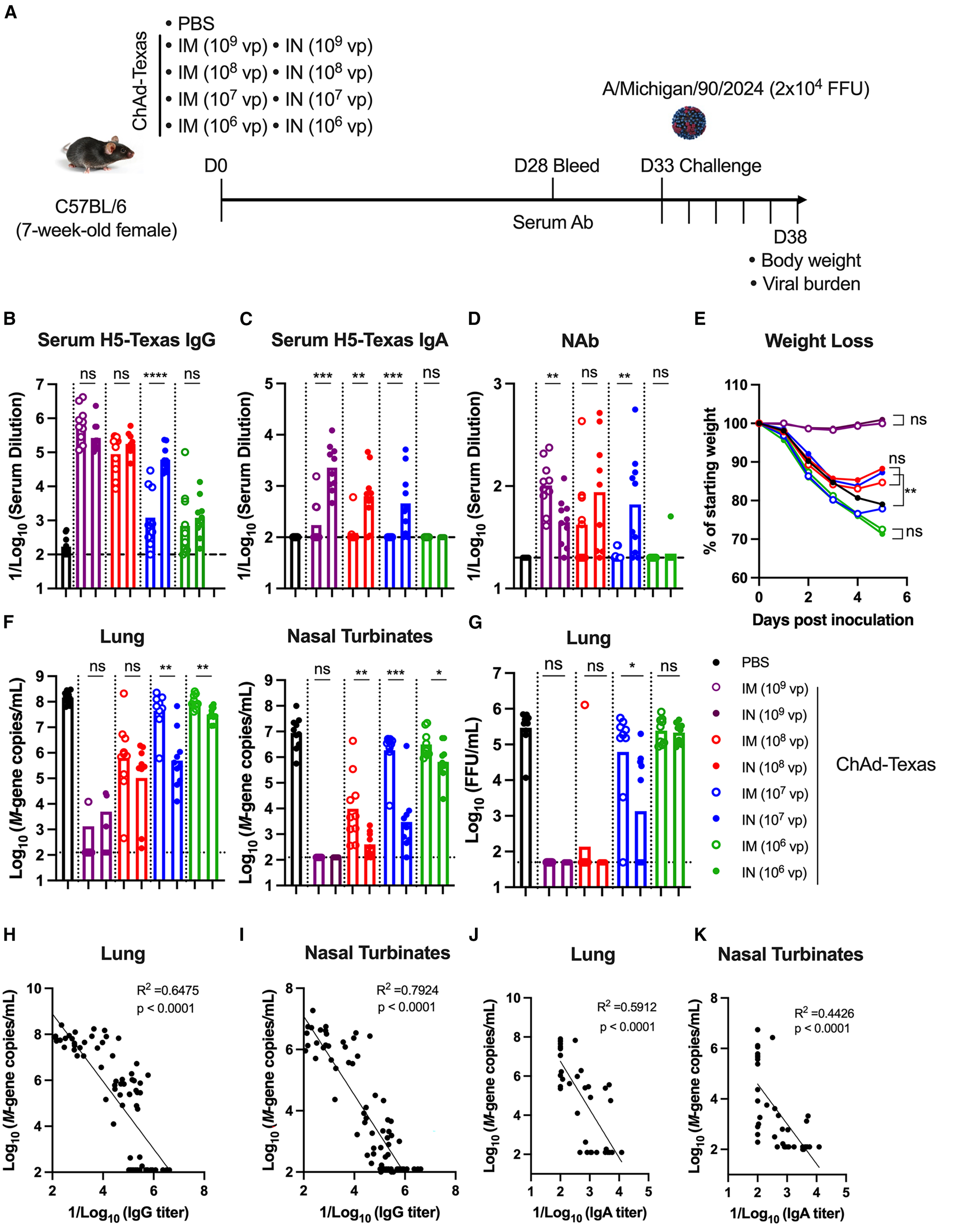

(legend on next page)

To further assess the efficacy of the IN ChAd-Texas vaccine and compare it to an IM delivery route, we reduced the amount of ChAd-Texas vaccine administered. Mice were immunized either IM or IN with 10^9, 10^8, 10^7, or 10^6 dose of ChAd-Texas. A control group received PBS only. Four weeks later, serum antibody responses were measured, and the mice were then challenged with 20,000 FFU of A/Michigan/90/2024 (Figure 4A). Serum H5-specific IgG responses were detectable in both IM- and IN-immunized groups, with titers decreasing in a vaccine dose-dependent manner (Figure 4B). In contrast, IN immunization elicited 10- to 100-fold higher levels of serum H5-specific IgA responses than IM immunization (Figure 4C). Virus-neutralizing antibodies were readily detected in mice immunized IM or IN with the 10^9 dose of ChAd-Texas. Mice that received 10^8 or 10^7 of ChAd-Texas showed lower neutralizing activity in serum with 5/10 and 8/9 mice having detectable neutralization after 10^8 IM and IN and 2/10 and 8/10 after immunization with 10^7 of ChAd-Texas IM and IN, respectively. Neutralizing activity was not detected in mice that received 10^6 ChAd-Texas by either the IM or IN route (Figure 4D).

After challenge, the PBS control mice lost substantial weight. In contrast, mice vaccinated with 10^9 of ChAd-Texas (IM or IN) were fully protected from weight loss (Figure 4E) and had 10,000 to 100,000-fold less viral RNA in the lung and nasal turbinates (Figure 4F). At the 10^8 dose, both IN and IM immunization conferred partial protection, characterized by initial weight loss during the first 4 days post-challenge followed by partial recovery. At this dose, IN-immunized mice sustained lower viral RNA levels in the nasal turbinates (25-fold) and lungs (6-fold) than IM-immunized mice. At the 10^7 dose, the IN-immunized mice exhibited less weight loss and more rapid recovery than IM-immunized mice, and significant reductions in viral RNA levels in the lungs (90-fold, $p < 0.01$) and nasal turbinates (640-fold, $p < 0.001$) were observed (Figure 4F). At the lowest vaccine dose (10^6 of ChAd-Texas), both IN and IM delivery failed to protect from virus infection-induced weight loss. However, viral RNA levels in the lungs (3-fold) and nasal turbinates (5-fold) were reduced after IN compared to IM delivery (Figure 4F). Infectious virus was not detected in the lungs of IN- and IM-immunized mice at the 10^9 dose (Figure 4G). Similarly, at the 10^8 dose, all IN-immunized mice and 9/10 IM-immunized mice lacked infectious virus in their lungs at 5 dpi. At the 10^7 dose, lung viral titers were reduced compared to the control animals and 46-fold lower in IN-immunized mice compared to IM-immunized mice. Reductions in infectious titer were not seen at the 10^6 dose (Figure 4G). Together, these findings demonstrate that IN immunization with ChAd-Texas provides greater protection against viral replication in both the upper and lower respiratory tracts than IM delivery.

We performed correlations between vaccine-induced antibody levels and viral RNA burden in the lung and nasal turbinates at 5 dpi (Figures 4H–4K). Serum H5-specific IgG levels (Figures 4H and 4I) inversely correlated with A/Michigan/90/2024 viral RNA levels in both the lung (Figure 4H) and nasal turbinates (Figure 4I). Serum IgA levels also inversely correlated with viral RNA levels in the lungs and nasal turbinates, although the correlations were less robust (Figures 4J and 4K).

Depletion of CD8$^+$ and CD4$^+$ T cells have limited impact on ChAd-Texas-mediated protection

We next evaluated whether CD8$^+$ or CD4$^+$ T cells contribute to the ChAd vaccine-mediated protection against A/Michigan/90/2024 as shown in Figure S6A. After cell depletion and immunization with 10^8 dose of ChAd-CTRL or ChAd-Texas, sera were collected for antibody analysis and spleens and lungs were collected for CD4$^+$ and CD8$^+$ T cell profiling (Figures S6B–S6E). Comparable levels of serum H5-Texas-specific IgG antibodies were observed among ChAd-Texas-vaccinated mice that received isotype control, anti-CD4, or anti-CD8β antibodies (Figure S6F). Animals treated with anti-CD4 or anti-CD8β antibodies showed nearly complete depletion of CD4$^+$ or CD8$^+$ T cells in the spleen (Figures S6B and S6C) and lung (Figures S6D and S6E), respectively.

On day 36, animals were challenged with 20,000 FFU of A/Michigan/90/2024. ChAd-CTRL immunized mice that received isotype control antibody lost weight and had high levels of viral RNA in the nasal turbinates, lungs, and brain at 5 dpi (Figure S6H). ChAd-Texas vaccinated mice treated with isotype control, anti-CD4, or anti-CD8β antibodies did not lose weight and showed more than 1,000-fold lower levels of viral RNA in the nasal turbinates, lungs, and brains, as well as infectious virus in the lungs (Figures S6G–S6I). Although it did not attain statistical significance, several mice that received anti-CD4 antibodies prior to challenge had higher viral levels in the lungs compared with ChAd-Texas-vaccinated animals that received isotype or CD8β antibodies. Collectively, these findings suggest that antibody responses elicited by ChAd-Texas are the primary mediators of protection against H5N1 virus, with T cell-mediated cellular responses providing less benefit.

Nasally delivered ChAd-Texas vaccine protects against H5N1 infection in Syrian hamsters

To corroborate findings from mice, we evaluated the ChAd-Texas vaccine in Syrian hamsters. Groups of hamsters were

Figure 4. IN-delivered ChAd-Texas vaccine confers superior protection against a highly lethal H5N1 challenge than after IM administration
(A–D) (A) Experimental design. Mice were immunized once either IM or IN with decreasing doses of ChAd-Texas (10^9, 10^8, 10^7, or 10^6 vp) and were subsequently challenged IN with 20,000 FFU of A/Michigan/90/2024 5 weeks later. Serum HA-specific IgG (B) and IgA (C) levels. (D) Serum neutralizing antibody titers.
(E) Body weight changes after virus challenge.
(F) Viral RNA levels at 5 dpi in the lungs and nasal turbinates.
(G) Infectious virus levels in the lungs.
(H–K) Correlation comparing lung (H and J) or nasal turbinates' (I–K) viral RNA levels and serum IgG titers (H and I) or IgA titers (J and K).
Results in this figure are from two experiments (n = 8–10); boxes illustrate geometric mean values, and dotted lines show the limit of detection.
Statistical analysis: (B–D, F, and G) Mann-Whitney test, (E) two-way ANOVA or mixed effect analysis with the Geisser-Greenhouse correction, (H–K) linear regression analysis with p and R^2 values indicated. ns, not significant, *$p < 0.05$, **$p < 0.01$, ***$p < 0.001$, ****$p < 0.0001$.

CellPress
OPEN ACCESS

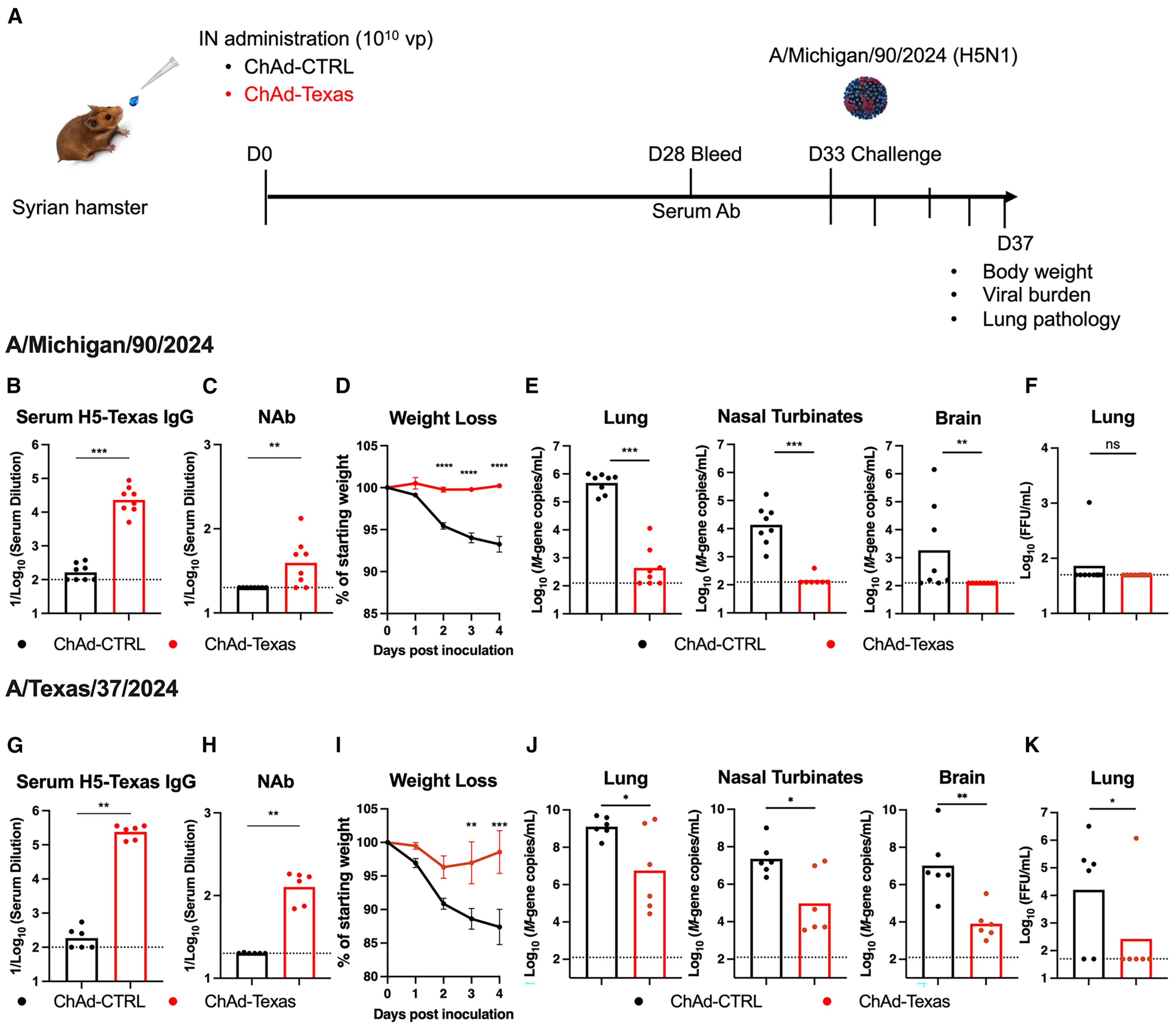

Figure 5. IN-delivered ChAd-Texas vaccine protects Syrian hamsters against A/Michigan/90/2024 and A/Texas/37/2024 challenge

(A) Experimental design.

(B and G) Anti-H5 Texas HA IgG response in serum of hamsters from Charles Rivers Laboratories (B) or Inotiv (G) immunized via IN route with ChAd-CTRL or ChAd-Texas. Serum was collected 28 days after immunization.

(C–F and H–K) (C and H) Serum neutralizing antibody responses against PR8/H5N1. Weight loss (mean ± SEM) after IN challenge with A/Michigan/90/2024 (D) or A/Texas/37/2024 (I). Viral RNA levels in lungs, nasal turbinates, and brain (E and J) as well as infectious virus in the lungs (F and K) after IN challenge with A/Michigan/90/2024 (E and F) or A/Texas/37/2024 (J and K).

Bars indicate geometric mean values, and dotted lines denote the limit of detection of the assays (two experiments, n = 6–8).

Statistical analysis: (B, C, E, F, G, and H) Mann-Whitney test; (J and K) lognormal Welch's unpaired *t* test; (D and I) two-way ANOVA with Bonferroni's post-test. ns, not significant, **p* < 0.05, ***p* < 0.01, ****p* < 0.001, *****p* < 0.0001.

immunized once IN with 10^{10} vp of ChAd-CTRL or ChAd-Texas (Figure 5A). Serum was collected 28 days later, and H5 HA-specific IgG antibodies were measured (Figure 5B). Hamsters immunized with ChAd-Texas, but not ChAd-CTRL, induced serum IgG (Figure 5B) and neutralizing antibody (Figure 5C) responses. Five weeks after immunization, the hamsters were challenged with 10^5 FFU of A/Michigan/90/2024. IN immunization with ChAd-Texas completely protected against weight loss after challenge (Figure 5D) and reduced viral RNA levels in the lung (2,400-fold), nasal turbinates (369-fold), and brain (95-fold) compared to ChAd-CTRL-immunized animals (Figure 5E). For reasons that remain unclear, we did not (1/8 animals) detect infectious virus in the lungs of hamsters at 4 days after A/Michigan/90/2024 challenge (Figure 5F). We also tested the ChAd-Texas vaccine against a highly virulent A/Texas/37/2024 virus containing the PB2-E627K mutation,[13] which is associated with adaptation to

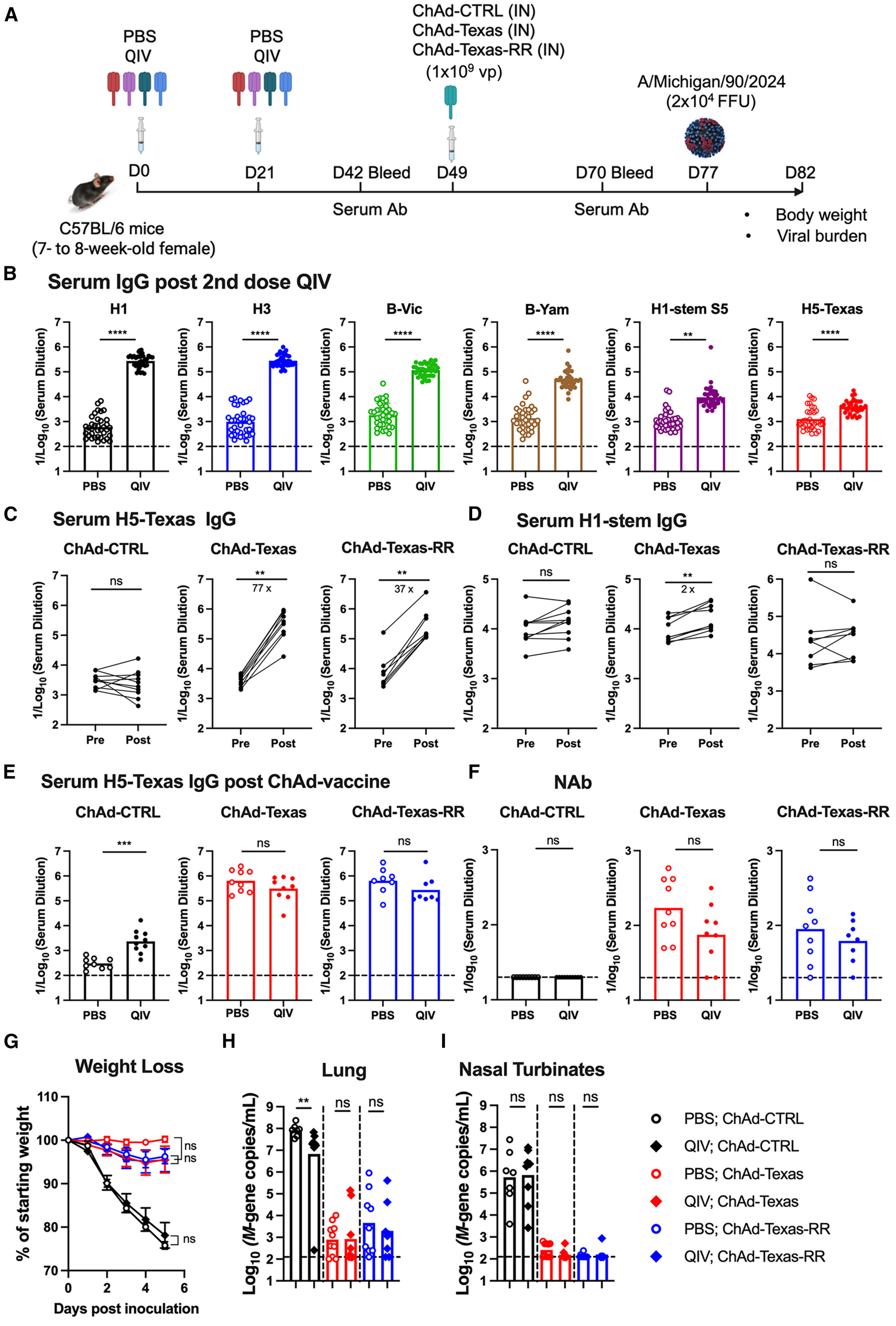

(legend on next page)

mammalian and human hosts (Figures 5G–5K). Again, IN immunization with ChAd-Texas protected against weight loss (Figure 5I) and reduced viral RNA levels (Figure 5J) in the lung (221-fold), nasal turbinates (239-fold), and brain (1,288-fold) and infectious virus in the lung (60-fold) compared to ChAd-CTRL-immunized hamsters (Figure 5K).

IN-delivered ChAd-Texas induces H5-specific immunity and protection in the context of pre-existing immunity

Given that most individuals possess pre-existing immunity to seasonal influenza viruses (H1N1, H3N2, and influenza B viruses), we evaluated whether prior heterosubtypic immunity affects the immunogenicity and efficacy of the ChAd-Texas vaccine. Mice were first immunized IM with two doses (1.5 μg) of Flucelvax quadrivalent seasonal vaccine (2022–2023 formulation, quadrivalent influenza vaccine [QIV]) or PBS as a control, and serum IgG responses to the H1, H3, and influenza B virus HA proteins in the QIV, the H1 stem, and the H5-Texas protein were quantified 3 weeks later. Robust serum IgG responses were detected against HA antigens from H1 (A/Delaware/55/2019), H3 (A/Darwin/11/2021), B-Victoria lineage (B/Singapore/WU4618/2021), and B-Yamagata lineage (B/Singapore/INFTT-16-0610/2016) (Figure 6B). Cross-reactive IgG targeting the conserved stem domain of the H1 HA protein and H5-Texas was also detected (Figure 6B).

To determine whether the QIV vaccine-induced H5-binding antibodies cross-reacted with H1 and H3 HA, QIV-immune sera were absorbed with streptavidin-coated beads loaded with biotinylated HA proteins from H1, H3, or the stem domain of H1 HA. The pre-cleared sera were then evaluated for residual binding to H1, H3, and H5 HA (Figure S7). Depletion of QIV-immune sera with H1 and H3 HA virtually abolished H1, H3, and H5 HA binding (Figure S7A). In comparison, depletion with the H1 HA stem domain protein abrogated H5 HA binding but not H1 or H3 HA binding (Figure S7B), demonstrating that QIV immunization predominantly elicited IgG responses against the HA head domain and that the low levels of H5-binding antibodies induced were cross-reactive and targeted the HA stem region.

Next, these QIV-immunized mice were boosted with ChAd-CTRL, ChAd-Texas, or ChAd-Texas-RR, a derivative vaccine incorporating two arginine substitutions (I45R and T49R) in the stem region of the H5 Texas HA. I45R and T49R substitutions disrupt epitopes recognized by the cross-reactive stem antibody CR9114, while epitopes in the globular head domain remain intact, as indicated by preserved binding to the anti-H5 head antibody AJ-4 (Figure S8), consistent with previous reports.[45,46] Three weeks after boosting, serum was collected, and H1 stem and H5-Texas-specific IgG responses were measured. Boosting with 10^9 vp of ChAd-Texas or ChAd-Texas-RR significantly increased H5-Texas IgG responses with a 77-fold and 37-fold increase in titer, respectively (Figure 6C). This antibody response principally targeted the head domain of H5 HA, as H1 stem-specific IgG levels were only marginally increased in the ChAd-Texas-immunized mice (2-fold, Figure 6D). This increase in stem-specific antibodies, however, was not observed in the ChAd-Texas-RR-boosted animals. Differences in H5-Texas binding (Figure 6E) or neutralizing antibody titers (Figure 6F) were not detected after ChAd-Texas and ChAd-Texas-RR immunization between QIV-primed and the PBS control mice.

We then evaluated the impact of pre-existing immunity induced by QIV on the protective efficacy of ChAd-Texas following A/Michigan/90/2024 challenge. Mice boosted with ChAd-CTRL experienced weight loss after viral challenge (Figure 6G), regardless of prior seasonal influenza virus immune status, indicating that seasonal QIV vaccination alone did not confer robust protection against heterosubtypic H5N1 infection. In contrast, both QIV-primed and unprimed mice boosted with ChAd-Texas or ChAd-Texas-RR were fully protected from weight loss, with no significant differences between groups (Figure 6G). Viral burden analysis showed high levels of viral RNA in the lung (Figure 6H) and nasal turbinates (Figure 6I) of unprimed and ChAd-CTRL-boosted mice following H5N1 virus challenge. QIV priming followed by a boost with ChAd-CTRL resulted in a ∼10-fold lower viral RNA load in the lung (Figure 6H) but not in the nasal turbinates (Figure 6I) compared to unprimed counterparts. In contrast, viral RNA levels in the lung and nasal turbinates of ChAd-Texas- or ChAd-Texas-RR-boosted mice were reduced 50,000- and 4,000-fold, respectively. Statistical differences in virus titer in the lung or nasal turbinates were not detected between the unprimed and QIV-primed mice after ChAd-Texas or ChAd-Texas-RR vaccination (Figures 6H and 6I).

To extend these findings, we primed with an antigenically divergent H1 HA protein (A/New Caledonia/20/1999) followed by a boost with QIV 3 weeks later (Figure S9A). This design

Figure 6. ChAd-Texas induces H5-specific immunity and protection against H5N1 in the context of pre-existing immunity to seasonal influenza vaccine

(A) Experimental design. Mice were immunized IM with either PBS or 1.5 μg of the Flucelvax QIV twice at a 3-week interval. Four weeks after the final QIV dose, mice received an IN immunization of 10^9 vp of ChAd-CTRL, ChAd-Texas, or ChAd-Texas-RR vaccine. Serum samples were collected 3 weeks after QIV vaccine and 3 weeks after the ChAd vaccine boost to evaluate antibody responses.

(B) Serum IgG responses against different HA proteins 21 days after QIV immunization.

(C) Paired analysis of serum IgG titers pre- and post-boost with indicated ChAd vaccine against H5-Texas.

(D) Paired analysis of serum IgG titers pre- and post-boost with indicated ChAd vaccine against H1 HA-stem protein.

(E) Serum H5-HA binding IgG titers after ChAd vaccine boost in mice previously primed with PBS or QIV.

(F) Serum neutralizing antibody titers after ChAd vaccine boost in mice primed with PBS or QIV.

(G) Weight loss after IN challenge with A/Michigan/90/2024.

(H and I) Viral RNA levels determined at 5 dpi in lungs (H) and nasal turbinates (I).

n = 7–10 mice per group, two independent experiments. Column heights indicate GMT, and dotted lines show the limit of detection. GMTs or fold-changes are indicated above corresponding graphs.

Statistical analyses: (B, E, F, H, and I) Mann-Whitney test, (C and D) Wilcoxon matched signed-rank test, (G) two-way ANOVA with Tukey's post-test. ns, not significant; $^{**}p < 0.01$, $^{***}p < 0.001$, $^{****}p < 0.0001$.

was intended to boost HA stem-specific and H5 HA cross-reactive immune responses prior to ChAd-Texas immunization. Consistent with QIV/QIV immunization results, we observed high IgG titers against H1, H3, B-Victoria, and B-Yamagata HA proteins and high titers against H1 stem-reactive IgG antibodies with an 8-fold increase compared to the PBS control group (Figure S9B). We also observed a ~14-fold increase in H5 cross-reactive IgG titer in the rH1+QIV immunized mice (Figure S9B). Boosting with a 10^9 dose of ChAd-Texas or ChAd-Texas-RR increased the H5-Texas-specific response by 9.5- and 5.4-fold, respectively (Figure S9D), and induced neutralizing antibodies in serum (Figure S9E). Like the QIV/QIV studies, boosting of the H1 stem response only occurred with ChAd-Texas (~2-fold) but not ChAd-Texas-RR or ChAd-CTRL (Figure S9C). Following H5N1 challenge with A/Michigan/90/2024, rH1+QIV-primed mice boosted with ChAd-CTRL had similar weight loss (Figure S9F) and viral RNA levels in the lung, nasal turbinates, and brain (Figure S9G) as naive mice boosted with ChAd-CTRL. In contrast, ChAd-Texas and ChAd-Texas-RR boosting of rH1+QIV-primed mice resulted in less weight loss and reduced viral loads in the lung (5,000- to 10,000-fold), nasal turbinates (6,000-fold), and brain (160-fold) compared to ChAd-CTRL-boosted mice (Figures S9F and S9G). Differences in efficacy were not observed between ChAd-Texas and ChAd-Texas-RR. Altogether, our data demonstrate that the IN ChAd-Texas vaccine is effective in mice in the context of pre-existing systemic immunity to antigens from seasonal influenza viruses.

DISCUSSION

We developed a series of ChAd-vectored vaccines encoding the HA protein from distinct H5N1 clades and evaluated their immunogenicity and protective efficacy against currently circulating H5N1 strains in mice and Syrian hamsters. IN delivery of the ChAd-vectored H5 HA vaccines elicited neutralizing serum antibodies, mucosal IgG and IgA antibody responses, and T cell immune responses and conferred greater protection against a high-dose H5N1 virus challenge than IM vaccination. Prior seasonal influenza virus immunity did not impair antibody responses or protection conferred by IN ChAd-Texas vaccine. Finally, in hamsters, a single IN dose of ChAd-Texas prevented weight loss and reduced upper and lower respiratory tract infection after H5N1 A/Michigan/90/2024 or A/Texas/37/2024 challenge.

Recent studies in cynomolgus macaques showed that intratracheal boosting after IM priming with a homologous rhesus adenovirus serotype 52 (RhAd52)-vectored influenza vaccine (H5 or H1) elicited robust mucosal antibody and T cell responses and conferred superior protection than IM boosting.[47,48] Our work uses a mucosal ChAd-vectored H5 vaccine to evaluate protection and address the impact of pre-existing immunity induced by heterologous seasonal QIV vaccination on vaccine-induced immunity. We demonstrate that mucosal delivery of a ChAd-vectored H5 vaccine can overcome pre-existing seasonal influenza immunity while maintaining strong protection. Together, these two studies support further development of mucosally delivered Ad-vectored H5 vaccines as a platform for HPAI H5N1 preparedness.

The H5 HA of HPAI continues to evolve genetically and antigenically, and it is not known which clade will cause an outbreak or potential pandemic. While ChAd-Texas and ChAd-Vietnam vaccine induced high levels of IgG and IgA antibodies recognizing all three clades (clade 2.3.4.4b, 2.3.2.1c, and 2.4.4.4h) and were associated with near-complete protection against A/Michigan/90/2024 virus challenge, ChAd-Fujian, a clade 2.3.4.4h H5 vaccine, induced lower levels of H5-Texas or H5-Vietnam specific antibodies and conferred less protection against a clade 2.3.4.4b challenge. Although the basis for this difference in antibody response remains unknown, analysis of amino acid sequences of HA proteins (Figures S1B and S1C) showed that H5-Texas is more similar to H5-Fujian than H5-Vietnam. However, the percent sequence identity does not always predict antigenic similarity, the latter of which may be a better predictor of cross-protective antibody responses.[49] Indeed, even a single amino acid deletion at position 131 in the HA protein (131Δ, H3 numbering, or 126 in H5) in the 2.3.4.4h Fujian strain, which has been found disproportionally in human-infecting viruses,[50] can skew a cross-reactive to a more subclade-specific response.

The precise mechanism by which the ChAd-vectored H5 vaccine protects against high-dose challenge by HPAI H5N1 virus remains to be elucidated. Our correlation analysis revealed that serum H5-specific IgG levels, and to a lesser extent IgA levels, inversely correlated with A/Michigan/90/2024 viral RNA levels in both the lung and nasal turbinate. Since the IN-delivered ChAd-H5 vaccine induces high levels of mucosal IgG and IgA antibodies capable of neutralizing H5 virus, we expected that mucosal antibody responses might correlate better with protection than serum antibodies. The mechanism of protection against HPAI H5N1 contrasts with that observed for ChAd-SARS-CoV-2-S, where cross-protective immunity against viral variants was associated with robust $CD8^+$ T cell responses in the lungs.[51] However, we did not detect a $CD8^+$ T cell response in H5 HA using 16 different predicted epitopes, and $CD8^+$ T cell depletion had minimal impact on ChAd-Texas-mediated protection. These results indicate that antibody responses elicited by ChAd-Texas likely are the primary mediators of vaccine-mediated protection.

Our virus challenge data demonstrate that a single IN dose of the ChAd-Texas vaccine protects against H5N1 A/Michigan/90/2024, even under a stringent challenge of more than 10,000 x LD_{50}. Whereas nearly complete protection was observed against viral replication and clinical disease at the highest vaccine dose tested, robust protection was still evident even at a 100-fold lower dose of ChAd-Texas vaccine. Our studies revealed that (1) IN delivery of the ChAd-Texas vaccine is more immunogenic at lower doses of the vaccine than IM administration; (2) unlike IM delivery, IN delivery of ChAd-Texas induces robust levels of IgA in serum and mucosa; and (3) serum IgG and IgA responses correlate with protection from infection. Future studies can use the lower vaccine doses to identify the correlates of immune protection against homologous and heterologous challenge. These data are consistent with previous reports showing that IN vaccination provides better protection than IM immunization against respiratory virus infections, including SARS-CoV-2 and seasonal influenza.[30,31]

Given that most individuals possess pre-existing immunity to seasonal influenza viruses (H1N1, H3N2, and influenza B virus), we evaluated the immunogenicity and efficacy of the ChAd-Texas vaccine in pre-immune mice. Significant differences in H5 HA-specific or neutralizing antibody titer were not observed between pre-immune and unprimed mice. As such, IN delivery of the ChAd-Texas vaccine protected both groups of mice against high-dose H5N1 challenge. Boosting with ChAd-Texas, but not ChAd-Texas-RR, increased H1-stem-specific antibody responses, suggesting that some back-boosting occurs, and masking or abrogating a dominant HA-stem epitope can negate at least some of this effect. Our findings contrast with reports in humans,[39] where individuals immunized IM once with an inactivated H5N1 vaccine derived from A/Vietnam/1203/2004 or A/Indonesia/5/2005 produced high amounts of anti-HA stem antibodies and not the more effective anti-H5 head-specific antibodies. Multiple factors could contribute to the discrepancies between studies. (1) Vaccine platform: the human studies used inactivated H5N1 vaccines, whereas we used a ChAd-vectored vaccine. (2) Vaccine antigen: while unlikely to play a major role, the human studies used a different H5 HA (clade 1 and 2.1) compared to the clade 2.3.4.4b H5 HA used in this study. (3) Vaccine dose: the 10^9 dose of ChAd-Texas may overcome immune imprinting by delivering sufficient H5 antigen to surmount memory B cell seniority and activate naive B cells directed against the H5 HA head domain. (4) Route of immunization: different routes of immunization engage distinct anatomical sites for antigen presentation and B cell activation. Repetitive IM immunization may skew toward reactivating pre-existing cross-reactive memory B cells instead of *de novo* H5-specific antibody responses. (5) Immune history: in our study, mice were primed with either QIV/QIV or rH1+QIV vaccines. Immune history in humans is more complex, as individuals are exposed to multiple influenza infections and vaccinations throughout their lifetime.

Previously, it was shown that multiple immunizations with a seasonal trivalent inactivated vaccine can confer partial protection in BALB/c mice against H5N1 strains, including A/Hong Kong/156/1997, A/Vietnam/1194/2004, and A/Indonesia/6/2005,[32,52] even in the absence of detectable H5N1-neutralizing antibodies. Similarly, infection with influenza A (H1N1) pdm09 (pH1N1) virus protected ferrets against cow/Texas/24 H5N1 without detectable neutralizing antibodies.[53] In our study, we observed a 10-fold reduction in virus load in the lungs, but no reduction in weight loss or virus titer in the nasal turbinates in the QIV/QIV-primed mice following challenge with H5N1 virus. This difference is likely caused by the higher challenge dose used in our studies ($\sim$10,000 x LD_{50}). Whether this partial protection is mediated by non-neutralizing cross-reactive antibodies to HA or NA proteins or by tissue-resident memory T cells in the respiratory tract remains to be determined.

Limitations of the study

(1) The effectiveness of the ChAd-Texas vaccine against clade 2.3.2.1c and 2.3.4.4h H5N1 viruses needs to be evaluated. (2) Longitudinal studies are needed to assess the durability of mucosal and systemic immunity as well as protective efficacy. (3) Due to the invasive and terminal nature of the BAL procedure, we could not correlate pre-existing mucosal IgA and lung T cell responses with protection in our animal models. (4) Our study was designed to evaluate the immunogenicity and efficacy of IN-delivered ChAd-vectored H5 vaccines against H5N1 infection in both naive and antigen-experienced animals. We did not conduct a systematic comparison of IN versus IM delivery of the ChAd-H5 vaccine under pre-existing QIV exposure conditions. (5) We did not assess the vaccine efficacy on H5N1 virus transmission. We conducted a direct-contact transmission study to assess the efficacy of the ChAd-H5 vaccine against virus transmission in hamsters using the H5N1 A/Texas/37/2024 strain but were unable to consistently detect H5N1 virus transmission in the control group. (6) As our study focused on the protection of Ad-vectored H5 mucosal vaccines directed against infection by contemporary H5N1 strains in naive and antigen-experienced animals in preclinical models, their safety and efficacy in non-human primates and humans await evaluation. Some Ad-vectored COVID-19 vaccines (ChAdOx1 nCoV-19 and Ad26.COV2) delivered IM have been associated with rare adverse events of thrombosis and thrombocytopenia[54]; these issues have not yet been reported with the mucosally administered ChAd-SARS-CoV-2-S (iNCOVACC) and Ad5-nCoV-S (Convidecia Air) vaccines used in India and China, respectively.[55–57] The impact of vaccine backbones and genetic modifications as well as routes of administration on the safety profile warrants further investigation.

Collectively, our study provides evidence that an IN-delivered ChAd-Texas vaccine elicits robust systemic and mucosal H5-specific neutralizing antibody and T cell responses, conferring strong protection against contemporary HPAI H5N1 infection, even in the context of pre-existing immunity to seasonal influenza viruses. Although human clinical trials are warranted, a strain-updated formulation of this viral-vectored IN vaccine holds promise as a platform for HPAI H5N1 pandemic preparedness.

RESOURCE AVAILABILITY

Lead contact

Further information and requests for resources and reagents should be directed to the lead contact, Adrianus C.M. Boon (jboon@wustl.edu).

Materials availability

All requests for resources and reagents should be directed to the lead contact author. This includes viruses, vaccines, proteins, peptides, and primer-probe sets. All reagents will be made available on request after completion of a materials transfer agreement (MTA).

Data and code availability

- All data supporting the findings of this study are available within the paper, supplemental information or extended data files, or online (https://doi.org/10.17632/7cjrxg6hjd).
- No original code is associated with this study.
- Any additional information related to the study also is available from the corresponding author upon reasonable request.

ACKNOWLEDGMENTS

This study was supported by the Cooperative Center for Human Immunology (U19AI181103 to A.C.M.B. and M.S.D.) and the Center for Research on Structural Biology of Infectious Diseases (75N93022C00035 to A.C.M.B. and

E.M.S.). We thank Igor P. Dmitriev for his assistance in amplifying the Chimpanzee adenovirus vaccines.

AUTHOR CONTRIBUTIONS

B.Y., K.P., H.H.H., and T.L.D. performed *in vivo* experiments, antibody binding studies, virus titrations, and pathology. L.-C.H. generated the IAV. K.S. and T.P. performed virus neutralization studies. A.S. generated the HA proteins. E.-M.S., M.S.D., and A.C.M.B. designed the studies. B.Y., M.S.D., and A.C.M.B. analyzed the data and performed the statistical analysis. J.B.C., E.A.K., and D.T.C. generated the ChAd vaccines. B.P. performed T cell epitope predictions. B.Y. and P.D. performed T cell analysis. A.C.M.B., M.S.D., D.T.C., and E.M.S. supervised experiments and acquired funding. B.Y., M.S.D., and A.C.M.B. wrote the first draft of the manuscript, and all authors edited the final version. All authors approved the final draft and take full responsibility for its content.

DECLARATION OF INTERESTS

The Boon laboratory has received funding from Novavax Inc for the development of an influenza virus vaccine and unrelated funding support from AbbVie Inc. M.S.D. is a consultant for or on the Scientific Advisory Board of Inbios, IntegerBio, Akagera Medicines, GlaxoSmithKline, Merck, and Moderna. The Diamond laboratory has received unrelated funding support in sponsored research agreements from Moderna.

STAR★METHODS

Detailed methods are provided in the online version of this paper and include the following:

- KEY RESOURCES TABLE
- EXPERIMENTAL MODEL AND SUBJECT DETAILS
  - Cells
  - Viruses
  - Animals
- METHOD DETAILS
  - Construction of chimpanzee adenoviral vectors
  - Generation of recombinant influenza virus HA proteins
  - HA multiplex binding assays
  - Antibody depletion assay
  - Virus neutralization assay
  - Mouse immunization and challenge experiments
  - Hamster experiments
  - Measurement of viral burden
  - Lung histology and viral RNA *in situ* hybridization
  - H5 HA CD4 and CD8 T cell epitopes prediction
  - T cell analysis
  - T cells depletion experiments
- QUANTIFICATION AND STATISTICAL ANALYSIS

SUPPLEMENTAL INFORMATION

Supplemental information can be found online at https://doi.org/10.1016/j.xcrm.2025.102582.

Received: September 18, 2025
Revised: November 26, 2025
Accepted: December 30, 2025
Published: January 30, 2026

REFERENCES

1. Chan, P.K.S. (2002). Outbreak of avian influenza A(H5N1) virus infection in Hong Kong in 1997. Clin. Infect. Dis. *34*, S58–S64. https://doi.org/10.1086/338820.

2. Subbarao, K., Klimov, A., Katz, J., Regnery, H., Lim, W., Hall, H., Perdue, M., Swayne, D., Bender, C., Huang, J., et al. (1998). Characterization of an avian influenza A (H5N1) virus isolated from a child with a fatal respiratory illness. Science *279*, 393–396. https://doi.org/10.1126/science.279.5349.393.

3. Claas, E.C., Osterhaus, A.D., van Beek, R., De Jong, J.C., Rimmelzwaan, G.F., Senne, D.A., Krauss, S., Shortridge, K.F., and Webster, R.G. (1998). Human influenza A H5N1 virus related to a highly pathogenic avian influenza virus. Lancet *351*, 472–477. https://doi.org/10.1016/s0140-6736(97)11212-0.

4. Claas, E.C., de Jong, J.C., van Beek, R., Rimmelzwaan, G.F., and Osterhaus, A.D. (1998). Human influenza virus A/HongKong/156/97 (H5N1) infection. Vaccine *16*, 977–978.

5. Webster, R.G., and Govorkova, E.A. (2006). H5N1 influenza–continuing evolution and spread. N. Engl. J. Med. *355*, 2174–2177. https://doi.org/10.1056/NEJMp068205.

6. Wille, M., and Barr, I.G. (2022). Resurgence of avian influenza virus. Science *376*, 459–460. https://doi.org/10.1126/science.abo1232.

7. Smith, G.J.D., and Donis, R.O.; World Health Organization/World Organisation for Animal Health/Food and Agriculture Organization WHO/OIE/FAO H5 Evolution Working Group & Agriculture Organization (2015). H. E. W. G. Nomenclature updates resulting from the evolution of avian influenza A(H5) virus clades 2.1.3.2a, 2.2.1, and 2.3.4 during 2013-2014. Influenza Other Respir. Viruses *9*, 271–276. https://doi.org/10.1111/irv.12324.

8. Charostad, J., Rezaei Zadeh Rukerd, M., Mahmoudvand, S., Bashash, D., Hashemi, S.M.A., Nakhaie, M., and Zandi, K. (2023). A comprehensive review of highly pathogenic avian influenza (HPAI) H5N1: An imminent threat at doorstep. Travel Med. Infect. Dis. *55*, 102638. https://doi.org/10.1016/j.tmaid.2023.102638.

9. Kandeil, A., Patton, C., Jones, J.C., Jeevan, T., Harrington, W.N., Trifkovic, S., Seiler, J.P., Fabrizio, T., Woodard, K., Turner, J.C., et al. (2023). Rapid evolution of A(H5N1) influenza viruses after intercontinental spread to North America. Nat. Commun. *14*, 3082. https://doi.org/10.1038/s41467-023-38415-7.

10. Pulit-Penaloza, J.A., Brock, N., Pappas, C., Sun, X., Belser, J.A., Zeng, H., Tumpey, T.M., and Maines, T.R. (2020). Characterization of highly pathogenic avian influenza H5Nx viruses in the ferret model. Sci. Rep. *10*, 12700. https://doi.org/10.1038/s41598-020-69535-5.

11. Youk, S., Torchetti, M.K., Lantz, K., Lenoch, J.B., Killian, M.L., Leyson, C., Bevins, S.N., Dilione, K., Ip, H.S., Stallknecht, D.E., et al. (2023). H5N1 highly pathogenic avian influenza clade 2.3.4.4b in wild and domestic birds: Introductions into the United States and reassortments, December 2021-April 2022. Virology *587*, 109860. https://doi.org/10.1016/j.virol.2023.109860.

12. Webby, R.J., and Uyeki, T.M. (2024). An Update on Highly Pathogenic Avian Influenza A(H5N1) Virus, Clade 2.3.4.4b. J. Infect. Dis. *230*, 533–542. https://doi.org/10.1093/infdis/jiae379.

13. Mostafa, A., Naguib, M.M., Nogales, A., Barre, R.S., Stewart, J.P., García-Sastre, A., and Martinez-Sobrido, L. (2024). Avian influenza A (H5N1) virus in dairy cattle: origin, evolution, and cross-species transmission. mBio *15*, e0254224. https://doi.org/10.1128/mbio.02542-24.

14. Peacock, T.P., Moncla, L., Dudas, G., VanInsberghe, D., Sukhova, K., Lloyd-Smith, J.O., Worobey, M., Lowen, A.C., and Nelson, M.I. (2025). The global H5N1 influenza panzootic in mammals. Nature *637*, 304–313. https://doi.org/10.1038/s41586-024-08054-z.

15. Xie, R., Edwards, K.M., Wille, M., Wei, X., Wong, S.S., Zanin, M., El-Shesheny, R., Ducatez, M., Poon, L.L.M., Kayali, G., et al. (2023). The episodic resurgence of highly pathogenic avian influenza H5 virus. Nature *622*, 810–817. https://doi.org/10.1038/s41586-023-06631-2.

16. Fusaro, A., Zecchin, B., Giussani, E., Palumbo, E., Agüero-García, M., Bachofen, C., Bálint, Á., Banihashem, F., Banyard, A.C., Beerens, N., et al. (2024). High pathogenic avian influenza A(H5) viruses of clade

CellPress
OPEN ACCESS

2.3.4.4b in Europe-Why trends of virus evolution are more difficult to predict. Virus Evol. *10*, veae027. https://doi.org/10.1093/ve/veae027.

17. Caliendo, V., Lewis, N.S., Pohlmann, A., Baillie, S.R., Banyard, A.C., Beer, M., Brown, I.H., Fouchier, R.A.M., Hansen, R.D.E., Lameris, T.K., et al. (2022). Transatlantic spread of highly pathogenic avian influenza H5N1 by wild birds from Europe to North America in 2021. Sci. Rep. *12*, 11729. https://doi.org/10.1038/s41598-022-13447-z.
18. Burrough, E.R., Magstadt, D.R., Petersen, B., Timmermans, S.J., Gauger, P.C., Zhang, J., Siepker, C., Mainenti, M., Li, G., Thompson, A.C., et al. (2024). Highly Pathogenic Avian Influenza A(H5N1) Clade 2.3.4.4b Virus Infection in Domestic Dairy Cattle and Cats, United States, 2024. Emerg. Infect. Dis. *30*, 1335–1343. https://doi.org/10.3201/eid3007.240508.
19. Caserta, L.C., Frye, E.A., Butt, S.L., Laverack, M., Nooruzzaman, M., Covaleda, L.M., Thompson, A.C., Koscielny, M.P., Cronk, B., Johnson, A., et al. (2024). Spillover of highly pathogenic avian influenza H5N1 virus to dairy cattle. Nature *634*, 669–676. https://doi.org/10.1038/s41586-024-07849-4.
20. Neumann, G., and Kawaoka, Y. (2024). Highly pathogenic H5N1 avian influenza virus outbreak in cattle: the knowns and unknowns. Nat. Rev. Microbiol. *22*, 525–526. https://doi.org/10.1038/s41579-024-01087-1.
21. CDC: Centers for Disease Control and Prevention. H5 Bird Flu: Current Situation, <https://www.cdc.gov/bird-flu/situation-summary/>(2025).
22. US Food and Drug Administration. Influenza virus vaccine, H5N1 (for national stockpile) <https://www.fda.gov/vaccines-blood-biologics/vaccines/influenza-virus-vaccine-h5n1-national-stockpile>(given here is invalid or inaccessible. Please verify the address and correct if required.>2018).
23. US Food and Drug Administration (2019). Influenza A (H5N1) Virus Monovalent Vaccine, Adjuvanted. https://www.fda.gov/vaccines-blood-biologics/vaccines/influenza-h5n1-virus-monovalent-vaccine-adjuvanted.
24. US Food and Drug Administration (2024). AUDENZ, Influenza A (H5N1) Monovalent Vaccine, Adjuvanted. https://www.fda.gov/vaccines-blood-biologics/audenz.
25. Khurana, S., King, L.R., Manischewitz, J., Posadas, O., Mishra, A.K., Liu, D., Beigel, J.H., Rappuoli, R., Tsang, J.S., and Golding, H. (2024). Licensed H5N1 vaccines generate cross-neutralizing antibodies against highly pathogenic H5N1 clade 2.3.4.4b influenza virus. Nat. Med. *30*, 2771–2776. https://doi.org/10.1038/s41591-024-03189-y.
26. Furey, C., Scher, G., Ye, N., Kercher, L., DeBeauchamp, J., Crumpton, J.C., Jeevan, T., Patton, C., Franks, J., Rubrum, A., et al. (2024). Development of a nucleoside-modified mRNA vaccine against clade 2.3.4.4b H5 highly pathogenic avian influenza virus. Nat. Commun. *15*, 4350. https://doi.org/10.1038/s41467-024-48555-z.
27. Hatta, M., Hatta, Y., Choi, A., Hossain, J., Feng, C., Keller, M.W., Ritter, J.M., Huang, Y., Fang, E., Pusch, E.A., et al. (2024). An influenza mRNA vaccine protects ferrets from lethal infection with highly pathogenic avian influenza A(H5N1) virus. Sci. Transl. Med. *16*, eads1273. https://doi.org/10.1126/scitranslmed.ads1273.
28. Hawman, D.W., Tipih, T., Hodge, E., Stone, E.T., Warner, N., McCarthy, N., Granger, B., Meade-White, K., Leventhal, S., Hatzakis, K., et al. (2025). Clade 2.3.4.4b but not historical clade 1 HA replicating RNA vaccine protects against bovine H5N1 challenge in mice. Nat. Commun. *16*, 655. https://doi.org/10.1038/s41467-024-55546-7.
29. Darling, T.L., Harastani, H.H., Joshi, A., Bricker, T.L., Soudani, N., Seehra, K., Hassan, A.O., Diamond, M.S., and Boon, A.C.M. (2024). Mucosal immunization with ChAd-SARS-CoV-2-S prevents sequential transmission of SARS-CoV-2 to unvaccinated hamsters. Sci. Adv. *10*, eadp1290. https://doi.org/10.1126/sciadv.adp1290.
30. Hassan, A.O., Kafai, N.M., Dmitriev, I.P., Fox, J.M., Smith, B.K., Harvey, I.B., Chen, R.E., Winkler, E.S., Wessel, A.W., Case, J.B., et al. (2020). A Single-Dose Intranasal ChAd Vaccine Protects Upper and Lower Respiratory Tracts against SARS-CoV-2. Cell *183*, 169–184.e13. https://doi.org/10.1016/j.cell.2020.08.026.
31. Wang, L., Chan, C.N., Chaudhari, J., Hope, D., Espinosa Perez, A.A., Lasrado, N., Li, Z., Waller-Pulido, A., Cabrera-Barragan, D.N., Verrette, B., et al. (2025). Mucosal boosting increases protective efficacy of an influenza vaccine in mice. iScience *28*, 112721. https://doi.org/10.1016/j.isci.2025.112721.
32. Ichinohe, T., Tamura, S.I., Kawaguchi, A., Ninomiya, A., Imai, M., Itamura, S., Odagiri, T., Tashiro, M., Takahashi, H., Sawa, H., et al. (2007). Cross-protection against H5N1 influenza virus infection is afforded by intranasal inoculation with seasonal trivalent inactivated influenza vaccine. J. Infect. Dis. *196*, 1313–1320. https://doi.org/10.1086/521304.
33. Holmes, E.C., Krammer, F., and Goodrum, F.D. (2024). Virology-The next fifty years. Cell *187*, 5128–5145. https://doi.org/10.1016/j.cell.2024.07.025.
34. Sheikh-Mohamed, S., Sanders, E.C., Gommerman, J.L., and Tal, M.C. (2022). Guardians of the oral and nasopharyngeal galaxy: IgA and protection against SARS-CoV-2 infection. Immunol. Rev. *309*, 75–85. https://doi.org/10.1111/imr.13118.
35. Abreu, R.B., Kirchenbaum, G.A., Clutter, E.F., Sautto, G.A., and Ross, T.M. (2020). Preexisting subtype immunodominance shapes memory B cell recall response to influenza vaccination. JCI Insight *5*, e132155. https://doi.org/10.1172/jci.insight.132155.
36. Dugan, H.L., Guthmiller, J.J., Arevalo, P., Huang, M., Chen, Y.Q., Neu, K.E., Henry, C., Zheng, N.Y., Lan, L.Y.L., Tepora, M.E., et al. (2020). Preexisting immunity shapes distinct antibody landscapes after influenza virus infection and vaccination in humans. Sci. Transl. Med. *12*, eabd3601. https://doi.org/10.1126/scitranslmed.abd3601.
37. Gostic, K.M., Ambrose, M., Worobey, M., and Lloyd-Smith, J.O. (2016). Potent protection against H5N1 and H7N9 influenza via childhood hemagglutinin imprinting. Science *354*, 722–726. https://doi.org/10.1126/science.aag1322.
38. Nachbagauer, R., Choi, A., Hirsh, A., Margine, I., Iida, S., Barrera, A., Ferres, M., Albrecht, R.A., García-Sastre, A., Bouvier, N.M., et al. (2017). Defining the antibody cross-reactome directed against the influenza virus surface glycoproteins. Nat. Immunol. *18*, 464–473. https://doi.org/10.1038/ni.3684.
39. Ellebedy, A.H., Krammer, F., Li, G.M., Miller, M.S., Chiu, C., Wrammert, J., Chang, C.Y., Davis, C.W., McCausland, M., Elbein, R., et al. (2014). Induction of broadly cross-reactive antibody responses to the influenza HA stem region following H5N1 vaccination in humans. Proc. Natl. Acad. Sci. USA *111*, 13133–13138. https://doi.org/10.1073/pnas.1414070111.
40. Henry, C., Palm, A.K.E., Krammer, F., and Wilson, P.C. (2018). From Original Antigenic Sin to the Universal Influenza Virus Vaccine. Trends Immunol. *39*, 70–79. https://doi.org/10.1016/j.it.2017.08.003.
41. Elbe, S., and Buckland-Merrett, G. (2017). Data, disease and diplomacy: GISAID's innovative contribution to global health. Glob. Chall. *1*, 33–46. https://doi.org/10.1002/gch2.1018.
42. Swarnalekha, N., Schreiner, D., Litzler, L.C., Iftikhar, S., Kirchmeier, D., Künzli, M., Son, Y.M., Sun, J., Moreira, E.A., and King, C.G. (2021). T resident helper cells promote humoral responses in the lung. Sci. Immunol. *6*, eabb6808. https://doi.org/10.1126/sciimmunol.abb6808.
43. Wu, T., Hu, Y., Lee, Y.T., Bouchard, K.R., Benechet, A., Khanna, K., and Cauley, L.S. (2014). Lung-resident memory CD8 T cells (TRM) are indispensable for optimal cross-protection against pulmonary virus infection. J. Leukoc. Biol. *95*, 215–224. https://doi.org/10.1189/jlb.0313180.
44. Son, Y.M., Cheon, I.S., Wu, Y., Li, C., Wang, Z., Gao, X., Chen, Y., Takahashi, Y., Fu, Y.X., Dent, A.L., et al. (2021). Tissue-resident CD4(+) T helper cells assist the development of protective respiratory B and CD8(+) T cell memory responses. Sci. Immunol. *6*, eabb6852. https://doi.org/10.1126/sciimmunol.abb6852.
45. Lingwood, D., McTamney, P.M., Yassine, H.M., Whittle, J.R.R., Guo, X., Boyington, J.C., Wei, C.J., and Nabel, G.J. (2012). Structural and genetic basis for development of broadly neutralizing influenza antibodies. Nature *489*, 566–570. https://doi.org/10.1038/nature11371.
46. Yassine, H.M., McTamney, P.M., Boyington, J.C., Ruckwardt, T.J., Crank, M.C., Smatti, M.K., Ledgerwood, J.E., and Graham, B.S. (2018). Use of Hemagglutinin Stem Probes Demonstrate Prevalence of Broadly Reactive

Group 1 Influenza Antibodies in Human Sera. Sci. Rep. *8*, 8628. https://doi.org/10.1038/s41598-018-26538-7.

47. Jacob-Dolan, C., Hope, D., Liu, J., Waller-Pulido, A., Verrette, B., Cabrera-Barragan, D.N., Nangle, S.J., Wang, Q., Blanc, R., Fisher, J., et al. (2025). Adenovirus and mRNA vaccines as well as mucosal boosting improve protective efficacy against influenza virus challenge in macaques. Sci. Transl. Med. *17*, eadu7646. https://doi.org/10.1126/scitranslmed.adu7646.
48. Lasrado, N., Wang, L., Liu, J., Rössler, A., Chaudhari, J., Wang, Q., Stone, J.J., Granados-Contreras, F.A., Wu, J., Cabrera-Barragan, D.N., et al. (2025). An intramuscular prime and mucosal boost vaccine regimen protects against lethal clade 2.3.4.4b H5N1 challenge in cynomolgus macaques. Sci. Transl. Med. *17*, eady2282. https://doi.org/10.1126/scitranslmed.ady2282.
49. Kok, A., Wilks, S.H., Tureli, S., James, S.L., Bestebroer, T.M., Burke, D.F., Funk, M., van der Vliet, S., Spronken, M.I., Rijnink, W.F., et al. (2025). A vaccine central in A(H5) influenza antigenic space confers broad immunity. Nature *647*, 1005–1013. https://doi.org/10.1038/s41586-025-09626-3.
50. Sun, H., Deng, G., Sun, H., Song, J., Zhang, W., Li, H., Wei, X., Li, F., Zhang, X., Liu, J., et al. (2022). N-linked glycosylation enhances hemagglutinin stability in avian H5N6 influenza virus to promote adaptation in mammals. PNAS Nexus *1*, pgac085. https://doi.org/10.1093/pnasnexus/pgac085.
51. Ying, B., Darling, T.L., Desai, P., Liang, C.Y., Dmitriev, I.P., Soudani, N., Bricker, T., Kashentseva, E.A., Harastani, H., Raju, S., et al. (2024). Mucosal vaccine-induced cross-reactive CD8(+) T cells protect against SARS-CoV-2 XBB.1.5 respiratory tract infection. Nat. Immunol. *25*, 537–551. https://doi.org/10.1038/s41590-024-01743-x.
52. Roos, A., Roozendaal, R., Theeuwsen, J., Riahi, S., Vaneman, J., Tolboom, J., Dekking, L., Koudstaal, W., Goudsmit, J., and Radošević, K. (2015). Protection against H5N1 by multiple immunizations with seasonal influenza vaccine in mice is correlated with H5 cross-reactive antibodies. Vaccine *33*, 1739–1747. https://doi.org/10.1016/j.vaccine.2015.01.070.
53. Le Sage, V., Werner, B.D., Merrbach, G.A., Petnuch, S.E., O'Connell, A.K., Simmons, H.C., McCarthy, K.R., Reed, D.S., Moncla, L.H., Bhavsar, D., et al. (2025). Influenza A(H5N1) Immune Response among Ferrets with Influenza A(H1N1)pdm09 Immunity. Emerg. Infect. Dis. *31*, 477–487. https://doi.org/10.3201/eid3103.241485.
54. Warkentin, T.E., Baskin-Miller, J., Raybould, A.L., Sheppard, J.A.I., Daka, M., Nazy, I., and Moll, S. (2023). Adenovirus-Associated Thrombocytopenia, Thrombosis, and VITT-like Antibodies. N. Engl. J. Med. *389*, 574–577. https://doi.org/10.1056/NEJMc2307721.
55. Akula, V.R., Bhate, A.S., Gillurkar, C.S., Kushwaha, J.S., Singh, A.P., Singh, C., Pandey, A.K., K K, S., Rai, S.K., and Vadrevu, K.M.; BBV154 Study Group (2025). Effect of heterologous intranasal iNCOVACC((R)) vaccination as a booster to two-dose intramuscular Covid-19 vaccination series: a randomized phase 3 clinical trial. Commun. Med. *5*, 133. https://doi.org/10.1038/s43856-025-00818-6.
56. Singh, C., Verma, S., Reddy, P., Diamond, M.S., Curiel, D.T., Patel, C., Jain, M.K., Redkar, S.V., Bhate, A.S., Gundappa, V., et al. (2023). Phase III Pivotal comparative clinical trial of intranasal (iNCOVACC) and intramuscular COVID 19 vaccine (Covaxin((R))). npj Vaccines *8*, 125. https://doi.org/10.1038/s41541-023-00717-8.
57. Xu, J.W., Wang, B.S., Gao, P., Huang, H.T., Wang, F.Y., Qiu, W., Zhang, Y.Y., Xu, Y., Gou, J.B., Yu, L.L., et al. (2024). Safety and immunogenicity of heterologous boosting with orally administered aerosolized bivalent adenovirus type-5 vectored COVID-19 vaccine and B.1.1.529 variant adenovirus type-5 vectored COVID-19 vaccine in adults 18 years and older: a randomized, double blinded, parallel controlled trial. Emerg. Microbes Infect. *13*, 2281355. https://doi.org/10.1080/22221751.2023.2281355.
58. VanBlargan, L.A., Adams, L.J., Liu, Z., Chen, R.E., Gilchuk, P., Raju, S., Smith, B.K., Zhao, H., Case, J.B., Winkler, E.S., et al. (2021). A potently neutralizing SARS-CoV-2 antibody inhibits variants of concern by utilizing unique binding residues in a highly conserved epitope. Immunity *54*, 2399–2416.e6. https://doi.org/10.1016/j.immuni.2021.08.016.
59. Williams, G.D., Pinto, A.K., Doll, B., and Boon, A.C.M. (2016). A North American H7N3 Influenza Virus Supports Reassortment with 2009 Pandemic H1N1 and Induces Disease in Mice without Prior Adaptation. J. Virol. *90*, 4796–4806. https://doi.org/10.1128/JVI.02761-15.
60. Suguitan, A.L., Jr., Marino, M.P., Desai, P.D., Chen, L.M., Matsuoka, Y., Donis, R.O., Jin, H., Swayne, D.E., Kemble, G., and Subbarao, K. (2009). The influence of the multi-basic cleavage site of the H5 hemagglutinin on the attenuation, immunogenicity and efficacy of a live attenuated influenza A H5N1 cold-adapted vaccine virus. Virology *395*, 280–288. https://doi.org/10.1016/j.virol.2009.09.017.
61. Maizel, J.V., Jr., White, D.O., and Scharff, M.D. (1968). The polypeptides of adenovirus. I. Evidence for multiple protein components in the virion and a comparison of types 2, 7A, and 12. Virology *36*, 115–125.
62. Reynisson, B., Alvarez, B., Paul, S., Peters, B., and Nielsen, M. (2020). NetMHCpan-4.1 and NetMHCIIpan-4.0: improved predictions of MHC antigen presentation by concurrent motif deconvolution and integration of MS MHC eluted ligand data. Nucleic Acids Res. *48*, W449–W454. https://doi.org/10.1093/nar/gkaa379.
63. Yan, Z., Kim, K., Kim, H., Ha, B., Gambiez, A., Bennett, J., de Almeida Mendes, M.F., Trevizani, R., Mahita, J., Richardson, E., et al. (2024). Next-generation IEDB tools: a platform for epitope prediction and analysis. Nucleic Acids Res. *52*, W526–W532. https://doi.org/10.1093/nar/gkae407.

STAR★METHODS

KEY RESOURCES TABLE

REAGENT or RESOURCE	SOURCE	IDENTIFIER
**Antibodies**		
CR9114, anti-influenza A virus HA mAb	Cell Sciences	Cat: DVV03807A
AJ-4, anti-influenza A virus H5 HA mAb	This paper	N/A
SARS2-2, anti-SARS-CoV-2 MAb	Van Blargan et al.[58]	N/A
SARS2-11	Van Blargan et al.[58]	N/A
SARS2-16	Van Blargan et al.[58]	N/A
SARS2-31	Van Blargan et al.[58]	N/A
SARS2-38	Van Blargan et al.[58]	N/A
SARS2-57	Van Blargan et al.[58]	N/A
SARS2-71	Van Blargan et al.[58]	N/A
Alexa Fluor 647 anti-human IgG	Thermo Fisher	Cat: A21445; RRID: AB_ 2535862
Alexa Fluor 647 anti-mouse IgG	Thermo Fisher	Cat: A21235; RRID: AB_2535804
HRP-conjugated goat anti-human IgG	Sigma	Cat: A6029; RRID: AB_258272
APC/Fire750 anti-CD45 mAb	BioLegend	Cat: 103154; RRID: AB_2572116
BV605 anti-mouse CD45	BioLegend	Cat: 103139; RRID: AB_2562341
BV785 anti-mouse CD4	BioLegend	Cat: 100453; RRID: AB_2565843
PerCP/Cyanine 5.5 anti-mouse CD8b	BioLegend	Cat: 126610; RRID: AB_2260149
PE/Cyanine 7 anti-mouse/human CD44	BioLegend	Cat: 103030; RRID: AB_830787
FITC anti-mouse CD69	BioLegend	Cat: 104505; RRID: AB_313108
BV421 anti-mouse CD103	BioLegend	Cat: 121421; RRID: AB_10900074
APC anti-mouse IFN-gamma	BioLegend	Cat: 505810; RRID: AB_315404
PE anti-mouse TNF	Invitrogen	Cat:12-7321-82; RRID: AB_466199
InViVoMAb anti-mouse CD4	Bio X cell	Cat: BE0003-1; RRID: AB_1107636
InViVoMAb anti-mouse CD8-beta	Bio X cell	Cat: BE0223; RRID: AB_2687706
InViVoMAb anti-horseradish peroxidase	Bio X cell	Cat: BE0088; RRID: AB_1107775
**Bacterial and virus strains**		
H5N1 A/Michigan/90/2024	This paper	N/A
H5N1 A/Texas/37/2024	Dr. Richard Webby at St Jude Children's Hospital	N/A
"6 + 2" H5N1/PR8 virus (HA and NA from A/Michigan/90/2024)	This paper	N/A
ChAd-CTRL	Hassan et al.[30]	N/A
ChAd-SARS-CoV-2-S	Hassan et al.[30]	N/A
ChAd-H5-Texas	This paper	N/A
ChAd-H5-Texas-RR (I45R + T49R)	This paper	N/A
ChAd-H5-Vietnam	This paper	N/A
ChAd-H5-Fujian	This paper	N/A
**Chemicals, peptides, and recombinant proteins**		
H5-HA protein (A/Texas/37/2024)	This paper	N/A
H5-HA protein (A/Vietnam/KhanhhoaRV1-005/2024)	This paper	N/A
H5-HA protein (A/Fujian/2/2004)	This paper	N/A
H5-HA (A/Texas/37/2024-I45R-T49R)	This paper	N/A
H1-HA protein (A/Delaware/55/2019)	This paper	N/A
H3-HA protein (A/Darwin/11/2021)	This paper	N/A
HA protein (B/Singapore/WU4618/2021)	This paper	N/A
HA protein (B/Singapore/INFTT-16-0610/2016)	This paper	N/A

(*Continued on next page*)

Continued

REAGENT or RESOURCE	SOURCE	IDENTIFIER
H1 HA-stem (A/California/04/2009)	This paper	N/A
EZ-Link NHS-PEG4-Biotin	Thermo Fisher	Cat: A39259
Streptavidin Coated Fluorescent Yellow Particle Kit	Spherotech	Cat: SVFA-2552-6K
Streptavidin coated magnetic beads	BioLabs	Cat: S1420S
Experimental models: Cell lines		
Madin-Darby canine kidney (MDCK) cells	ATCC, CCL-34	N/A
HEK293 cells	ATCC, CRL-1573;	RRID: CVCL_0045
HEK293T cells	ATCC, CRL-3216;	RRID: CVCL_0063
Experimental models: Organisms/strains		
C57BL/6J mice	Jackson laboratory	strain # 000664
Syrian hamsters	Charles River and Inotiv	strain # 049; HsdHan®:AURA
Oligonucleotides		
H5N1 *M* gene qPCR: F-primer 5′- CTTCTAACCGAGGTCGA AACGTA -3′	This paper	N/A
H5N1 *M* gene qPCR: R-primer 5′- GGTGACAGGATTGGTCT TGTCTTTA -3′	This paper	N/A
H5N1 *M* gene qPCR probe: 5’-/56-FAM/TCAAGGAAC/Zen/ TCGGGCCCCCTCAAAGCCGAG/3’/IBFQ	This paper	N/A
Software and algorithms		
GraphPad Prism	GraphPad	version 10.0.3
Adobe Illustrator	Adobe	version 24.2.3
NDP.View2	Hamamatsu	N/A
Biorender	Biorender.com	N/A
FlowJo	BD Biosciences	version 10.8.0

EXPERIMENTAL MODEL AND SUBJECT DETAILS

Cells

Madin-Darby canine kidney (MDCK) cells (American Type Culture Collection, CCL-34) were cultured at 37°C in Dulbecco’s Modified Eagle medium (DMEM) supplemented with 10% fetal bovine serum (FBS), 10 mM HEPES pH 7.3, 1 mM sodium pyruvate, 1x non-essential amino acids, and 100 U/mL of penicillin–streptomycin.

Viruses

A molecular clone of HPAI H5N1 virus A/Michigan/90/2024 (clade 2.4.4.4b) virus was produced by reverse genetics using the pHW2000 system. The eight gene segments (GenBank: PB2 (PP839262.1), PB1 (PP839264.1), PA (PP839259.1), HA (PP839265.1), NP (PP839258.1), NA (PP839261.1), M (PP839263.1), NS (PP839260.1) were cloned into the pHW2000 plasmid, sequence confirmed, and used to generate infectious virus as described previously (Williams et al., 2016). Following the rescue, the virus was propagated in MDCK cells and titrated by focus forming assay (FFA). The HPAI H5N1 A/Texas/37/2024 (clade 2.3.4.4b) virus was obtained from Dr. Richard Webby (St. Jude Children’s Research Hospital, Memphis TN). The virus was propagated in MDCK cells and titrated by FFA. All virus stocks were subjected to next-generation sequencing (NGS) to confirm the identity and sequence of the virus. All experiments with HPAI H5N1 virus were performed in an approved animal biosafety level 3 plus (ABSL-3+) facility.

A reassortant low-pathogenic “6 + 2” H5N1 virus encoding the HA and NA of H5N1 A/Michigan/9/2024 on a A/Puerto Rico/8/1934 (PR8) backbone was generated using a reverse genetics system.[59] The polybasic cleavage site in the H5 HA gene, REKRRKRGL, was replaced with RGTRGL,[60] and the “6 + 2” virus was generated in our ABSL-3+ facility. The infectious virus was expanded once on MDCK cells in the presence of TPCK trypsin, and the dependence on trypsin for growth and expansion of this LPAI H5N1 “6 + 2” virus was confirmed. The virus stock was sequenced to confirm the presence of six gene segments of PR8, two gene segments of A/Michigan/90/2024, and the trypsin-dependent HA cleavage site. Upon approval by our institutional biosafety committee, this virus was transported to our approved BSL-2+ facility and used for virus neutralization assays.

Animals

Seven-to 8-week-old C57BL/6 female mice were obtained from The Jackson Laboratory. Five-to 6-week old Syrian hamsters were obtained from Charles River Laboratories or Inotiv and housed at Washington University School of Medicine. Animal studies were

carried out in accordance with the recommendations in the Guide for the Care and Use of Laboratory Animals of the National Institutes of Health. The protocols were approved by the Institutional Animal Care and Use Committee at the Washington University School of Medicine (Assurance #A3381–01). Virus inoculations were performed under anesthesia with ketamine hydrochloride and xylazine or isoflurane (mice) or isoflurane (hamsters), and all efforts were made to minimize animal suffering. Experiments were neither randomized nor blinded.

METHOD DETAILS

Construction of chimpanzee adenoviral vectors

A pSAd36 genomic plasmid was described previously[30] and used for ChAd-vector construction. Briefly, H5 HA sequences corresponding to different HPAI H5 strains from clade 2.3.4.4b, 2.3.2.1c, and 2.3.4.4h (GISAID: EPI_ISL_19027114, EPI_ISL_19031556, EPI_ISL_19145664) were codon-optimized for expression in *Mus musculus* cells and cloned into pSAd36 plasmid under the CMV-tetO2 promoter control to generate pSAd36-HA. In parallel, a pSAd36-control plasmid without transgene insertion was generated. The pSAd36-HA and pSAd36-control plasmids were linearized and transfected into T-Rex 293-HEK cells (Invitrogen). The rescued replication-incompetent ChAd-H5 HA and ChAd-CTRL vectors were scaled up in 293 cells and purified by CsCl density-gradient ultracentrifugation. Virus particle (vp) concentration in each vaccine preparation was determined by spectrophotometry at 260 nm based on a well-established conversion factor (viral particles per mL = $OD_{260nm} \times 1.1 \times 10^{12}$) as described.[61] To confirm that the H5 HA protein was expressed and antigenically intact, 293T cells were transduced with 20 virus particles per cell of ChAd-SARS-CoV-2-S, ChAd-Texas, ChAd-Vietnam or ChAd-Fujian. At 40 h post-infection, cells were fixed with 4% paraformaldehyde at 4° C for 10 min and subsequently stained with an oligoclonal pool of spike-reactive monoclonal antibodies SARS2-2, SARS2-11, SARS2-16, SARS2-31, SARS2-38, SARS2-57, and SARS2-71 SARS-CoV-2,[58] or HA stem-reactive mAb (CR9114).

Generation of recombinant influenza virus HA proteins

Sequences for HA (Table S1) were codon-optimized for expression in a human cell line using the GenSmart tool and synthesized by Twist Bioscience. Each HA construct included a T4 foldon trimerization domain at the C-terminus followed by an Avi-tag and a C-terminal 6×His tag. Plasmids were purified using the ZymaPURE Plasmid Maxiprep Kit (Zymo Research) and transiently transfected into Expi293F suspension cells cultured in FreeStyle 293 expression medium (Thermofisher). Cells were seeded at a density of 3×10^6 cells/mL and transfected using linear 25 kDa polyethylenimine (PEI, Polysciences) at a DNA: PEI ratio of 1:4 (w/w). Plasmid DNA and PEI were separately diluted in Opti-MEM, mixed, and incubated for 15 min at room temperature before being added to the culture. Transfected cultures were incubated at 37°C, 8% CO_2, and 125 rpm for 5 days. Proteins were purified from clarified supernatant using a HisTrap Excel column and eluted with 250 mM imidazole. Proteins were concentrated using Amicon Ultra centrifugal filters (10 kDa MWCO, Millipore) and further purified by size exclusion chromatography (SEC) using a Superdex 200 Increase 10/300 GL column (Cytiva) equilibrated with PBS (pH 7.4). Peak fractions were pooled and analyzed by SDS-PAGE.

HA multiplex binding assays

Recombinant HA proteins from H5 Texas (A/Texas/37/2024), H5 Vietnam (A/Vietnam/KhanhhoaRV1-005/2024), H5 Fujian (A/Fujian/2/2004), H5 Texas-RR (I45R and T49R substitutions), as well as HA proteins from seasonal influenza viruses, A/Delaware/55/2019 (H1), A/Darwin/11/2021 (H3), B/Singapore/WU4618/2021 (B-Vic), B/Singapore/INFTT-16-0610/2016 (B-Yam), H1 HA-stem (H1-stem, based on A/California/04/2009) were biotinylated separately with EZ-Link NHS-PEG4-Biotin (ThermoFisher, A39259) for 2 h at 4°C and purified through Zeba spin desalting columns (ThermoFisher) to remove unbound biotin. Each biotinylated HA protein was subsequently incubated with the SPHERO streptavidin-coated fluorescent yellow particles with different fluorescence intensity peaks (Spherotech, SVF3-2552) at 20 ng HA per μg beads for 45 min at room temperature. Free biotin (5 μM, Avidity, BIO200) was then added to the beads and incubated for 15 min at room temperature. Beads of different fluorescence intensity loaded with different proteins were then pooled and washed with FACS buffer (PBS supplemented with 2% FBS, 2 mM ethylenediaminetetraacetic acid (EDTA), and 0.1% NaN_3) followed by incubation with serially diluted serum samples or anti-HA stem CR9114 mAb for 30 min at room temperature. Beads were washed twice with FACS buffer and subsequently stained with an anti-mouse IgG Alexa Fluor 647 (ThermoFisher, A-21235, 1:3,000) for 10 min at room temperature in the dark. After two washes with FACS buffer, beads were resuspended in FACS buffer and acquired on an iQue3 flow cytometer (Sartorius). The mean fluorescent intensity (MFI) was recorded. The geometric MFI of no-antibody control wells was defined as the background signal. The endpoint serum dilution (EC_{50}) was calculated with curve fit analysis of MFI values for serially diluted sera with a cut-off value set to the mean of the background signal plus five times standard deviation.

Antibody depletion assay

Streptavidin coated magnetic beads (BioLabs, Cat. #S1420S) were washed twice with PBST and incubated with biotinylated recombinant HA proteins (18 μg per mg beads in PBS): H1N1 (A/Delaware/55/2019), H3N2 (A/Darwin/11/2021) or H1-stem (A/California/04/2009). Protein-bead complexes were incubated for 30 min at room temperature on an end-over-end mixer. Beads were washed

twice with PBS and incubated with diluted sera samples for 30 min at room temperature with agitation. Depleted sera were collected on a KingFisher Flex extraction robot (ThermoFisher). The levels of antibody binding to HA proteins of H1, H3, H1 stem and H5-Texas were measured using HA multiplex binding assay.

Virus neutralization assay

Mouse and hamster sera were treated with receptor destroying enzyme (RDE, Taikan) at a 1:3 ratio of serum to RDE overnight at 37°C. The enzyme was inactivated by heat (56°C for 30 min), and 6 volumes of PBS were added to each sample for a final dilution of 1:10. Serial dilutions of mouse and hamster sera or BALF were incubated with 10^2 FFU of "6 + 2" LPAI H5N1 virus (A/Michigan/90/2024) for 1 h at 37°C. Antibody-virus complexes were added to MDCK cell monolayers in 96-well plates and incubated at 37°C for 1 h. Subsequently, cells were washed once with PBS and overlaid with 1% (w/v) methylcellulose in MEM containing 1.0 μg/mL of TPCK trypsin. Plates were harvested 24 h later by removing overlays and fixing with 4% PFA in PBS for 20 min at room temperature. Plates were washed and sequentially incubated with CR9114 anti-HA stem mAb and HRP-conjugated goat anti-human IgG (Sigma Cat # A6029, 1:1000) in PBS supplemented with 0.1% saponin and 0.1% bovine serum albumin. Influenza virus infected cell foci were visualized using TrueBlue peroxidase substrate (KPL) and quantitated on an ImmunoSpot microanalyzer (Cellular Technologies).

Mouse immunization and challenge experiments

In the initial studies to assess the immunogenicity and protective activity of ChAd-H5 vaccines, cohorts of 7-week-old female C57BL/6 mice were immunized once IN with ChAd-CTRL, ChAd-Texas, ChAd-Vietnam, ChAd-Fujian, or a ChAd-trivalent vaccine (a 1:1:1 mixture of ChAd-Texas, ChAd-Vietnam, and ChAd-Fujian) at a dose of 10^{10} vp in 50 μL of PBS. Four weeks later, animals were bled to assess antibody responses. To evaluate protective efficacy, mice were challenged IN five weeks after immunization with 20 FFU (~20 LD_{50}) of HPAI H5N1 (A/Michigan/90/2024) virus, and body weights were monitored daily. At 6 dpi, animals were euthanized, and nasal wash, nasal turbinates, and left lung lobes were collected for virological analysis. The right lung lobes were harvested for pathological evaluation. In separate experiments, the amount of vaccine (ChAd-Texas or ChAd-CTRL) was reduced to 10^9, 10^8, 10^7, or 10^6 vp and administered via IN or IM inoculation, and the challenge dose was increased to 2,000 or 20,000 FFU of A/Michigan/90/2024.

Seasonal vaccine or H1 protein prior exposure experiments in mice. Cohorts of 7-to 8-week-old female C57BL/6 mice were immunized IM in the hind leg with 1.5 μg of the Flucelvax Quadrivalent Seasonal Influenza vaccine (QIV, 2022–2023 Formula) mixed with AddaVax, or PBS in a total volume of 100 μL. The vaccine targets four seasonal influenza viruses and contains 30 μg/mL of each HA antigen listed below: A/Delaware/55/2019 CVR-45 (an A/Wisconsin/588/2019 (H1N1)pdm09-like virus), A/Darwin/11/2021 (a A/Darwin/6/2021 (H3N2)-like virus), B/Singapore/WUH4618/2021 (a B/Austria/1359417/2021-like virus), and B/Singapore/INFTT-16-0610/2016 (a B/Phuket/3073/2013-like virus). Animals were bled three weeks after the second QIV dose for immunogenicity analysis. Four weeks after the final QIV dose, mice were boosted IN with 10^9 vp of ChAd-CTRL, ChAd-Texas, or ChAd-Texas-RR vaccine which incorporates two arginine mutations (I45R and T49R) in the stem region of the H5 Texas HA to disrupt cross-reactive stem-specific antibody responses. Serum samples were collected three weeks after the ChAd-vaccine boost to evaluate antibody responses. Six weeks after ChAd-vaccine boost, mice were challenged IN with 20,000 FFU of H5N1 A/Michigan/90/2024. Body weights were monitored daily, and tissues were collected at 5 dpi for virological analyses. In a second experiment, cohorts of mice were immunized and challenged using the same regimen except that the mice were primed intramuscularly with 3 μg of the H1 HA antigen (A/H1N1/New Caledonia/1999) before boosting with QIV plus Addavax and the ChAd-Texas vaccine.

The timing of tissue collection was optimized for each animal model based on viral kinetics while minimizing the risk of animal loss due to lethality. Tissues were collected at 6 dpi in the initial study in which animals were challenged with a lower virus dose (20 FFU). In other studies using a higher lethal challenge dose (2×10^4 FFU) 5 dpi was selected due to the accelerated infection kinetics and possible survivor bias at 6 dpi.

Hamster experiments

Cohorts of 5- to 6-week-old male Syrian golden hamsters from Charles Rivers Laboratories or Inotiv were immunized once IN with 10^{10} vp of ChAd-Texas or ChAd-CTRL in 50 μL of PBS. Four weeks later, animals were bled to assess antibody responses. To evaluate protective efficacy, hamsters were challenged IN five weeks post-immunization with 10^5 FFU of A/Michigan/90/2024 or 10^4 of A/Texas/37/2024, and body weights were recorded daily. At 4 dpi, animals were euthanized, and lungs, nasal turbinates, and brain were collected for virological analysis.

Measurement of viral burden

Tissues were homogenized in 1 mL of MEM, clarified by centrifugation (1,000 × *g* for 5 min) and used for viral titer analysis by quantitative RT-qPCR and by FFA assay. Infectious virus titers were measured by FFA. Serial dilutions of virus stocks or tissue homogenates were added to MDCK cell monolayers in 96-well plates and incubated at 37°C for 1 h. Subsequently, cells were washed once with PBS and overlaid with 1% (w/v) methylcellulose in MEM. Plates were harvested 24 h later by removing overlays and fixing with 4% PFA in PBS for 20 min at room temperature. Plates were washed and sequentially incubated with CR9114 anti-HA stem mAb and

HRP-conjugated goat anti-human IgG (Sigma Cat # A6029, 1:1000) in PBS supplemented with 0.1% saponin and 0.1% bovine serum albumin. Influenza virus infected cell foci were visualized using TrueBlue peroxidase substrate (KPL) and quantitated on an ImmunoSpot microanalyzer (Cellular Technologies).

Total RNA was extracted from 100 μL of the homogenate using the MagMAX Viral/Pathogen Nucleic Acid Isolation kit (A48310, Thermo Fisher Scientific) on the Kingfisher Flex extraction robot (Thermo Fisher Scientific) and eluted in 50 μL of water. Four microliters of RNA were used for real-time RT-qPCR to detect and quantify *M* gene of H5N1 A/Michigan/90/2024 using TaqMan RNA-to-CT 1-Step Kit (Thermo Fisher Scientific) using the following primers and probes: Forward: CTTCTAACCGAGGTCGAAACGTA; Reverse: GGTGACAGGATTGGTCTTGTCTTTA; Probe: TCGGGCCCCCTCAAAGCCGAG; 5′Dye/3′Quencher: 6-FAM/ZEN/IBFQ. Reverse transcription was carried out at 48°C for 15 min followed by 2 min at 95°C. Amplification was accomplished over 50 cycles as follows: 95°C for 15 s and 60°C for 1 min. Viral RNA was expressed as *M* gene copy numbers per mL for tissue homogenates and was based on a standard included in the assay created via *in vitro* transcription of a synthetic DNA molecule containing the target region of the *M* gene.

Lung histology and viral RNA *in situ* hybridization

Lungs (right lobes) of euthanized mice were inflated with 1 mL of 10% neutral buffered formalin using a 1-mL syringe after a catheter was inserted into the trachea. Lungs were then kept in fixative for 7 days. Tissues were embedded in paraffin, and sections were stained with hematoxylin and eosin for histopathological evaluation. For *in situ* RNA hybridization, RNAscope probes targeting nucleoprotein (*NP*) mRNA of clade 2.3.4.4b H5N1 (A/Michigan/90/2024) (V-H5N1-NP) was custom-designed (Advanced Cell Diagnostics) and consisted of 20 antisense oligopairs. RNAscope *in situ* hybridization assays were assessed using RNAscope 2.5 high-definition brown assay according to the manufacturer's recommendations. Briefly, paraffin-embedded lung sections were deparaffinized by incubating at 60°C for 1 h, and endogenous peroxidases were quenched with H_2O_2 for 10 min at room temperature. Slides were boiled for 15 min in RNAscope Target Retrieval Reagents and incubated for 30 min in RNAscope Protease Plus reagent prior to viral RNA probe (Advanced Cell Diagnostics 848561) hybridization and signal amplification. Sections were counterstained with Gill's hematoxylin. Images were captured using the Nanozoomer (Hamamatsu) at the Alafi Neuroimaging Core at Washington University.

H5 HA CD4 and CD8 T cell epitopes prediction

We scanned the A/Texas/37/2024 H5 HA protein sequence (GenBank: WPD27583.1) using NetMHCpan 4.1 EL[62] implemented on the IEDB analysis resource[63] to predict peptides binding to H2-Kb and H2-Db alleles (class I MHC, 8–11-mers). Peptides with a predicted percentile rank in the top 0.5 percentile rank were considered, and duplicate peptides selected for both Kb and Db were removed. The top 8 peptides for Db and 8 for Kb were selected for a total of 16. For class II MHC, 15-mer peptides were considered and ranked using NetMHCIIPan 4.1 EL. 49 peptides in the top 10 percentile rank were considered, and peptides sharing the same predicted binding core were removed, resulting in 14 peptides. All peptides are listed in Table S2.

T cell analysis

Seven-week-old female C57BL/6 mice were immunized IN with 10^9 vp of ChAd-CTRL or ChAd-Texas and boosted four weeks later IN with the same dose. BALF, spleen, and lung tissues were collected on day 10 after the 2nd dose. To discriminate circulating from extravascular parenchymal immune cells, mice anesthetized with an overdose of ketamine and xylazine were intravenously administered 2 μg of APC/Fire750-labeled anti-CD45 mAb (clone 30-F11; cat: 103154; BioLegend). After 3 min of *in vivo* labeling, mice were euthanized. BALF was collected by inserting a plastic catheter into the trachea and performing three sequential lavages with 800 μL of PBS. Pooled BALF was centrifuged at 600 × g for 5 min at 4°C, and the supernatant was stored at −20°C for subsequent analysis. Lungs were collected in DMEM supplemented with 10% FBS on ice, minced with scissors, and passed through 70 μm cell strainers. The resulting cell suspension was digested in HBSS containing 25 μg/mL DNase I (cat: 11284932001; Roche) and 50 μg/mL Liberase (cat: 5401119001; Roche) for 30 min at 37°C. Following hypotonic erythrocyte lysis, single cells were filtered again through 70 μm cell strainers. Single cell splenocytes were generated by mechanical disruption and filtration through a 70 μm cell strainer, followed by red blood cell lysis using ACK lysis buffer (cat: A10492-01; Gibco) without Liberase/DNase I treatment. All cells were resuspended in RPMI 1640 medium supplemented with 10% FBS, 1% HEPES, 1% L-glutamine, and 0.1% β-mercaptoethanol.

For peptide restimulation, cells were incubated overnight at 37°C with either a pool of MHC class I (Table S2; 2.0 μg/mL per peptide), or a combination of two-pre-tested MHC class II restricted peptides of $HA_{204-218}$; TNLYKNPITYISVGT and HA_{90-104}; PEWSYIVERANPAND (4.0 μg/mL per peptide) in the presence of Brefeldin A (1:500; Invitrogen). The following day, cells were washed and stained with Fc block (clone 93; cat: 101302; BioLegend), and surface markers including CD45 (BV605; clone 30-F11; cat: 103139; BD Biolegend), CD4 (BV785; clone GK1.5; cat: 100453; BioLegend), CD8β (PerCP/Cy5.5; clone YTS156.7.7; cat: 126610; BioLegend), CD44 (PE/Cy7; clone IM7; cat: 103030; BioLegend), CD69 (FITC; clone H1.2F3; cat: 104505; Biolegend) and CD103 (BV421; clone 2E7; cat: 121421; Biolegend) for 30 min at 4°C in FACS buffer (1× PBS with 2% FBS and 2 mM EDTA). Dead cells were excluded using Live/Dead viability dye (efluor506, cat: 65-0866-14; Invitrogen), which was added during surface staining. Subsequently, cells were washed, fixed, and permeabilized using the BD Fixation/Permeabilization Kit (cat: 554714; BD Biosciences) according to the manufacturer's protocol. Intracellular staining was performed for IFN-γ (APC; clone XMG1.2; cat: 505810; BioLegend), TNF (PE; clone MP6-XT22; cat: 12-7321-82; Invitrogen). Samples were acquired using a Cytek Aurora flow cytometer and analyzed with FlowJo software (version 10.4.2). Precision counting beads (BioLegend; Cat: 424902) were added to each well

before acquisition of the sample on flow cytometer and absolute cell numbers were obtain by comparing bead count and cell count according to manufacturer's protocol.

T cells depletion experiments

Cohorts of seven-week-old female C57BL/6 mice were immunized IN with a single dose (10^8 vp) of ChAd-CTRL or ChAd-Texas vaccine. The mice were treated with an anti-mouse CD4 antibody (Bio X cell, BE0003-1, clone GK1.5), anti-mouse CD8β antibody (Bio X cell, BE0223, clone 53–5.8), or with a sham isotype-control antibody (rat IgG1 isotype, anti-horseradish peroxidase, Bio X cell, # BE0088) via intraperitoneal (250 μg in 100 μL PBS) in combination with IN administration (10 μg in 50 μL PBS) using two different regimens): (1) For $CD8^+$ T cell depletion, anti-CD8β antibodies were administered intraperitoneally (IP) one day before vaccination and three days after IN ChAd-Texas immunization. A second round of antibody treatment was given on days 30 and 33 after vaccination, corresponding to six and three days before A/Michigan/90/2024 virus challenge. (2) For $CD4^+$ T cell depletion, intraperitoneal (IP) and intranasal (IN) injections of anti-CD4 antibodies were administered on days 30 and 33 after vaccination to avoid affecting the T cell-dependent antibody response. On day 34, sera were collected for antibody analysis, and spleens and lungs were analyzed for $CD4^+$ and $CD8^+$ T cells by flow cytometry. Thirty-six days after vaccination, mice were challenge with 20,000 FFU of H5N1 A/Michigan/37/2024 virus by intranasal administration. Daily weights were recorded, and at 5 dpi, nasal turbinates, lungs, and brains were collected for viral burden analysis.

QUANTIFICATION AND STATISTICAL ANALYSIS

Statistical analysis was performed using GraphPad Prism version 10 and significance was assigned when p values were <0.05. Statistical tests, number of animals, mean, median or geometric mean values, and statistical comparison groups are indicated in the Figure legends. Log-transformed viral RNA levels, serum antibody, or cellular responses were used in the statistical analysis.

Cell Reports Medicine, Volume *7*

Supplemental information

An intranasal adenoviral-vectored vaccine protects against highly pathogenic avian influenza H5N1 in naive and antigen-experienced animals

Baoling Ying, Kelly Pyles, Tamarand L. Darling, Kuljeet Seehra, Truc Pham, Lin-Chen Huang, Houda H. Harastani, Ashish Sharma, Pritesh Desai, Elena A. Kashentseva, David T. Curiel, Bjoern Peters, James Brett Case, Eva-Maria Strauch, Michael S. Diamond, and Adrianus C.M. Boon

Supplemental information

An intranasal adenoviral-vectored vaccine protects against highly pathogenic avian influenza H5N1 in naïve and antigen-experienced animals

Baoling Ying, Kelly Pyles, Tamarand L Darling, Kuljeet Seehra, Truc Pham, Lin-Chen Huang, Houda H. Harastani, Ashish Sharma, Pritesh Desai, Elena A. Kashentseva, David T. Curiel, Bjoern Peters, James Brett Case, Eva-Maria Strauch, Michael S. Diamond, and Adrianus C.M. Boon

Supplementary Figure S1

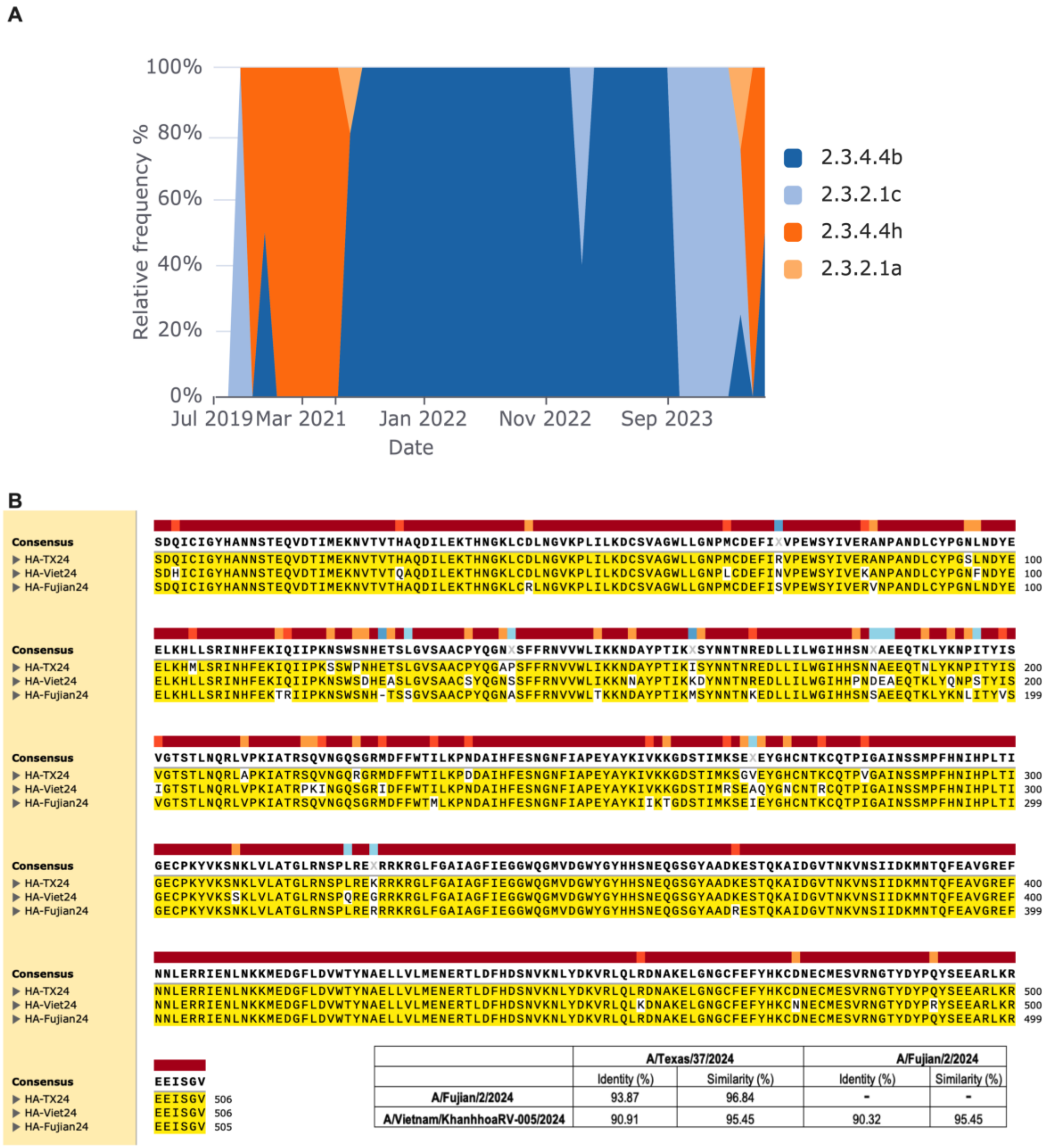

	A/Texas/37/2024		A/Fujian/2/2024	
	Identity (%)	Similarity (%)	Identity (%)	Similarity (%)
A/Fujian/2/2024	93.87	96.84	-	-
A/Vietnam/KhanhhoaRV-005/2024	90.91	95.45	90.32	95.45

Figure S1. Genetic and sequence analysis of HA proteins from recently circulating H5-Texas, H5-Vietnam and H5-Fujian strains, Related to Figure 1. (**A**) Relative clade frequency of human infecting HPAI H5 influenza A viruses circulating between June 2021 and April 2024. (**B**) Sequence alignment of HA proteins from the H5-Texas, H5-Vietnam, and H5-Fujian strains. The percentages of identity and similarity among the strains are shown in the Table.

Supplementary Figure S2

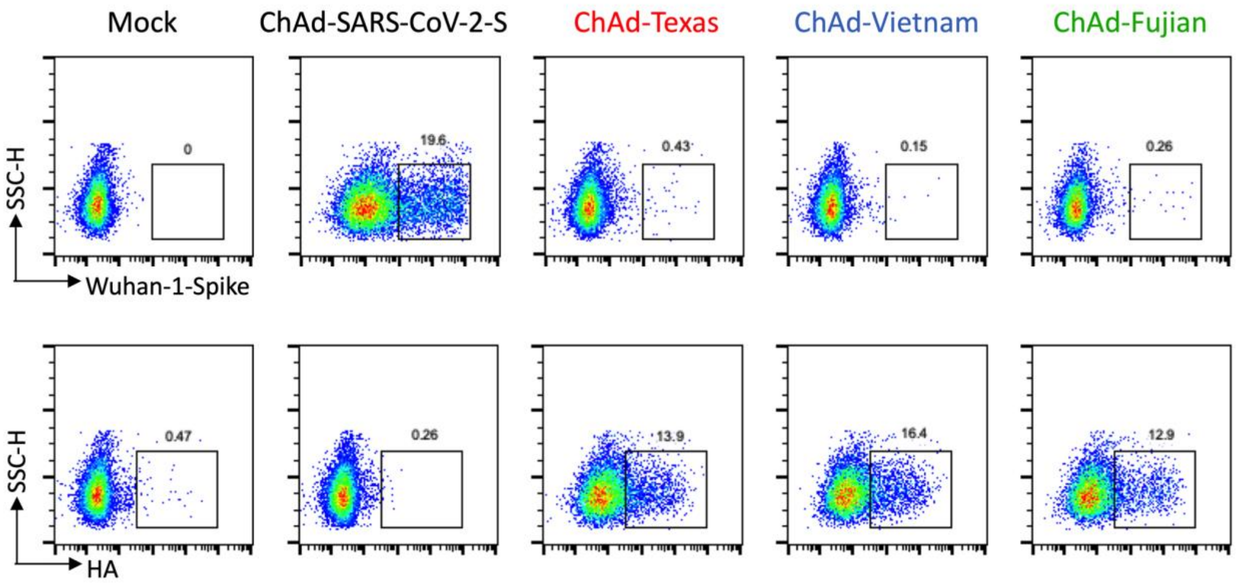

Figure S2. Surface expression of H5 HA from ChAd-Texas (clade 2.3.4.4b), ChAd-Vietnam (clade 2.3.2.1c) and ChAd-Fujian (clade 2.3.4.4h) transduced 293T cells, Related to Figures 1 and 2. 293T cells were transduced with 20 virus particles per cell of ChAd-SARS-CoV-2-S, ChAd-Texas, ChAd-Vietnam or ChAd-Fujian. At 40 h post-infection, cells were fixed and stained with an oligoclonal pool of spike-reactive monoclonal antibodies SARS2-2, SARS2-11, SARS2-16, SARS2-31, SARS2-38, SARS2-57, and SARS2-71 SARS-CoV-2 (VanBlargan et al., 2021), or HA stem-reactive mAb (CR9114). Representative flow cytometry plots of surface staining are shown.

Supplementary Figure S3

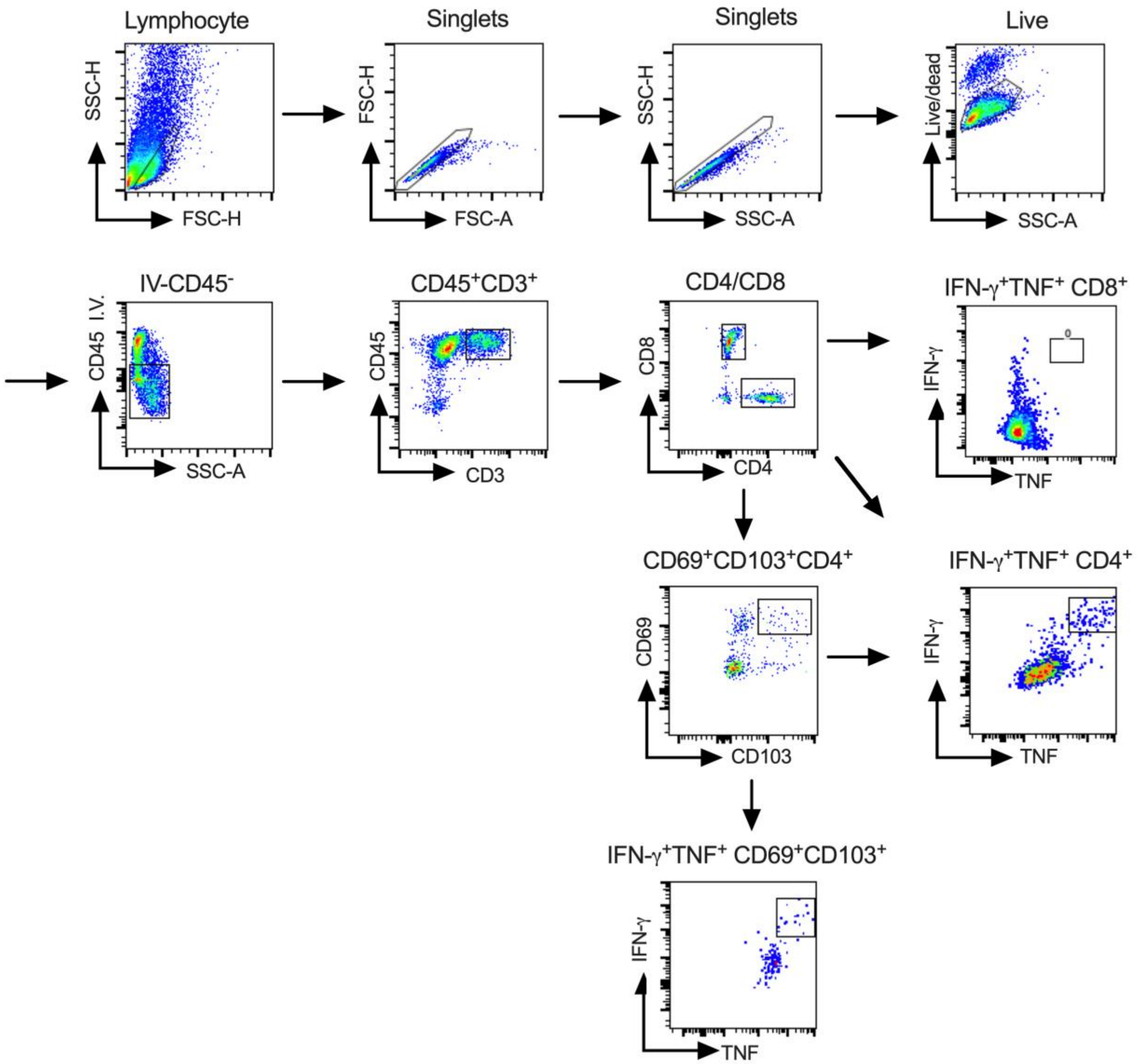

Figure S3. Gating strategy for T cell responses, Related to Figure 3 and S6. Seven-week-old female C57BL/6 mice were immunized with ChAd-CTRL or ChAd-Texas and boosted four weeks later. T cell responses in the spleen and lungs at day 10 post-boost were analyzed after HA peptide re-stimulation. Cells were gated for lymphocytes (FSC-A/SSC-A), singlets (SSC-W/SSC-H), live cells (Viability dye eF506$^-$), IV-CD45$^-$, CD45$^+$ CD3$^+$, CD4$^+$ or CD8$^+$ followed by CD4$^+$ or CD8$^+$ cell populations expressing IFN-γ and TNF. HA-specific resident memory CD4$^+$ T cells (CD4 T_{RM}) in the lung were gated on CD4$^+$CD69$^+$CD103$^+$, followed by staining of IFN-γ $^+$ and TNF$^+$.

Supplementary Figure S4

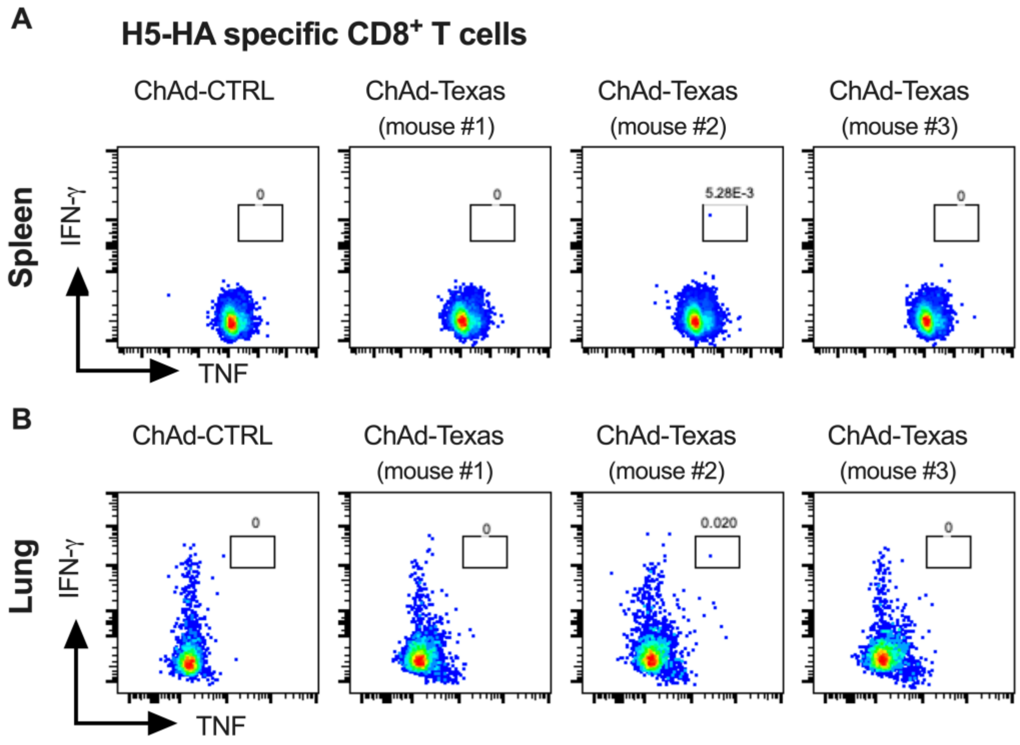

Figure S4. Lack of measurable CD8$^+$ T cell responses following IN immunization with ChAd-Texas vaccine in C57BL/6 mice, Related to Figure 3. Seven-week-old female C57BL/6 mice were immunized IN with 10^9 vp of ChAd-CTRL or ChAd-Texas and boosted four weeks later. On day 10 post-boost, CD8$^+$ T cell responses in the spleen and lungs were analyzed following HA peptide re-stimulation. Representative flow cytometry plots of IFN-γ^+ and TNF$^+$ CD8$^+$ T cells in the spleen (**A**) and lung (**B**) are shown.

Supplementary Figure S5

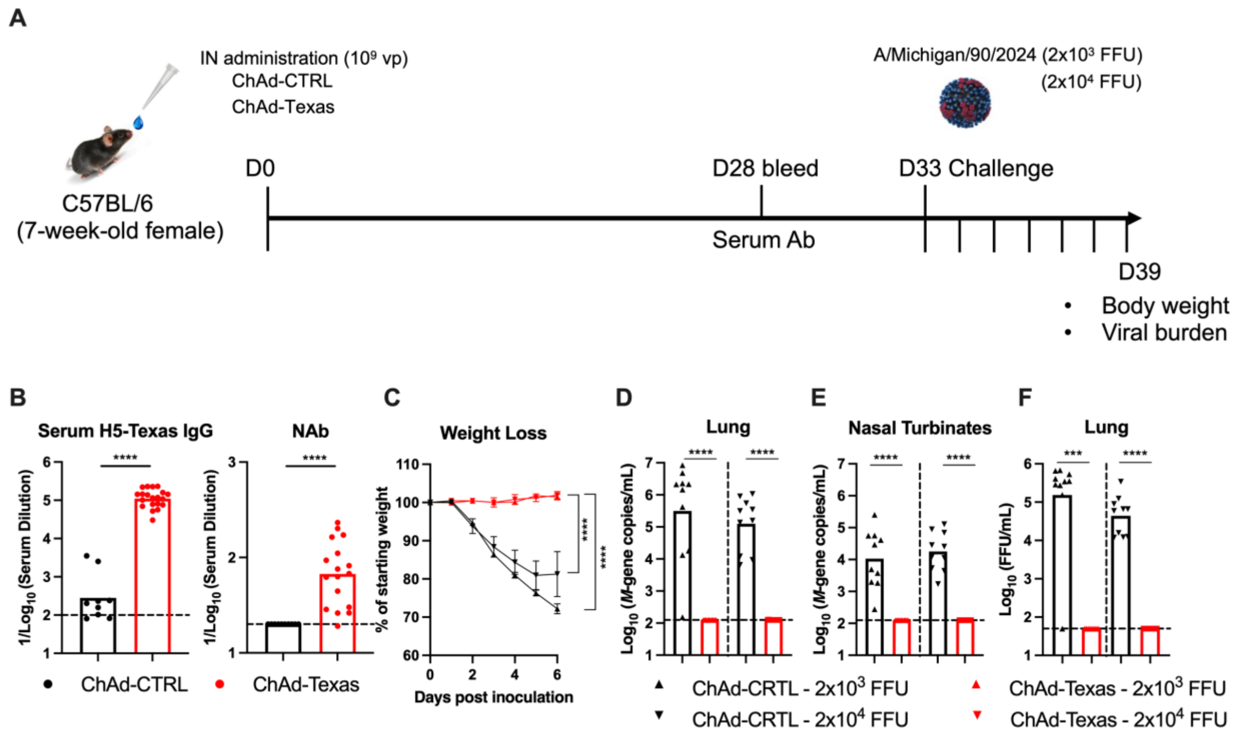

Figure S5. IN delivered ChAd-Texas vaccine confers protection against high dose H5N1 challenge, Related to Figure 4. (**A**) Scheme of immunizations, blood collection, and virus challenge. Cohorts of C57B/L6 mice were immunized via an IN route with 10^9 vp of ChAd-CTRL or ChAd-Texas. Sera were collected four weeks after immunization, and H5-Texas specific IgG and neutralizing antibody titers were determined (**B**). Four to five weeks after immunization, mice were challenged IN with increasing doses (2,000 or 20,000 FFU) of H5N1 A/Michigan/90/2024**.** (**C**) Body weight change. Viral RNA levels were determined at 6 dpi in the lungs (**D**) and nasal turbinates (**E**). Infectious virus levels in the lung (**F**). n = 8-10, two experiments, boxes illustrate geometric mean values, dotted lines show the limit of detection. Statistical analysis: (**B, D-F**) Mann-Whitney test. (**C**) two-way ANOVA analysis between ChAd-CTRL and ChAd-Texas immunized mice at corresponding challenge dose: **** $P < 0.0001$.

Supplementary Figure S6

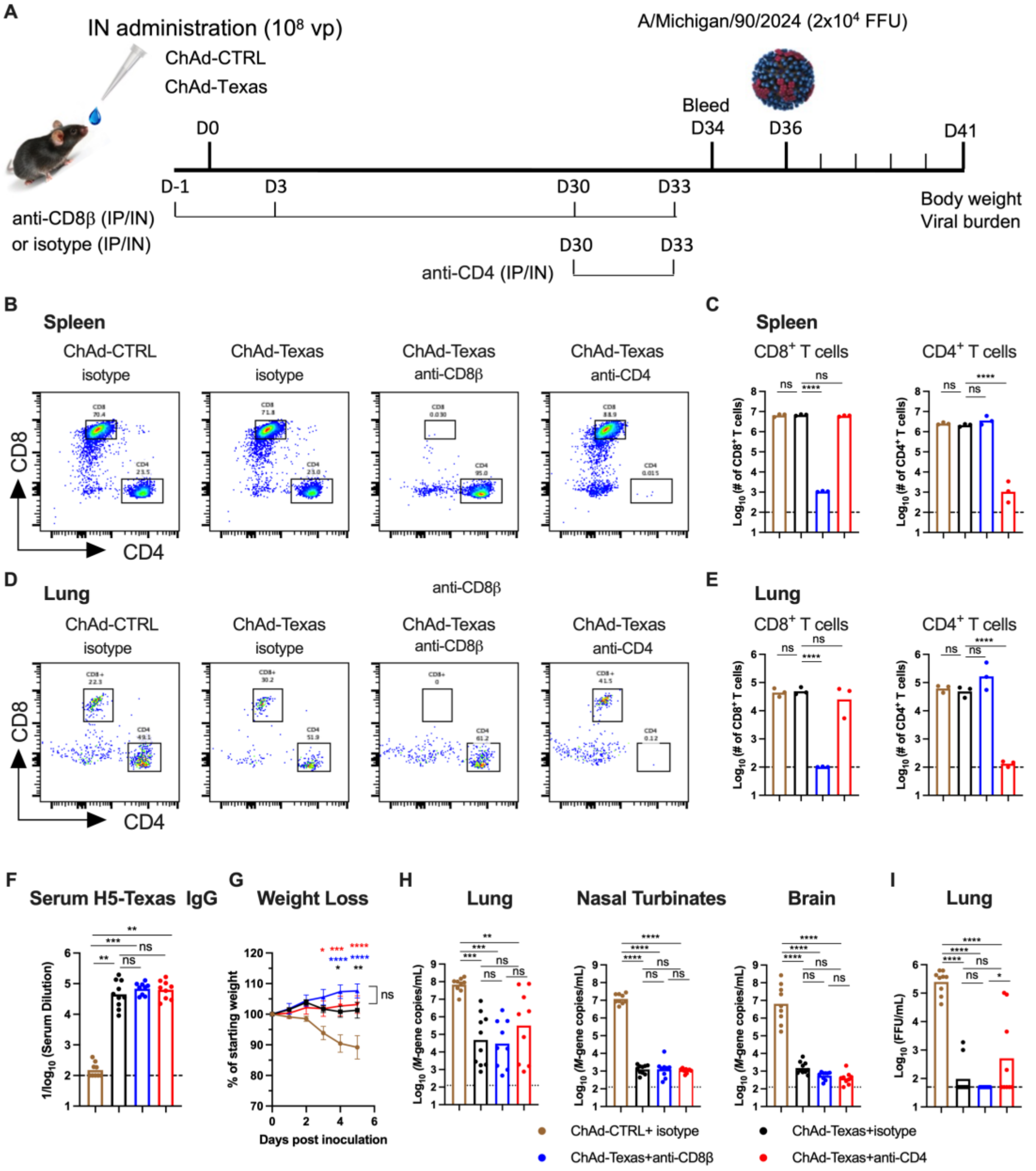

Figure S6. Depletion of CD4⁺ or CD8⁺ T cells had minimal impact on ChAd-Texas-mediated protection following H5N1 challenge, Related to Figures 3 and 4. (**A**) Scheme of immunizations, T cell depletion, and virus challenge. On day 34 post-immunization, spleens (**B-C**) and lungs (**D-E**) from a subset of animals were collected for analysis of $CD8^+$ and $CD4^+$ T cell frequencies and numbers by flow cytometry. Representative scatter plots (**B, D**) and total cell numbers (**C, E**) of $CD8^+$ and $CD4^+$ T cells in the spleen (**B-C**) and lungs (**D-E**) are shown (n = 3 per group; boxes indicate geometric mean values). (**F**) Serum IgG against HA of A/Texas/37/2024. (**G**) Weight loss following intranasal challenge with A/Michigan/90/2024. (**H**) Viral RNA levels in lungs, nasal turbinates, and brain. (**I**) Infectious virus titers in lungs (**F-I**, n = 8-10; two independent experiments). Boxes indicate geometric mean values; dotted lines represent the limit of detection. Statistical analysis: (**C, E**) one-way ANOVA with Dunnett's post-test; (**F**) Kruskal-Wallis with Dunn's test; (**G**) two-way ANOVA with Turke's post-test; (**H** and **I**) one-way ANOVA with Holm-Sidak post-test. ns, not significant, * $P < 0.05$; ** $P < 0.01$; *** $P < 0.001$; **** $P < 0.0001$.

Supplementary Figure S7

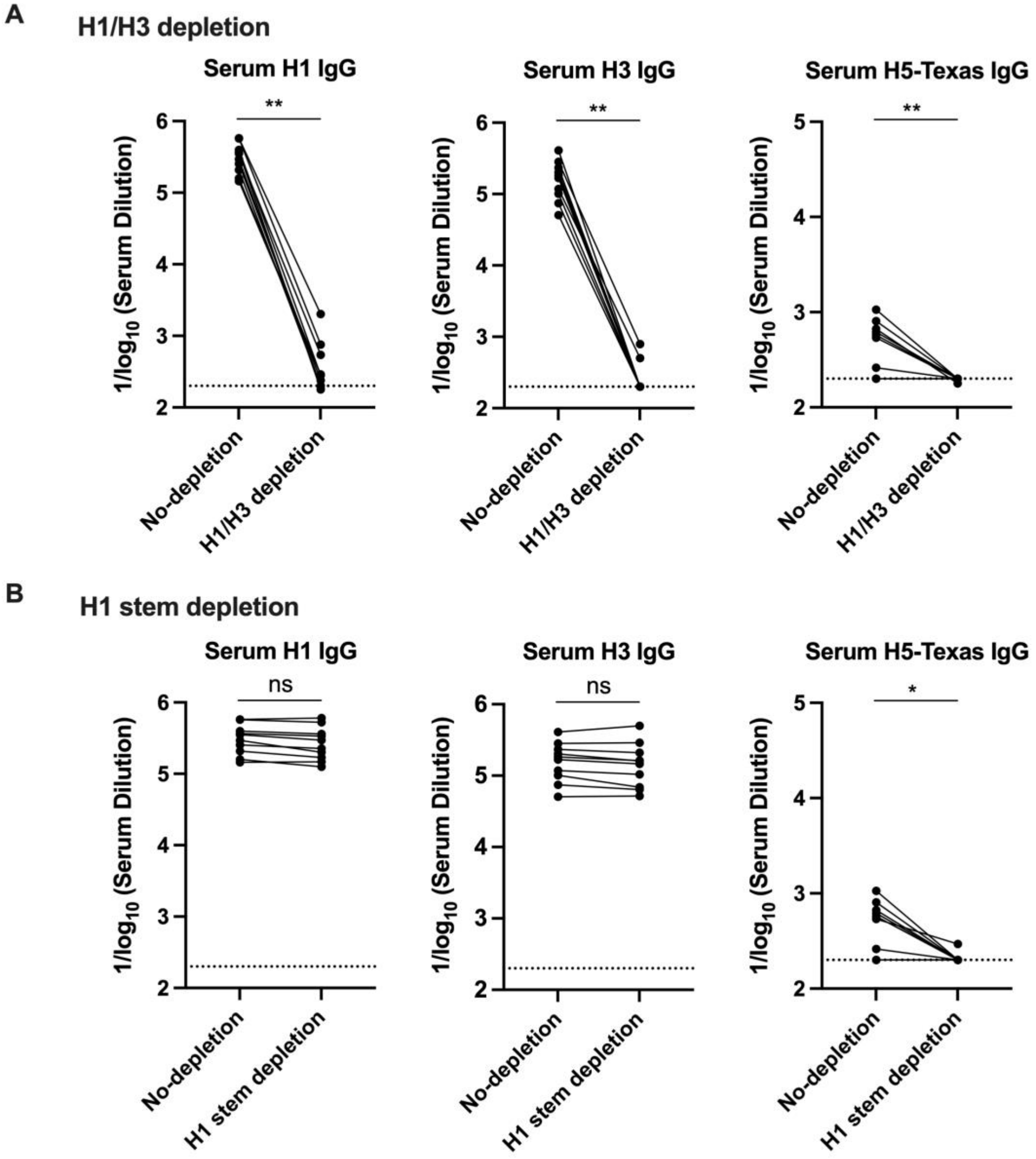

Figure S7. QIV immunization elicits low levels of H5-HA cross-reactive antibodies, Related to Figure 6. Serum samples collected three weeks after the second QIV immunization were absorbed with streptavidin-coated magnetic beads loaded with biotinylated HA proteins from H1, H3, or the stem domain of H1 HA. The pre-cleared sera were evaluated for residual binding to H1-, H3-, and H5-HA detection beads. Serum IgG levels binding to H1-HA, H3-HA, or H5-HA following pre-clearing with empty beads (no depletion), H1 and H3-HA loaded beads (H1/H3 depletion, panel **A**), or H1 HA stem–loaded beads (H1 stem depletion, panel **B**). Connecting lines represent sera from the same animals. Dotted lines indicate the limit of detection (LOD); values at the LOD are plotted slightly below the line. Wilcoxon matched pairs signed rank test. ns, not significant; * $P < 0.05$; ** $P < 0.01$.

Supplementary Figure S8

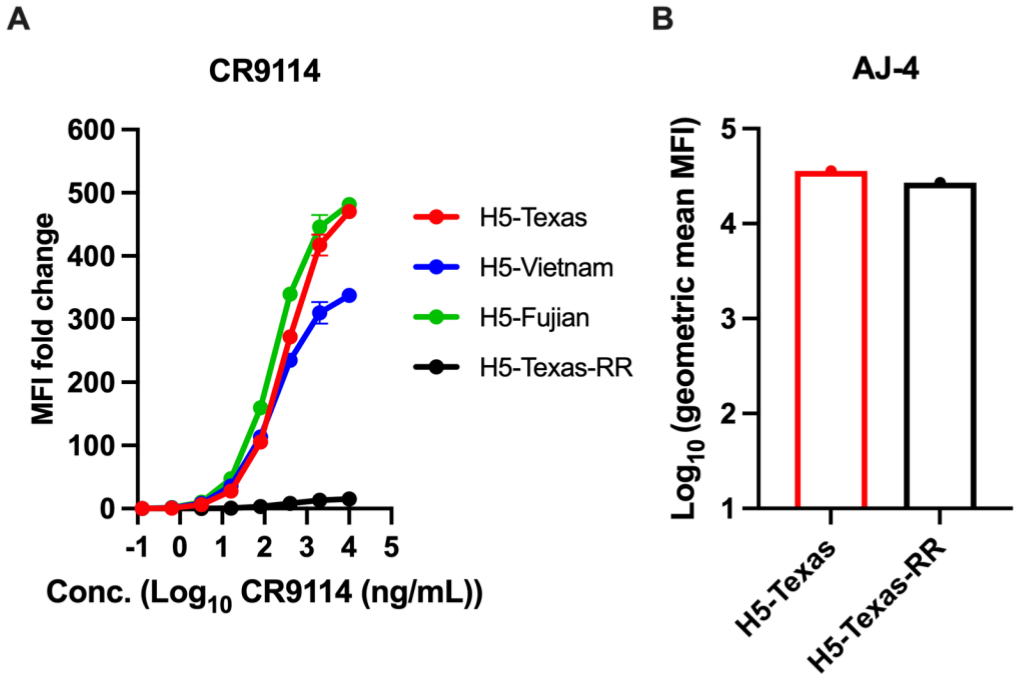

Figure S8. Elimination of the stem epitope in H5 HA, Related to Figure 6. Substitutions at residues 45 and 49 in the stem-domain of H5 HA (I45R-T49R) disrupt CR9114 binding as measured by flow-cytometry (**A**). Anti-H5 antibody (AJ-4), targeting the head domain of H5 was used to control for H5 HA binding (**B**).

Supplementary Figure S9

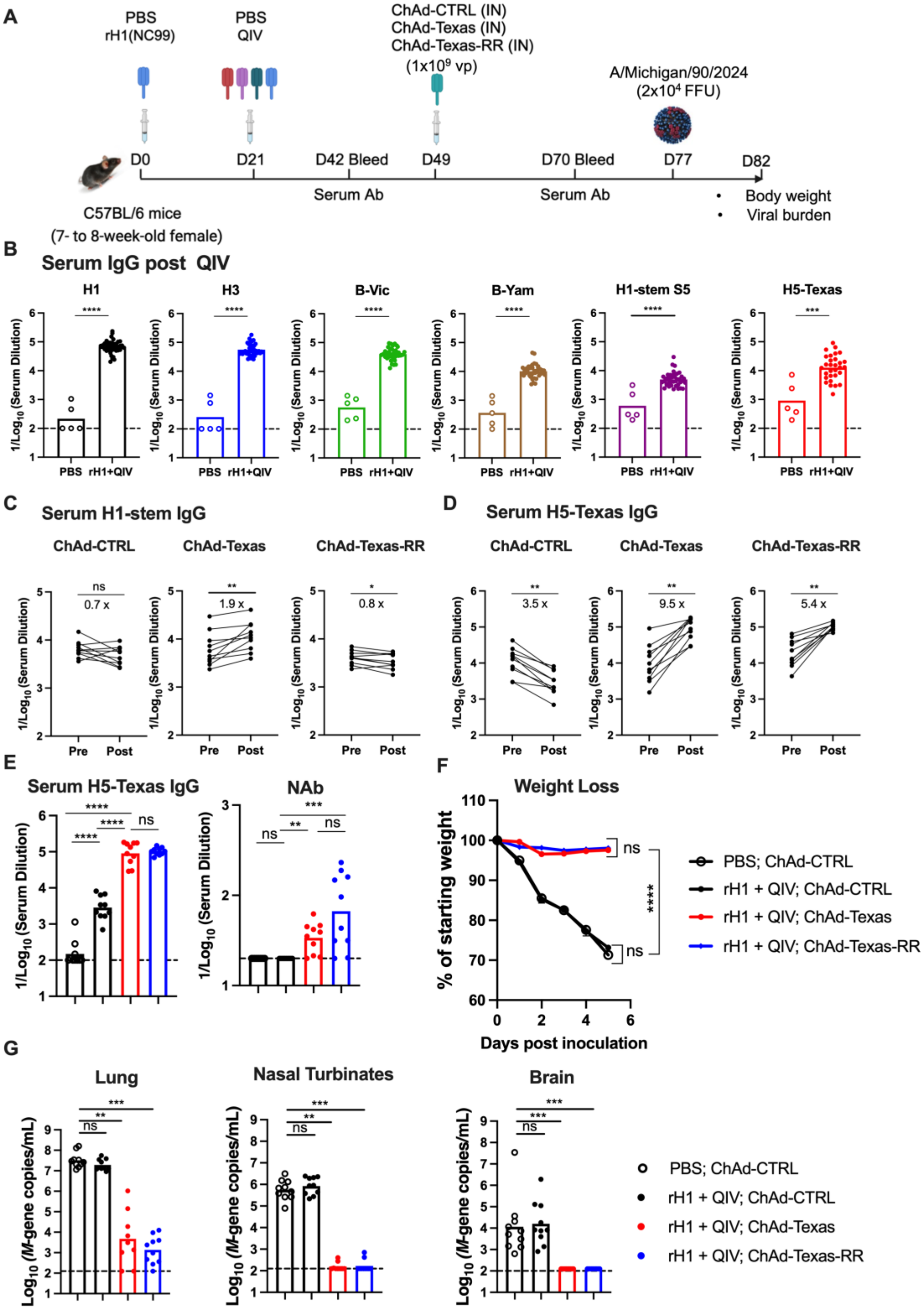

Figure S9. ChAd-Texas induces H5-specific immunity and confers protection against H5N1 in mice immunized with H1 and H3 HA antigens, Related to Figure 6. (**A**) Schematic of study design with indicated timeline of immunization, blood collection, and viral challenge. C57BL/6 mice were immunized IM with either PBS or 3.0 µg of recombinant H1 HA protein (A/New Caledonia/20/1999, H1N1) mixed 1:1 with AddaVax followed by seasonal QIV. Three weeks after the QIV dose, mice received an IN immunization of 10^9 vp of ChAd-CTRL, ChAd-Texas, or ChAd-Texas-RR vaccine. Serum samples were collected three weeks after QIV vaccine and three weeks after the ChAd-vaccine boost to evaluate antibody responses. (**B**) Serum IgG against HA of seasonal influenza virus strain and H5N1-Texas at day 21 after QIV immunization. (**C**) Paired analysis of serum IgG titers of pre- and post-boost with indicated ChAd-vaccine against H1 HA-stem protein. (**D**) Paired analysis of serum IgG titers pre- and post-boost with indicated ChAd-vaccine against H5 HA-Texas. (**E**) Serum H5-HA binding IgG and neutralizing antibody titers after ChAd-vaccine boost in previously unprimed or QIV primed mice. (**F**) Weight loss after IN challenge with 20,000 FFU of A/Michigan/90/2024. (**G**) Viral RNA levels determined at 4 dpi in lungs, nasal turbinates and brain. n = 9-10 mice per group, two independent experiments. Column heights indicate geometric mean values, and dotted lines show the limit of detection. Fold-changes are indicated above corresponding graphs. Statistical analyses: Mann–Whitney test (**B, E and G**); (**C-D**) Wilcoxon matched signed-rank test. (**F**) two-way ANOVA or mixed effect analysis with the Geisser-Greenhouse correction. ns, not significant; * $P < 0.05$, ** $P < 0.01$, *** $P < 0.001$, **** $P < 0.0001$.

Supplementary Table S1: HA protein sequences, Related to Figure 1, 3, 4, 5 and 6.

H1 A/Delaware/55/2019

MKAILVVMLYTFTTANADTLCIGYHANNSTDTVDTVLEKNVTVTHSVNLLEDKHNGKLCKLRGVA
PLHLGKCNIAGWILGNPECESLSTARSWSYIVETSNSDNGTCYPGDFINYEELREQLSSVSSFE
RFEIFPKTSSWPNHDSDNGVTAACPHAGAKSFYKNLIWLVKKGKSYPKINQTYINDKGKEVLVL
WGIHHPPTIADQQSLYQNADAYVFVGTSRYSKMFKPEIATRPKVRDQEGRMNYYWTLVEPGDK
ITFEATGNLVAPRYAFTMERDAGSGIIISDTPVHDCNTTCQTPEGAINTSLPFQNVHPITIGKCPKY
VKSTKLRLATGLRNVPSIQSRGLFGAIAGFIEGGWTGMVDGWYGYHHQNEQGSGYAADLKST
QNAIDKITNKVNSVIEKMNTQFTAVGKEFNHLEKRIENLNKKVDDGFLDIWTYNAELLVLLENERT
LDYHDSNVKNLYEKVRNQLKNNAKEIGNGCFEFYHKCDNTCMESVKNGTYDYPKYSEEAKLN
REKIDGGYIPEAPRDGQAYVRKDGEWVLLSTFLGSGLNDIFEAQKIEWHEGHHHHHH

H3 A/Darwin/11/2021

MKTIIALSNILCLVFAQKIPGNDNSTATLCLGHHAVPNGTIVKTITNDRIEVTNATELVQNSSIGEIC
DSPHQILDGGNCTLIDALLGDPQCDGFQNKEWDLFVERSRANSNCYPYDVPDYASLRSLVASS
GTLEFKNESFNWTGVKQNGTSSACIRGSSSSFFSRLNWLTSLNNIYPAQNVTMPNKEQFDKLYI
WGVHHPDTDKNQISLFAQSSGRITVSTKRSQQAVIPNIGSRPRIRDIPSRISIYWTIVKPGDILLIN
STGNLIAPRGYFKIRSGKSSIMRSDAPIGKCKSECITPNGSIPNDKPFQNVNRITYGACPRYVKQ
STLKLATGMRNVPEKQTRGIFGAIAGFIENGWEGMVDGWYGFRHQNSEGRGQAADLKSTQAA
IDQINGKLNRLIGKTNEKFHQIEKEFSEVEGRVQDLEKYVEDTKIDLWSYNAELLVALENQHTIDL
TDSEMNKLFEKTKKQLRENAEDMGNGCFKIYHKCDNACIGSIRNETYDHNVYRDEALNNRFQI
KGVGYIPEAPRDGQAYVRKDGEWVLLSTFLGSGLNDIFEAQKIEWHEGHHHHHH

Victoria-lineage B/Singapore/WUH4618/2021

MKAIIVLLMVVTSNADRICTGITSSNSPHVVKTATQGEVNVTGVIPLTTTPTKSHFANLKGTETRG
KLCPKCLNCTDLDVALGRPKCTGKIPSARVSILHEVRPVTSGCFPIMHDRTKIRQLPNLLRGYEH
VRLSTHNVINTEDAPGGPYEIGTSGSCLNITNGKGFFATMAWAVPKNKTATNPLTIEVPYICTEEE
DQITVWGFHSDDETQMVRLYGDSKPQKFTSSANGVTTHYVSQIGGFPNQTEDGGLPQSGRIV
VDYMVQKSGKTGTITYQRGILLPQKVWCASGKSKVIKGSLPLIGEADCLHEKYGGLNKSKPYYT
GEHAKAIGNCPIWVKTPLKLANGTKYRPPAKLLKERGFFGAIAGFLEGGWEGMIAGWHGYTSH
GAHGVAVAADLKSTQEAINKITKNLNSLSELEVKNLQRLSGAMDELHNEILELDEKVDDLRADTIS
SQIELAVLLSNEGIINSEDEHLLALERKLKKMLGPSAVEIGNGCFETKHKCNQTCLDRIAAGTFDA
GEFSLPTFDSLNITAASGYIPEAPRDGQAYVRKDGEWVLLSTFLGSGLNDIFEAQKIEWHEGHH
HHHH

Yamagata-lineage B/Singapore/INFTT-16-0610/2016

MKAIIVLLMVVTSNADRICTGITSSNSPHVVKTATQGEVNVTGVIPLTTTPTKSYFANLKGTRTRG
KLCPDCLNCTDLDVALGRPMCVGTTPSAKASILHEVRPVTSGCFPIMHDRTKIRQLPNLLRGYE
KIRLSTQNVIDAEKAPGGPYRLGTSGSCPNATSKIGFFSTMAWAVPKDNYKNATNPQTVEVPYI
CTEGEDQITVWGFHSDNKTQMKSLYGDSNPQKFTSSANGVTTHYVSQIGDFPDQTEDGGLPQ
SGRIVVDYMVQKPGKTGTIVYQRGVLLPQKVWCASGRSKVIKGSLPLIGEADCLHEEYGGLNK
SKPYYTGKHAKAIGNCPIWVKTPLKLANGTKYRPPAKLLKERGFFGAIAGFLEGGWEGMIAGW
HGYTSHGAHGVAVAADLKSTQEAINKITKNLNSLSELEVKNLQRLSGAMDELHNEILELDEKVDD
LRADTISSQIELAVLLSNEGIINSEDEHLLALERKLKKMLGPSAVDIGNGCFETKHKCNQTCLDRI
AAGTFNAGEYSLPTFDSLNITAASGYIPEAPRDGQAYVRKDGEWVLLSTFLGSGLNDIFEAQKIE
WHEGHHHHHH

H5 A/Vietnam/KhanhhoaRV1-005/2024

MERIVLLFATINLVKSDHICIGYHANNSTEQVDTIMEKNVTVTQAQDILEKTHNGKLCDLNGVKPL
ILKDCSVAGWLLGNPLCDEFINVPEWSYIVEKANPANDLCYPGNFNDYEELKHLLSRINHFEKIQ
IIPKNSWSDHEASLGVSAACSYQGNSSFFRNVVWLIKKNNAYPTIKKDYNNTNREDLLILWGIHH
PNDEAEQTKLYQNPSTYISIGTSTLNQRLVPKIATRPKINGQSGRIDFFWTILKPNDAIHFESNGN
FIAPEYAYKIVKKGDSTIMRSEAQYGNCNTRCQTPIGAINSSMPFHNIHPLTIGECPKYVKSSKLV
LATGLRNSPQREGRRKRGLFGAIAGFIEGGWQGMVDGWYGYHHSNEQGSGYAADKESTQKA
IDGVTNKVNSIIDKMNTQFEAVGREFNNLERRIENLNKKMEDGFLDVWTYNAELLVLMENERTL
DFHDSNVKNLYDKVRLQLKDNAKELGNGCFEFYHKCNNECMESVRNGTYDYPRYSEEARLKR
EEISGVGYIPEAPRDGQAYVRKDGEWVLLSTFLGHHHHHH

H5 A/Texas/37/2024

MENIVLLLAIVSLVKSDQICIGYHANNSTEQVDTIMEKNVTVTHAQDILEKTHNGKLCDLNGVKPL
ILKDCSVAGWLLGNPMCDEFIRVPEWSYIVERANPANDLCYPGSLNDYEELKHMLSRINHFEKI
QIIPKSSWPNHETSLGVSAACPYQGAPSFFRNVVWLIKKNDAYPTIKISYNNTNREDLLILWGIH
HSNNAEEQTNLYKNPITYISVGTSTLNQRLAPKIATRSQVNGQRGRMDFFWTILKPDDAIHFES
NGNFIAPEYAYKIVKKGDSTIMKSGVEYGHCNTKCQTPVGAINSSMPFHNIHPLTIGECPKYVKS
NKLVLATGLRNSPLREKRRKRGLFGAIAGFIEGGWQGMVDGWYGYHHSNEQGSGYAADKES
TQKAIDGVTNKVNSIIDKMNTQFEAVGREFNNLERRIENLNKKMEDGFLDVWTYNAELLVLMEN
ERTLDFHDSNVKNLYDKVRLQLRDNAKELGNGCFEFYHKCDNECMESVRNGTYDYPQYSEEA
RLKREEISGVGYIPEAPRDGQAYVRKDGEWVLLSTFLGHHHHHH

H5 A/Texas/37/2024-I45R-T49R

MENIVLLLAIVSLVKSDQICIGYHANNSTEQVDTIMEKNVTVTHAQDILEKTHNGKLCDLNGVKPL
ILKDCSVAGWLLGNPMCDEFIRVPEWSYIVERANPANDLCYPGSLNDYEELKHMLSRINHFEKI
QIIPKSSWPNHETSLGVSAACPYQGAPSFFRNVVWLIKKNDAYPTIKISYNNTNREDLLILWGIH
HSNNAEEQTNLYKNPITYISVGTSTLNQRLAPKIATRSQVNGQRGRMDFFWTILKPDDAIHFES
NGNFIAPEYAYKIVKKGDSTIMKSGVEYGHCNTKCQTPVGAINSSMPFHNIHPLTIGECPKYVKS
NKLVLATGLRNSPLREKRRKRGLFGAIAGFIEGGWQGMVDGWYGYHHSNEQGSGYAADKES
TQKARDGVRNKVNSIIDKMNTQFEAVGREFNNLERRIENLNKKMEDGFLDVWTYNAELLVLME
NERTLDFHDSNVKNLYDKVRLQLRDNAKELGNGCFEFYHKCDNECMESVRNGTYDYPQYSEE
ARLKREEISGVGYIPEAPRDGQAYVRKDGEWVLLSTFLGHHHHHH

H5 A/Fujian/2/2024

MEKIVLLLSVVNLVKSDQICIGYHANNSTEQVDTIMEKNVTVTHAQDILEKTHNGKLCRLNGVKP
LILKDCSVAGWLLGNPMCDEFISVPEWSYIVERVNPANDLCYPGNLNDYEELKHLLSRINHFEKT
RIIPKNSWSNHTSSGVSAACPYQGNASFFRNVVWLTKKNDAYPTIKMSYNNTNKEDLLILWGIH
HSNSAEEQTKLYKNLITYVSVGTSTLNQRLVPKIATRSQVNGQSGRMDFFWTMLKPNDAIHFES
NGNFIAPEYAYKIIKTGDSTIMKSEIEYGHCNTKCQTPIGAINSSMPFHNIHPLTIGECPKYVKSNK
LVLATGLRNSPLRERRRKRGLFGAIAGFIEGGWQGMVDGWYGYHHSNEQGSGYAADRESTQ
KAIDGVTNKVNSIIDKMNTQFEAVGREFNNLERRIENLNKKMEDGFLDVWTYNAELLVLMENER
TLDFHDSNVKNLYDKVRLQLRDNAKELGNGCFEFYHKCDNECMESVRNGTYDYPQYSEEARL
KREEISGVGYIPEAPRDGQAYVRKDGEWVLLSTFLGHHHHHH

Signal peptide
Foldon
Avi-tag
6x histag

Supplementary Table S2, Related to Figure 3.

Predicted MHC class I HA peptides in C57BL/6 mice by NetMHCPan 4.1 EL

allele	start	end	length	peptide	score	percentile_rank
H-2-Db	303	313	11	SSMPFHNIHPL	0.925328	0.01
H-2-Db	136	145	10	SSWPNHETSL	0.826912	0.02
H-2-Db	304	313	10	SMPFHNIHPL	0.670663	0.04
H-2-Db	445	453	9	VLMENERTL	0.554872	0.06
H-2-Db	11	19	9	VSLVKSDQI	0.521853	0.07
H-2-Db	158	166	9	SFFRNVVWL	0.414976	0.11
H-2-Db	321	329	9	YVKSNKLVL	0.411489	0.11
H-2-Db	109	117	9	GSLNDYEEL	0.395589	0.12
H-2-Kb	303	310	8	SSMPFHNI	0.94821	0.01
H-2-Kb	411	418	8	VGREFNNL	0.835582	0.02
H-2-Kb	155	163	9	GAPSFFRNV	0.774067	0.03
H-2-Kb	264	272	9	IAPEYAYKI	0.595342	0.07
H-2-Kb	208	216	9	KNPITYISV	0.415406	0.18
H-2-Kb	525	532	8	SVGTYQIL	0.279533	0.31
H-2-Kb	156	163	8	APSFFRNV	0.267409	0.33
H-2-Kb	137	145	9	SWPNHETSL	0.263779	0.33

Predicted MHC class II HA peptides in C57BL/6 mice by NetMHCPan 4.1 EL

allele	start	end	length	peptide	score	percentile_rank
H-2-IAb	204	218	TNLYKNPITYISVGT	0.8314	0.29	204
H-2-IAb	168	182	KKNDAYPTIKISYNN	0.4958	1.8	168
H-2-IAb	90	104	PEWSYIVERANPAND	0.491	1.8	90
H-2-IAb	303	317	SSMPFHNIHPLTIGE	0.4054	2.5	303
H-2-IAb	452	466	TLDFHDSNVKNLYDK	0.3664	2.8	452
H-2-IAb	531	545	ILSIYSTAASSLALA	0.3492	3	531
H-2-IAb	298	312	VGAINSSMPFHNIHP	0.2841	3.9	298
H-2-IAb	210	224	PITYISVGTSTLNQR	0.2478	4.6	210
H-2-IAb	344	358	KRGLFGAIAGFIEGG	0.2372	4.7	344
H-2-IAb	364	378	DGWYGYHHSNEQGSG	0.2299	4.9	364
H-2-IAb	366	380	WYGYHHSNEQGSGYA	0.1943	5.6	366
H-2-IAb	498	512	RNGTYDYPQYSEEAR	0.1675	6.5	498
H-2-IAb	265	279	APEYAYKIVKKGDST	0.1453	7.3	265
H-2-IAb	37	51	EKNVTVTHAQDILEK	0.1368	7.6	37
